# Supplementary material for: Malaria and intestinal parasite co-infection and its association with anaemia among people living with HIV in Buea, Southwest Cameroon: A community-based retrospective cohort study
Source: PLoS One. 2021 Jan 22;16(1):e0245743. doi: 10.1371/journal.pone.0245743 (PMC7822292; doi:10.1371/journal.pone.0245743)
Supplement: S1 File — (DOCX) [file pone.0245743.s001.docx]

**QUESTIONNAIRE FOR SUBJECTS**

**Study Title:** **Malaria and intestinal parasite co-infection and its association with anaemia among people living with HIV in Buea, Southwest Cameroon: A community-based retrospective cohort study**

Patient’s identification code: ____________ Date of enrolment: ________________

**Socio-demographic data**

Sex: M F Age: __________

Occupation: __________________________

Quarter of residence: ___________________

Address/contact: _______________________

Marital status: Married Divorced Single Widow(er)

Level of education: No formal Primary Secondary Tertiary

**Clinical evaluation:**

Temperature ________ ^o^C

HIV status: _____________

Present signs and symptoms: __________________________________________________

Malaria Test result: ________________ Parasitaemia: ________________

Intestinal Parasite Test result: _______________ Parasitaemia: _______________

Are you taking antiretroviral therapy? Yes No

If yes, which year did you start taking them? _____________

ART regimen: ____________________

CD4 T cell count: _____________

Viral load: _________________

Hb value: ____________

**Malaria Prevention methods used**

1. Do you use insecticide Residual spray (IRS) to kill mosquitoes? Yes No
2. Do you sleep under a mosquito net? Yes No
