# Supplementary material for: Malaria and intestinal parasite co-infection and its association with anaemia among people living with HIV in Buea, Southwest Cameroon: A community-based retrospective cohort study
Source: PLoS One. 2021 Jan 22;16(1):e0245743. doi: 10.1371/journal.pone.0245743 (PMC7822292; doi:10.1371/journal.pone.0245743)

PLOS ONE

Malaria and intestinal parasite co-infection and its association with anaemia among people living with HIV in Buea, Southwest Cameroon: A community-based cross-sectional study

--Manuscript Draft--

**Manuscript Number:**	PONE-D-20-20708
**Article Type:**	Research Article
**Full Title:**	Malaria and intestinal parasite co-infection and its association with anaemia among people living with HIV in Buea, Southwest Cameroon: A community-based cross-sectional study
**Short Title:**	Malaria and Intestinal parasite co-infection and its association with anaemia in HIV patients.
**Corresponding Author:**	Sorelle Mekachie Sandie, MSc<br>University of Buea<br>Buea, CAMEROON
**Keywords:**	Malaria; anaemia; intestinal parasite; HIV, co-infection
**Abstract:**	Background: Both malaria and intestinal parasites are endemic in Cameroon, and their co-infection can be of great impact on anaemia among people living with HIV (PLWH). This community-based cross-sectional study determined the prevalence and impact of infections on anaemia in PLWH and HIV-negative individuals in Buea, Cameroon from March to August 2019.<br>Methods: The study population comprised of 190 PLWH and 216 consenting HIV negative individuals from the Buea community. Participants were examined clinically, the collected blood sample was used for malaria parasite (MP) detection and speciation, HIV diagnosis and haemoglobin (Hb) measurement while stool samples were examined for the detection of intestinal parasites (IPs). Proportions were compared using Pearson's Chi-square test and association of anaemia with indepent variables was evaluated using binary logistic regression analysis.<br>Results: Out of the 406 participants, MP, IPs and MP/IP co-infection prevalences were 15.5%, 13.0% and 3.0% respectively. When compared with HIV negative participants, PLWH had a higher prevalence of MP (16.3%, P = 0.17), IPs (23.7%, P ˂ 0.001) and MP/IPs co-infection (3.7%, P = 0.04). Participants with triple infections co-infected with HIV, MP and IP had a significantly lower mean haemoglobin value (10.6 ± 1.21 g/dL). When comparing single infections, the prevalence of anaemia was highest among those who had HIV only (79.3%) when compared with those infected with MP only (71.4%) and IPs only (25.0%). HIV positive patients had a significantly (P ˂ 0.001) higher prevalence of mild (56.8%), moderate (18.4) and severe (1.6%) anaemia when compared with HIV negative counterparts. The significant risk factors associated with anaemia included being febrile (P = 0.03), MP-infected only (P = 0.001), HIV-infected only (P < 0.001), having dual (P < 0.001) or triple-infections (P = 0.03).<br>Conclusion: Multiple infections exercerbate the health condition of PLWH. Routine medical check-ups among PLWH are recommended.
**Order of Authors:**	Sorelle Mekachie Sandie, MSc
	Irene Ule Ngole Sumbele
	Helen Mbufong Kuokuo Kimbi
	Martin Mih Tasah
**Additional Information:**	
**Question**	**Response**
**Financial Disclosure**<br><br>Enter a financial disclosure statement that describes the sources of funding for the	The author(s) received no specific funding for this work.

Powered by Editorial Manager® and ProduXion Manager® from Aries Systems Corporation

work included in this submission. Review the submission guidelines for detailed requirements. View published research articles from *PLOS ONE* for specific examples.<br><br>This statement is required for submission and **will appear in the published article** if the submission is accepted. Please make sure it is accurate.<br><br>**Unfunded studies**<br>Enter: *The author(s) received no specific funding for this work.*<br><br>**Funded studies**<br>Enter a statement with the following details:<br>• Initials of the authors who received each award<br>• Grant numbers awarded to each author<br>• The full name of each funder<br>• URL of each funder website<br>• Did the sponsors or funders play any role in the study design, data collection and analysis, decision to publish, or preparation of the manuscript?<br>• **NO** - Include this sentence at the end of your statement: *The funders had no role in study design, data collection and analysis, decision to publish, or preparation of the manuscript.*<br>• **YES** - Specify the role(s) played.<br><br>* typeset	
**Competing Interests**<br><br>Use the instructions below to enter a competing interest statement for this submission. On behalf of all authors, disclose any competing interests that could be perceived to bias this work—acknowledging all financial support and any other relevant financial or non-financial competing interests.<br><br>This statement **will appear in the published article** if the submission is accepted. Please make sure it is accurate. View published research articles from *PLOS ONE* for specific examples.	The authors have declared that no competing interests exist.

**NO authors have competing interests**<br><br>Enter: *The authors have declared that no competing interests exist.*<br><br>**Authors with competing interests**<br><br>Enter competing interest details beginning with this statement:<br><br>*I have read the journal's policy and the authors of this manuscript have the following competing interests: [insert competing interests here]*<br><br>* typeset	
**Ethics Statement**<br><br>Enter an ethics statement for this submission. This statement is required if the study involved:<br><br>• Human participants<br>• Human specimens or tissue<br>• Vertebrate animals or cephalopods<br>• Vertebrate embryos or tissues<br>• Field research<br><br>Write "N/A" if the submission does not require an ethics statement.<br><br>General guidance is provided below. Consult the submission guidelines for detailed instructions. **Make sure that all information entered here is included in the Methods section of the manuscript.**	Name: Institutional Review Board hosted by the Faculty of Health Sciences of the University of Buea<br>Approval number: 661-09<br>Form of consent: Written consent

**Format for specific study types**<br><br>**Human Subject Research (involving human participants and/or tissue)**<br>• Give the name of the institutional review board or ethics committee that approved the study<br>• Include the approval number and/or a statement indicating approval of this research<br>• Indicate the form of consent obtained (written/oral) or the reason that consent was not obtained (e.g. the data were analyzed anonymously)<br><br>**Animal Research (involving vertebrate animals, embryos or tissues)**<br>• Provide the name of the Institutional Animal Care and Use Committee (IACUC) or other relevant ethics board that reviewed the study protocol, and indicate whether they approved this research or granted a formal waiver of ethical approval<br>• Include an approval number if one was obtained<br>• If the study involved *non-human primates*, add *additional details* about animal welfare and steps taken to ameliorate suffering<br>• If anesthesia, euthanasia, or any kind of animal sacrifice is part of the study, include briefly which substances and/or methods were applied<br><br>**Field Research**<br><br>Include the following details if this study involves the collection of plant, animal, or other materials from a natural setting:<br>• Field permit number<br>• Name of the institution or relevant body that granted permission	
**Data Availability**<br><br>Authors are required to make all data underlying the findings described fully available, without restriction, and from the time of publication. PLOS allows rare exceptions to address legal and ethical concerns. See the PLOS Data Policy and FAQ for detailed information.	No - some restrictions will apply

A Data Availability Statement describing where the data can be found is required at submission. Your answers to this question constitute the Data Availability Statement and **will be published in the article**, if accepted.<br><br>**Important:** Stating 'data available on request from the author' is not sufficient. If your data are only available upon request, select 'No' for the first question and explain your exceptional situation in the text box.<br><br>Do the authors confirm that all data underlying the findings described in their manuscript are fully available without restriction?	
**Describe where the data may be found in full sentences. If you are copying our sample text, replace any instances of XXX with the appropriate details.**<br><br>• If the data are **held or will be held in a public repository**, include URLs, accession numbers or DOIs. If this information will only be available after acceptance, indicate this by ticking the box below. For example: *All XXX files are available from the XXX database (accession number(s) XXX, XXX.).*<br>• If the data are all contained **within the manuscript and/or Supporting Information files**, enter the following: *All relevant data are within the manuscript and its Supporting Information files.*<br>• If neither of these applies but you are able to provide **details of access elsewhere**, with or without limitations, please do so. For example:<br><br>*Data cannot be shared publicly because of [XXX]. Data are available from the XXX Institutional Data Access / Ethics Committee (contact via XXX) for researchers who meet the criteria for access to confidential data.*<br><br>*The data underlying the results presented in the study are available from (include the name of the third party*	All relevant data are within the manuscript

Powered by Editorial Manager® and ProduXion Manager® from Aries Systems Corporation

and contact information or URL).<br>• This text is appropriate if the data are owned by a third party and authors do not have permission to share the data.<br><br>* typeset	
Additional data availability information:	

Powered by Editorial Manager® and ProduXion Manager® from Aries Systems Corporation

Manuscript
Click here to access/download;Manuscript;Sandies manuscript 2020.docx

Malaria and intestinal parasite co-infection and its association with anaemia among people living with HIV in Buea, Southwest Cameroon: A community-based cross-sectional study

Sorelle Mekachie Sandie[1*], Irene Ule Ngole Sumbele[1, 2], Martin Mih Tasah[1] and Helen Kuokuo Kimbi[1,3]

1 Department of Zoology and Animal Physiology, University of Buea, Buea, Cameroon.

2 Department of Microbiology and Immunology, Cornell College of Veterinary Medicine, Ithaca, New York, USA.

3 Department of Medical Laboratory Science, The University of Bamenda, Bambili, Cameroon

*Corresponding author

E- mail: **sandiesorelle2@gmail.com** **(SMS)**

Abstract

Background: Both malaria and intestinal parasites are endemic in Cameroon, and their co-infection can be of great impact on anaemia among people living with HIV (PLWH). This community-based cross-sectional study determined the prevalence and impact of infections on anaemia in PLWH and HIV-negative individuals in Buea, Cameroon from March to August 2019.

Methods: The study population comprised of 190 PLWH and 216 consenting HIV negative individuals from the Buea community. Participants were examined clinically, the collected blood sample was used for malaria parasite (MP) detection and speciation, HIV diagnosis and haemoglobin (Hb) measurement while stool samples were examined for the detection of intestinal parasites (IPs). Proportions were compared using Pearson's Chi-square test and association of anaemia with indepent variables was evaluated using binary logistic regression analysis.

Results: Out of the 406 participants, MP, IPs and MP/IP co-infection prevalences were 15.5%, 13.0% and 3.0% respectively. When compared with HIV negative participants, PLWH had a higher prevalence of MP (16.3%, P = 0.17), IPs (23.7%, P < 0.001) and MP/IPs co-infection (3.7%, P = 0.04). Participants with triple infections co-infected with HIV, MP and IP had a significantly lower mean haemoglobin value (10.6 ± 1.21 g/dL). When comparing single infections, the prevalence of anaemia was highest among those who had HIV only (79.3%) when compared with those infected with MP only (71.4%) and IPs only (25.0%). HIV positive patients had a significantly (P < 0.001) higher prevalence of mild (56.8%), moderate (18.4) and severe (1.6%) anaemia when compared with HIV negative counterparts. The significant risk factors associated with anaemia included being febrile (P = 0.03), MP-infected only (P = 0.001), HIV-infected only (P < 0.001), having dual (P < 0.001) or triple-infections (P = 0.03).

Conclusion: Multiple infections exercerbate the health condition of PLWH. Routine medical check-ups among PLWH are recommended.

Background

Parasitic infections are a major cause of morbidity and mortality in Africa; especially in resource-limited tropical and sub-tropical regions like sub-Saharan Africa [1] where the majority of HIV/AIDS cases are concentrated. People living with HIV (PLWH) are threatened by a great number of diseases caused by different types of biological agents.

In 2018, there were roughly 1.7 million new HIV infections, which is an insignificant improvement when compared to 2017 (1.8 million) [2]. HIV infection is characterized by immunosuppression that exposes the infected individual to a variety of microbial and parasitic infections such as *Plasmodium*, *Mycobacterium tuberculosis* and intestinal parasitic infections [3]. These infections (opportunistic or not) are a serious threat to the health status of people living with HIV, especially in developing countries like Cameroon. In an attempt to achieve HIV epidemic control in 2021, the Cameroon Government has been implementing various strategies that led to an encouraging increase in antiretroviral therapy (ART) coverage (53%) and viral load (VL) suppression (44.7%) nationally in 2019 [4].

African countries still bear the largest burden of malaria morbidity (93%), with *Plasmodium falciparum* accounting for 99.7% of malaria cases in 2018 [5]. In 2018, WHO reported that

Cameroon featured the fourth country behind Nigeria, Democratic Republic of Congo and Mozambique with the highest malaria prevalence [6]. Many studies have consistently reported an association between HIV infection and malaria in Cameroon [7, 8, 9, 10, 11], with each region showing a varied prevalence of both malaria and HIV infection. HIV prevalence is approximately 3.7% [4] while malaria transmission is stable throughout the year, with an overall malaria test positivity rate of 62% registered in 2017 [12].

The burden of intestinal parasite infections (IPIs) is greatest in low-income areas and are major public health problems in certain areas of Sub-Saharan Africa. These IPIs are associated with a humid climate, unsanitary environments and poor socio-economic conditions. Parasites causing these IPIs are protozoans (*Cryptosporidium species, Cyclospora cayetanensis, Isospora belli, Microsporidia spp* and *Entamoeba histolytica*) and helminths (*Ascaris lumbricoides, Trichuris trichuiria*, hookworms, *Schistosoma mansoni* and *Taenia species*). *Cryptosporidium species, Cyclospora cayetanensis, Isospora belli*, and *Microsporidia spp* are often referred to as opportunistic parasites in people living with HIV because they cause great morbidity especially when CD4 T cell count falls below 200 cells/µl [13]. Various studies carried out among African adults living with HIV have reported IPI rates ranging from 10% to 45% [14, 15]. Majority of the research on the impact of IPIs on HIV progression in Cameroon, occurred before the wide availability of ART [16] hence, the need for further evaluation.

In Cameroon, PLWH face similar environmental realities as those not infected with HIV and so can easily get co-infected with combinations of intestinal and malaria parasites. Such co-infections may have considerable health consequences which could lead to severe clinical symptoms and

pathology in PLWH. Studies have shown that individuals with multiple infections have an increased risk of developing more frequent and severe diseases due to interactions among infecting parasite species [17-19].

Malaria and intestinal parasites in PLWH can both lead to severe haematological abnormalities. Although ART in combination with cotrimoxazole confers a level of immunity to infections in PLWH, it has been reported to have adverse effects on the red blood cell indices of these patients, thereby causing anaemia [20]. Although malaria and intestinal parasites, as well as ART, are known etiological factors in anaemia [11, 16, 21, 22], the extent to which their interaction might enhance the risk of anaemia merits further investigations. Previous studies have reported on the influence of malaria/HIV co-infection on the occurrence of anaemia [11, 19, 23]. The situation regarding PLWH co-infected with malaria and intestinal parasites in the area following the intensification of different control strategies such as free availability of ART and cotrimoxazole, access to viral load testing and community ART dispensation is unknown. Therefore, the present study was undertaken to determine the prevalence of malaria parasite and intestinal parasites in PLWH compared to HIV negative counterparts as well as to assess the effects of these infections on anaemia to provide relevant information to policymakers in the country.

Methods

Study area and participants

The study was carried out in the HIV Treatment and Care Centre at the Regional Hospital and different neighbourhoods in the Buea community. The study area has been described in detail by Sumbele et al [24]. The Buea Regional Hospital is the referral HIV treatment centre in Fako

Division serving the population and hence receives HIV positive patients daily from the various neighbourhoods for their monthly distribution of ART. With the presence of the Mount Cameroon, Buea is a major touristic site and has a business-friendly environment. In the national HIV prevalence ranking, the South West Region is the 6th Region with a prevalence of 3.6% which equates the national prevalence (3.7%) [25] as well as the second to the last Region (33.8%) with the least viral load suppression. Buea is an area of moderate malaria transmission, with a peak time for malaria transmission during and just after the rainy season (March to September) [26]. Its tropical climate provides a suitable environment for the development of the malaria vectors and this therefore, contributes to increased malaria transmission. There is also inadequate access to safe water in this region, predisposing the population to water-borne infections including IPIs.

Two categories of participants; PLWH at the HIV Treatment and Care Centre and HIV-negative individuals in different neighbourhoods were enrolled in the study. Only participants who signed the informed consent form and willingly accepted to be counselled and tested for malaria, IPIs and HIV were enrolled in the study. Participants on antibiotics, malaria treatment or antiparasitic agents two weeks before enrollment were excluded from the study.

Study design and sample size

This study was a community-based cross-sectional study carried out between April 2018–August 2019. HIV-negative participants included persons who consented to be screened for HIV during the process and were negative. They were enrolled through HIV outreach programs in their homes and offices in the Buea neighbourhoods (Molyko, Bonduma, Great Soppo, Government reserve area (GRA), Buea town, Bokwaongo, Bokova, Mile 18, Clerks and Federal quarters). The sample

size was determined using the formula $n=Z^2pq/d^2$ [27] where n represented the sample size evaluated, Z was 1.96, which is the standard normal deviate (for the 95% confidence interval, CI), p was 14.1% [11] and 82.6% [16], the prevalence of malaria and IPIs in PLWH and HIV negative participants respectively, q was 1-p and d was 0.05. The sample size calculated for malaria parasite prevalence was 186 while that for IPIs prevalence was 220 giving an optimum sample size of 406 participants for the study.

Data collection

Questionnaire

A pre-structured questionnaire was used to collect socio-demographic information such as age, sex, location of residence, level of education, marital status and occupation while clinical data such as ART usage, duration on treatment, ART regimen and viral load (VL) were collected from each HIV patient's file at the HIV Treatment and Care Centre. Health and preventive practices towards malaria and IPIs as well as behavioural habits regarding health conditions with a history of symptoms were recorded.

Sample collection and processing

Participants were instructed on how to collect stool sample in universal transparent faecal containers with a screw cap lid. After the provision of a stool sample, the participant's body temperature was measured using a digital thermometer and fever was defined as temperature ≥ 37.5^{o}C. Blood was collected under sterile conditions into a well-labelled Ethylenediaminetetraacetate (EDTA) tube for each participant for HIV screening and

confirmation, malaria diagnosis and haemoglobin (Hb) measurement. The collected samples were transported to the Malaria Research Laboratory of the University of Buea for further analysis.
On arrival in the laboratory, the stool samples were analysed macroscopically and preserved in 10% formalin. Formol-ether concentration technique and Modified Ziehl-Neelsen (ZN) methods [28] were performed for the detection of intestinal parasites (protozoans and helminths).

The blood sample was used to prepare thick and thin blood films on a labelled slide that was Giemsa-stained and analysed following standard procedures [28] by two experienced microscopists. The blood was also used for HIV rapid test (in the case of presumed HIV-negative participants) and Hb measurement. Viral loads were obtained from the HIV positive participant's file at the HIV Care Centre. Viral load < 50 copies /mL of blood was considered undetectable [29]. CD4T cell counts were determined using the BD FACS Count™ System (BD Biosciences, USA) as per the manufacturer's instructions. To assure the quality of the laboratory data, standard operating procedure for each test was followed. Reagents were checked for expiry date and prepared according to the manufacturer's instruction. The Determine Rapid Test Kit (Abott Laboratories, CO., Ltd. Minato-Ku, Tokyo Japan) was used to test the supposed HIV negative subjects for HIV-1 following the manufacturer's instruction.

Haemoglobin (Hb) measurement

The FBC analysis was carried out using the URIT-3300 automated haematology analyser (URIT Medical Electronic CO., LTD. Jiuhua Road, Gungxi, China), following the manufacturer's instructions. Haemoglobin concentration was obtained from the FBC results. Anaemia was defined as Hb concentrations below the World Health Organization (WHO) reference values

corresponding to age or gender [30]. Further classification was done to determine severe (Hb < 7g/dL), moderate (Hb between 7.0 and 10.0 g/dL) and mild anaemia (> 10 g/dL Hb < 11g/dL) cases [28].

Statistical Analysis

Data were analysed using IBM-Statistical Package for the Social Science (SPSS) version 20.0 software (SPSS Inc., Illinois, USA). Baseline characteristics were summarized as mean with 95% CI and compared using the independent *t*-test. Malaria and intestinal parasites data were summarized into means and standard deviation (SD) and percentages were used in the evaluation of the descriptive statistics. The significance of differences in prevalence was explored using Pearson's Chi-square while Analysis of Variance (ANOVA) and Student's t-test was used where appropriate to assess difference in group means. The association of anaemia with the independent variables was evaluated using binary logistic regression analysis. $P < 0.05$ was considered for statistical significance.

Ethical considerations

Using an information sheet, a brief talk was given to the participants in the English Language (and exceptionally in Pidgin English) and the participants were then invited to participate in the study by signing an informed consent form. In the case of children (≤ 15years), their parents signed proxy informed consent form on their behalf. Ethical clearance was obtained from the Institutional Review Board hosted by the Faculty of Health Sciences of the University of Buea (Reference Number: 661-09). For confidentiality purposes, patients were identified by special identification

numbers. Results of participants with parasitic infections in the neighbourhoods were referred to a clinician for their case management.

Results

Socio-demographic and clinical characteristics of the participants

Five hundred and eighty-nine (589) participants were approached to participate in the study, with 406 (68.9%) being successfully enrolled. Out of the 406 participants, 190 were HIV positive patients from the HIV Treatment and Care Centre and 216 were HIV-negative from the communities as shown in Fig 1.

Fig 1: Study flow diagram showing compliance of the participants.

Overall, the mean ± SD age of the participants was 31.2 ± 16.7 (range = 1 – 70) years. There were more females (68.5%) than males (31.5%). A greater proportion of the study population belonged to the > 40 years age group (31.0%) and the least to the 31–40 years age group (18.7%). Majority of the PLWH had primary level of education (66.7%), were self-employed (31.1%) and were single (62.0%). Among HIV negative participants, majority had university level of education (35.2%), were students (56.5%) and were single (63.0%) as revealed in Table 1. Less than half of the stusy population (45.6%) used ITNs as a preventive measure against malaria. A greater proportion of PLWH used ITNs (57.4%) more than the HIV negative counterparts (35.2%).

Table 1. Characteristics of the study population

Parameters	HIV Positive	HIV Negative	Overall

		**n (%)**	**n (%)**	**n (%)**
**Age groups (years)**	≤ 20	42 (22.1)	72 (33.3)	114 (28.1)
	21-30	20 (10.5)	70 (32.4)	90 (22.2)
	31-40	48 (25.3)	28 (13.0)	76 (18.7)
	> 40	80 (42.1)	46 (21.3)	126 (31.0)
**Mean ± SD age**		35.5±16.5	27.6±15.8	32.3±16.7
**Sex**	Female	140 (73.7)	138 (63.9)	278 (68.5)
	Male	50 (26.3)	78 (36.1)	128 (31.5)
**Educational Level**	No formal	6 (3.7)	18 (8.6)	24 (6.0)
	Primary	126 (66.3)	54 (25.7)	180 (45.1)
	Secondary	43 (22.6)	64 (30.5)	107 (26.8)
	Tertiary	14 (7.4)	74 (35.2)	88 (22.1)
**Occupation**	Student	48 (25.4)	122 (56.5)	171 (42.1)
	Unemployed	25 (13.2)	12 (5.2)	37 (9.1)
	Self-employed	59 (31.2)	28 (13.0)	87 (21.4)
	Employee	57 (30.2)	54 (25.0)	111 (27.3)
**Marital status**	Single	93 (62.0)	102 (63.0)	197(62.5
	Married	61 (38.0)	60 (37.0)	117(37.5)
**ITN usage**	Yes	109 (57.4)	76 (35.2)	185 (45.6)
	No	81 (42.6)	140 (64.8)	221 (54.4)

Axillary temperature measurement revealed 14.0% (57) of the general population were feverish, with the highest proportion (15.7%) among HIV negative participants. Malaria clinical signs (headache, joint pains, nausea, vomiting etc…) were less frequent among HIV negative participants than among PLWH (23.4%). Anaemia occurred in 57.1% (232) of the participants and PLWH had the highest occurrence (76.8%) when compared with their HIV negative counterparts. Among PLWH, half of the population (50.0%) had CD4 T cell count between 200-500 cells/µL of blood, the majority (97.9%) were on ART, 91.9% (171) were taking zidovudine-based ART and 77.5% (141) had attained viral suppression(< 50 copies/mL of blood) as shown in Table 2.

Table 2. Clinical characteristics and laboratory profile of the study population

**Parameters**		**HIV Positive n (%)**	**HIV Negative n (%)**	**Overall n (%)**
**Fever status**	Febrile	23 (12.1)	34 (15.7)	57 (14.0)
	Afebrile	167 (87.9)	182 (84.3)	349 (86.0)
**Mean temperature (SD) in ^{o}C**		37.1 (0.5)	37.1 (0.5)	37.1 (0.5)
**Malaria clinical signs**	Yes	39 (23.5)	32 (18.2)	71 (20.7)
	No	127 (76.5)	144 (81.8)	272 (79.3)
**Anaemic status**	Anaemic	146 (76.8)	86 (39.8)	232 (57.1)
	Non-anaemic	44 (23.2)	130 (60.2)	174(42.9)
**Mean Hb (SD)** in g/dL		11.1(1.5)	12.5 (2.1)	11.8 (1.9)
**CD4 T-cell count(cells/µL)**	< 200	15 (14.2)	-	15 (14.2)
	200 – 500	51 (48.1)	-	51 (48.1)
	>500	40 (37.7)	-	40 (37.7)
**Mean CD4 T cell count SD (cells/µL)**		466.0 (275.9)	-	466.0 (275.9)
**ART usage**	Yes	186 (97.9)	-	186 (97.9)
	No	4 (2.1)	-	4 (2.1)

**Zidovudine-based ART**	Yes	171 (91.9)	-	171 (91.9)
	No	15 (8.1)	-	15 (8.1)
**ART Duration (years)**	0	4 (2.1)		4 (2.1)
	< 1	36 (19.4)	-	36 (19.4)
	1 -3	61 (32.8)	-	61 (32.8)
	>3	89 (47.8)	-	89 (47.8)
**Viral load (RNA copies/ml)**	Not detectable (<50)	141 (77.5)	-	141 (77.5)
	≥ 50	41 (22.5)	-	41 (22.5)

Malaria parasite, intestinal parasite infection and MP/IPI co-infection prevalences

Out of the 406 participants, 63 (15.5%) were positive for MP with *Plasmodium falciparum* as the only species observed. MP prevalence was higher in HIV positive participants (16.3%) than those negative (14.8%) although not statistically significant (P = 0.39). Overall, 13.0% (65) of the participants were positive for IPIs with a higher occurrence of protozoa (10.4%) than helminths (3.8%). The most prevalent protozoa parasite was *Cryptosporidium* species while that of helminths was hookworm. The prevalence of IPs was significantly higher (χ^2 = 31.73, P < 0.001) among PLWH (23.7%) than in those negative (5.6%). Malaria/IPs co-infection prevalence was 2.7% (11) and was comparable (P = 0.20) among PLWH (3.7%) and HIV negative participants (1.9%) as shown in Fig. 2.

Fig 2: Prevalence of MP, IPIs and MP/IPIs co-infection in the study population

Infection categories, haemoglobin level and anaemia prevalence

Out of the 406 participants, 1.7% (7) had triple infection (MP, HIV nad IPIs), 9.4% (38), 5.9% (24) and 1.0% (4) were co-infected with IPIs/HIV, MP/HIV and MP/IPIs respectively. Equally, 29.8% (121) had HIV only, 6.9% (28) had malaria only and 2.0 (8) had IPIs only. The rest of the participants (43.3%, 176) were uninfected. As shown in Fig 3, there was a significant difference ($H = 68.24$, $P < 0.001$) when comparing mean haemoglobin values between the different infection categories; as those triple infected had the lowest mean haemoglobin value (10.55 ± 1.20) when compared with dual and single infected patients accordingly.

Fig. 3: Box and whisker plots showing mean haemoglobin levels by infection categories

The prevalence of anaemia in the study population was 57.1% (232) and PLWH had a significantly higher ($\chi^2 = 56.59$, $P < 0.001$) prevalence of anaemia (76.8%) than their HIV negative counterparts (39.8%). When comparing single infections, the prevalence of anaemia was highest among those who had HIV only (79.3%), followed by those infected with malaria parasite only (71.4%) and then those with IPs only (25.0%) and the different was statistically significant ($P = 0.002$). Participants infected with MP, HIV and IPs simultaneously were observed to have the highest prevalence of anaemia (85.7%) when compared with other co-infection categories as shown in table 3.

With respect to anaemia severity, HIV positive patients had a significantly ($P < 0.001$) higher prevalence of mild (56.8%), moderate (18.4) and severe (1.6%) anaemia when compared with HIV negative. Severity of anaemia with respect to infection categories is seen in table 3.

Table 3. Prevalence of anaemia, anaemia severity and mean (SD) Hb levels by infection categories

Infection categories	Number examined N	Prevalence of anaemia n (%)	Mean (SD) Hb (g/dL)	Prevalence of anaemia severity		
				Mild anaemia n (%)	Moderate anaemia n (%)	Severe anaemia n (%)
**HIV positive**	190	146 (76.8)	11.11 (1.54)	108 (56.8)	35 (18.5)	3 (1.6)
**HIV negative**	216	86 (39.8)	12.45 (2.06)	72 (33.3)	12 (5.6)	2 (0.9)
**Test statistic**	$\chi^2 = 56.59$, $P < 0.001^*$		U = 29316 $P < 0.001^*$	$\chi^2 = 58.74$, $P < 0.001^*$		
**HIV only**	121	96 (79.3)	11.06 (1.44)	72 (59.5)	23 (19.0)	1 (0.8)
**MP only**	28	20 (71.4)	11.57 (1.52)	18 (64.3)	2 (7.1)	0 (0.0)
**IPs only**	8	2 (25.0)	12.40 (1.52)	2 (25.0)	0 (0.0)	0 (0.0)
**Test statistic**	$\chi^2 = 12.12$, $P = 0.002^*$		H = 6.87 $P < 0.001^*$	$\chi^2 = 14.47$, $P = 0.02^*$		
**MP/HIV**	24	19 (79.2)	10.83 (1.81)	15 (62.5)	3 (12.5)	1 (4.2)
**HIV/IPs**	38	25 (65.8)	11.49 (1.68)	17 (44.7)	7 (18.4)	1 (2.6)
**MP/IPs**	4	2 (50.0)	11.35 (0.06)	2 (50.0)	0 (0.0)	0 (0.0)
**MP/HIV/IPs**	7	6 (85.7)	10.55 (1.20)	4 (57.1)	2 (28.6)	0 (0.0)
**Test statistic**	$\chi^2 = 2.88$, $P = 0.83$		H = 3.84 $P = 0.28$	$\chi^2 = 5.07$, $P = 0.82$		

Risk factors of anaemia

A multinomial regression analysis was used to determine the factors associated with anaemia. As shown in Table 4, being febrile (P = 0.03), MP-positive only (P = 0.001), HIV-positive only (P <

0.001), dual (P < 0.001) and triple-infections (P = 0.03) were significant risk factors of anaemia in the study population (Table 4).

Table 4: Risk factors associated with anaemia in the study population

Parameters	Odds ratio (OR)	95% CI	P-value
**[a]Age group (years)**			
**≤ 20**	0.844	0.504 – 1.414	0.52
**21 – 30**	0.557	0.322 – 0.963	0.03*
**31 – 40**	1.091	0.606 – 1.964	0.77
**[b]Male**	0.373	0.242 – 0.573	< 0.001*
**[c]Febrile**	1.920	1.050 – 3.525	0.03*
**[d]HIV only**	7.061	7.061 – 4.123	< 0.001*
**[e]MP only**	4.597	1.914 – 11.041	0.001*
**[f]IP only**	0.613	0.120 – 3.128	0.57
**[g]Dual infections**	4.229	2.299 – 7.778	< 0.001*
**[h]Triple infection**	11.032	1.299 – 93.719	0.03*
**[i] Suppressed Viral load**	0.752	0.30 – 1.83	0.53
**[j]Zidovudine-based ART**	0.591	0.41 – 4.84	0.59
**[k]CD4 Tcell count (cells/μL)**			
**< 200**	0.359	0.070 – 1.842	0.22
**200 – 500**	0.642	0.248 – 1.659	0.36

*Statistically significant P-value
[a]Age group: ≤ 20 years =1; 21 – 30 years =2; 30 – 40 years =3; ≥ 41 years =4
[b]Male =1; Female =2
[c]Febrile =1; Afebrile =2
[d]HIV only =1; non-HIV only =2
[e]MP only =1; non-MP only =2
[f]IP only =1; non-IP only =2
[g]Dual infections =1; Non-dual infections =2
[h]Triple infection =1; non-triple infection =2
[i]Suppressed viral load=1; unsuppressed viral load=2
[j]Zidovudine-based ART=1; non-zidovudine-based ART=2
[k]CD4 T cell count: <200 cells/μL=1; 200-500 cells/μL=2; >500 cells/μL=3

Discussion

Parasitic infections (especially malaria and intestinal parasites) are major public health concerns among PLWH in developing countries, particularly Sub-Saharan Africa which has been reported to have the highest burden of HIV [2]. This study was a community-based cross-sectional study carried out to determine the prevalence of malaria and intestinal parasites and its association with anaemia among PLWH and HIV negative individuals in Buea, in the South West Region of Cameroon.

In the present study, the overall prevalence of malaria parasite was 15.5%, irrespective of the HIV status of the participants. This result is lower than the recently reported national prevalence (24.4%) [31]. This decrease could be attributed to the restless efforts of the Cameroon Government over the years to implement malaria control strategies to curb down malaria morbidity and mortality on the entire territory; knowing that Cameroon comes fourth on the list of countries that account for the burden of malaria in the WHO African Region [6].

The prevalence of malaria parasite among PLWH was 16.3%, which is high compared to the prevalence reported in PLWH in other areas of the South West Region of Cameroon. This includes the 7.8% reported in Limbe [11] and 2.3% in Buea [32] in 2019 and 2018 respectively. It was also observed in this study that despite the efforts made to scale up insecticide-treated nets (ITNs) distribution so that universal coverage can be attained, coverage remains low (57.4%) and this could have contributed to this rise in malaria prevalence among PLWH. Another reason for this difference could be the difference in the age distribution of the study population.

The prevalence of malaria parasite was slightly higher in HIV positive patients than in their HIV negative counterparts in line with findings in studies carried out in Nigeria [33]. Interactions between malaria and HIV have been studied extensively, which consider HIV positive patients as a high-risk group for malaria in endemic areas like Cameroon [10, 11, 32, 34]. This slight difference is not significant and this could be as a result of the government actions through the availability of different ACTs and ITNs for both PLWH and HIV-negative Cameroonians; as well as the increasing availability and rapid scale-up of ART and cotrimoxazole to HIV patients in particular.

It is worth noting that the HIV negative participants in the communities with malaria parasite are asymptomatic carriers and could greatly fuel the transmission of the malaria parasite in the community. However, the low prevalence shows that strategies implemented by the Cameroon government over the years are effective as this prevalence is lower than the reported national prelence of malaria in 2018 (29%) [6].

Fever is one of the most common clinical signs and characteristic feature of both parasitic and microbial infections. In this study, the febrile status of most of the participants irrespective of their HIV status was attributed to the presence of malaria parasite. This is in line with other studies carried out in Cameroon [11] and in Gabon [35]. Also, feverish PLWH had the highest prevalence of MP/IPs co-infection asserting the fact that fever remains an important feature in the clinical diagnosis of infections.

The prevalence of IPs in Cameroon varied from 27.8% in 2012 [36], to 14.6% in 2013 [37] and 13.0% in this present study. Considering the HIV status, PLWH had a higher prevalence of IPs

(23.7%) when compared with their HIV negative counterparts (5.6%). This prevalence among PLWH is lower than the 59.52% [37] in 2013 and 57.48% [38] in 2014 reported among HIV patients in the Centre and West Regions of Cameroon respectively. This observed decrease in the prevalence of IPs among HIV patients may be accredited to the attainment of viral suppression by a majority (77.5%) as well as their better awareness of IPs and their causes. It may also be due to the improvement in the care provided to PLWH by the government of Cameroon by ensuring a constant supply and provision of ART free of charge.

This study revealed 3.7% of PLWH were co-infected with MP and IPs. Limited information is available on co-infection among PLWH in Cameroon; but when compared with other studies carried out among children [19, 39, 40], the prevalence is low. The mean age (35.5 ±16.5 years) of PLWH, as well as the undetectable viral load observed in the majority of these patients, could account for this low prevalence. The lower the viral load, the faster the patient's immune system will recover, increasing the chances of fighting any infection easily. Besides, the majority of these patients have been on ART regularly for more than a year which probably could have contributed in boosting their immune systems against these parasites.

PLWH had a higher prevalence of anaemia (76.8%) when compared with their HIV negative counterparts (39.8%). This prevalence is higher than the less than 56.0% reported in several studies carried out among PLWH [10, 11, 41-45,]. The reason for the observed difference might be due to the heterogeneity of the study population as these previously reported anaemia prevalences were those of children or adult HIV patients, while this study included both children and adult participants (1 – 70 years old). Malaria and HIV positivity, as well as fever, were identified in this

study as risk factors of anaemia and therefore contributed significantly to the high prevalence of anaemia. Mixed nutritional deficiencies (iron, folic acid, or vitamin B12), though not investigated in this study, could have contributed to this high prevalence of anaemia as reported by a study carried out by Volberding et al. [46]. A direct comparison of the prevalence of anaemia in different studies is difficult as the study population, inclusion and exclusion criteria and anaemia definitions were different.

Parasites like the malaria parasites and intestinal parasites have long been recognized as major contributors to reduced haemoglobin levels in endemic countries like Cameroon, thereby causing anaemia. Also, PLWH on Zidovudine-containing ART have been reported to influence the haemoglobin level in previous studies [10,11, 47-49]. Aside from well-reported side eff ects of anaemia associated with ZDV-containing ART regimen, though not confirmed in this study, anaemia among HIV infected individuals is more broadly multifactorial in origin, and three basic pathophysiologic mechanisms of anaemia in HIV infection have been postulated [46]: decreased RBC production, increased RBC destruction, and ineff ective RBC production. In this study, patients infected with HIV, malaria and IPs had a significantly lower mean haemoglobin value (10.6 ± 1.21 g/dL). This is inline with previous studies which reported though among children, low haemoglobin values in patients with co-infections when compared with those with single infections [50-51].

A high prevalence of severe anaemia was observed among patients co-infected with MP and HIV. This is consistent with studies carried out in Nigeria [52-53], Ethiopia [48] and more recently in Cameroon [11]. This confirms the possibility that co-infections may increase malaria morbidity,

further worsening malaria disease severity [54]. In addition, triple, dual, HIV and malaria parasite infections as well as fever were reported as risk factors of anaemia in this study.

Even though different MP and IP species have been reported in Cameroon among PLWH, limited knowledge exists on the occurrence of MP/IPS co-infection among PLWH. Findings of this study will fill some of the gaps although it has its limitations. Notwithstanding, the fact that ART-naïve HIV-infected patients were not included in the study, nutritional deficiency as a cause of anaemia was not investigated, as well as the cross-sectional nature of the study that provides a picture at a point in time, the findings are valuable to policymakers in guiding the management of these conditions. Future longitudinal studies on the impact of iron supplementation and vitamin B12 levels among PLWH may be of great relevance.

Conclusions

From this study, it can be concluded that malaria parasite and intestinal parasites remain a public health concern among PLWH with higher occurrences of both MP, IPs and MP/IP co-infections than in their HIV negative counterparts in the communities. Though a good majority of the HIV patients in the study had attained viral suppression, they are still more vulnerable to some of the parasites than HIV negative individuals and therefore can constitute a good source of contamination in their various communities. To achieve HIV epidemic control in 2021, the Cameroon Government needs to intensify the implementation of the various strategies. Anaemia stands as a serious haematological abnormality among HIV patients and gets exacerbated even with the viral load suppression in the population of PLWH. Hence, there is a need for constant monitoring of the health status as well as the nutritional status of this at-risk group to better manage them.

Acknowledgements

The authors thank all the participants (both children and adults) who took part in the study as well as to the staff at the Unit in-charge of patients with HIV (UPEC) in the Buea Regional Hospital. We are also thankful to the Buea community inhabitants who consented and parents who consented for their children to participate in the study.

Authors' Contributions

Conceptualization: Sorelle Mekachie Sandie.

Data curation: Irene Ule Ngole Sumbele.

Formal analysis: Sorelle Mekachie Sandie.

I**nvestigation:** Sorelle Mekachie Sandie, Irene Ule Ngole Sumbele, Martin Mih Tasah, Helen Kuokuo Kimbi.

Methodology: Sorelle Mekachie Sandie.

Supervision: Irene Ule Ngole Sumbele, Helen Kuokuo Kimbi.

Validation: Irene Ule Ngole Sumbele, Helen Kuokuo Kimbi.

Writing – original draft: Sorelle Mekachie Sandie.

Writing – review & editing: Irene Ule Ngole Sumbele, Helen Kuokuo Kimbi.

References

1. Bangert M. The cross-cutting contribution of the end of neglected tropical diseases to the sustainable development goals. Infectious Diseases of Poverty 2017; 6.1: 73.

2. UNAIDS: UNAIDS report on the global AIDS epidemic 2018, Geneva, Switzerland.

3. Country Operational Plan (COP), Cameroon 2019. Strategic Direction Summary. https://www.state.gov/wp-content/uploads/2019/09/Cameroon_COP19-Strategic-Directional-Summary_public.pdf Accessed 07 Apr 2020.

4. Mabbott, N. A. (2018). The Influence of Parasite Infections on Host Immunity to Co-infection With Other Pathogens. *Frontiers in Immunology*, *9*. https://doi.org/10.3389/fimmu.2018.02579

5. World Health Organization. World Malaria Report 2019. Cameroon, African Region https://www.who.int/malaria/publications/country-profiles/profile_cmr_en.pdf?ua=1 2019. Accessed 28 Feb 2020.

6. World Health Organization. World Malaria Report 2018. Cameroon, African Region https://www.who.int/malaria/publications/country-profiles/profile_cmr_en.pdf?ua=1 2018. Accessed 04 Mar 2020.

7. Nkuo-Akenji T, Tevoufouet EE, Nzang F, Fon E, Ebong IN. HIV/AIDS and malaria in pregnant women from Cameroon short running title: HIV, malaria in pregnancy. Afr J Health Sci. 2011;18:105-109.

8. Njunda LA, Kamga HLF, Nsagha DS, Assoh JCN, Kwenti TE. Low malaria prevalence in HIV-positive patients in Bamenda, Cameroon. J Microbiol Res. 2012;2:56-59.

9. Kimbi HK, Njoh DT, Ndamukong KJN,Lehman LG. Malaria in HIV/AIDS patients at different CD4 T cell levels in Limbe, Cameroon. J Bacteriol Parasitol. 2013;4 (164). doi: 10.4172/2155-9597.1000164.

10. Bate, B, Kimbi, HK., Lum, E. Lehman LG, Onyoh EF, Ndip LM, Njabi CM, Tonga C, Wempnje GB, Ndip RN, Bessong PO. Malaria infection and anaemia in HIV-infected children in

Mutengene, Southwest Cameroon: a cross sectional study. BMC Infect Dis. 2016;16,523. https://doi.org/10.1186/s12879-016-1853-z

11. Sandie, SM., Sumbele, IUN, Tasah, MM and Kimbi HK. Malaria parasite prevalence and Haematological parameters in HIV seropositive patients attending the regional hospital Limbe, Cameroon: a hospital-based cross-sectional study. BMC Infect Dis. 2019;19, 988 https://doi.org/10.1186/s12879-019-4629-4

12. Malaria Operational Plan (MOP) FY 2018 and FY 2019. President's Malaria Initiative Cameroon, 2019. https://www.pmi.gov/docs/default-source/default-document-library/malaria-operational-plans/fy-2018/fy-2018-cameroon-malaria-operational-plan.pdf?sfvrsn=5 Accessed: 07 Apr 2020.

13. Alemu G, Alelign D, Abossie A. Prevalence of Opportunistic Intestinal Parasites and Associated Factors among HIV Patients while Receiving ART at Arba Minch Hospital in South Ethiopia: A Cross-sectional Study. Ethiop J Health Sci. 2018; 28(2):147. doi:http://dx.doi.org/10.4314/ejhs.v28i2.6

14. Webb EL, Ekii AO, Pala P. Epidemiology and immunology of helminth = HIV interactions. Curr Opin HIV AIDS. 2012;7:245–53.

15. Adeleke OA, Yogeswaran P, Wright G. Intestinal helminth infections amongst HIV-infected adults in Mthatha General Hospital, South Africa. Afr J Prm Health Care Fam Med. 2015; 7(1). http://dx.doi.org/10.4102/phcfm.v7i1.910

16. Nsagha DS, Njunda AL, Assob NJC, Ayima CW, Tanue EA, kibu OD and Kwenti TE. Intestinal parasitic infections in relation to CD4+Tcell counts and diarrhea in HIV/AIDS patients with or without antiretroviral therapy in Cameroon. BMC Infectious Diseases. 2016;16:9. DOI 10.1186/s12879-016-1337-1

17. Nacher M, Singhasivanon P, Yimsamran S, Manibunyong W, Thanyavanich N, Wuthisen R. Intestinal helminth infections are associated with increased incidence of Plasmodium falciparum malaria in Thailand. J Parasitol. 2002;88(1):55–8.

18. Sokhna C, Le Hesran JY, Mbaye PA, Akiana J, Camara P, Diop M. Increased malaria attacks among children presenting concomitant infections by S. mansoni in Senegal. Malar J. 2004;3:43.

19. Njunda AL, Fon SG, Assob JCN, Nsagha DS, Kwenti TDB and Kwenti TE. Coinfection with malaria and intestinal parasites, and its association with anaemia in children in Cameroon. Infectious Disease of Poverty. 2015;4:43 DOI 10.1186/s40249-015-0078-5

20. Wagnew, F., Eshetie, S., Alebel, A, Tesema C, Kibret GD, Gebrie A, Dessie G and Abajobir AA. Burden of anemia and its association with HAART in HIV infected children in Ethiopia: a systematic review and meta-analysis. BMC Infect Dis. 2019;19 https://doi.org/10.1186/s12879-019-4656-1

21. Brooker S, Akhwale W, Pullan R, Estambale B, Clarke SE, Snow RW. Epidemiology of Plasmodium-helminth co-infection in Africa: populations at risk, potential impact on anemia and prospects for combining control. Am J Trop Med Hyg.2007;77(6S):88–98.

22. Sumbele, I. U., Nkemnji, G. B. and Kimbi, H. K. Soil-transmitted helminths and plasmodium falciparum malaria among individuals living in different agroecosystems in two rural communities in the mount Cameroon area: a cross-sectional study. Infectious diseases of poverty, 2017;6(1), 67. doi:10.1186/s40249-017-0266-6

23. Saracino A, Nacarapa EA, Massinga EAC, Martinelli D, Oliveira C, Antonich A, Galloni D, Ferro JJ, Macome CA and Scacchetti M. Prevalence and clinical features of HIV and malaria

co-infection in hospitalized adults in Beira, Mozambique. Malaria Journal 2012, 11:241 http://www.malariajournal.com/content/11/1/241

24. Sumbele IUN, Sama SO, Kimbi HK and Taiwe GS. Malaria, Moderate to Severe Anaemia, and Malarial Anaemia in Children at Presentation to Hospital in the Mount Cameroon Area: A Cross-Sectional Study. Anemia. 2016; Article ID 572

25. CAMPHIA (Cameroon Population-based HIV Impact Assessment) 2017. Summary sheet: Preliminary findings. https://phia.icap.columbia.edu/wp-content/uploads/2018/07/3471CAMPHIA_Cameroon-SS_A4 v13_requests_7.25.18.pdf 2017. Accessed 09 Apr 2020.

26. World Health Organization. Malaria Factsheets 2020. https://www.who.int/news-room/fact-sheets/detail/malaria 2020. Accessed 02 Apr 2020.

27. Bryan FJ. The Design and Analysis of Research Studies, University of Otago, Dunedin, New Zealand; Cambridge University Press, Cambridge, UK, 1992.

28. Cheesbrough M. District Laboratory Practice in Tropical Countries, 3Rd ed., part 1. United Kingdom: Cambridge University Press; 2014. p. 178–215.

29. UNAIDS. Undetectable = Untransmittable. Publich health and HIV load suppression. 2018.https://www.unaids.org/sites/default/files/media_asset/undetectable-untransmittable_en.pdf Accessed 09 Apr 2020.

30. WHO Nutritional anaemias: tools for effective prevention and control. Geneva, Switzerland: World Health Organization Publication; 2017. https://www.who.int/nutrition/publications/micronutrients/anaemias-tools-prevention-control/en/. Accessed 07 April 2020.

31. Minsante. XIième Journée mondiale de lutte contre le paludisme “prêt à vaincre le paludisme” Nous sommes la génération qui peut éliminer le paludisme. Dossier de Presse. Minsante. 2018:1–20.

32. Mbah-Mbole FG, Tufon KA, Meriki DH, Enow-Orock G, Mbah-Mbole P, Njunda LA, Wam Iwoi MD, Kwenti TE. Malaria and human immunodeficiency virus coinfection in febrile patients attending the Regional Hospital of Buea, Southwest region, Cameroon. Int J Adv Med Health Res. 2018;6:46-51

33. Sanyaolu AO, Fagbenro-Beyioku AF, Oyibo WA, Badaru OS, Onyeabor OS, Nnaemeka CI, Malaria and HIV co-infection and their effect on haemoglobin levels from three healthcare institutions in Lagos, southwest Nigeria. Afr Health Sci. 2013; 13(2):295-300 http://dx.doi.org/10.4314/ahs.v13i2.14

34. Njunda AL, Njumkeng C , Nsagha SD , Assob JCN and Kwenti, TE. The prevalence of malaria in people living with HIV in Yaounde, Cameroon. BMC Public Health. 2016; 16:964 DOI 10.1186/s12889-016-3647-z

35. Bouyou-Akotet M, Offouga C, Mawili-Mboumba D, Essola L, Madoungou B, Kombila M: Falciparum Malaria as an Emerging Cause of Fever in Adults Living in Gabon, Central Africa. BioMed Res Int. 2014; Article ID 351281. kahighttp://dx.doi.org/10.1155/2014/351281

36. Lehman LG, Kangam L, Nguepi E, Mbenoun ML and Bilong Bilong CF. Study of intestinal parasitic infections associated with HIV infection in Douala, Cameroon. Retrovirology. 2012;9 (Suppl 1): P48.

37. Nkenfou CN, Nana CT and Payne VK. Intestinal Parasitic Infections in HIV Infected and Non-Infected Patients in a Low HIV Prevalence Region, West- Cameroon. PLoS ONE. 2013;8(2): e57914. doi:10.1371/journal.pone.0057914

38. Vouking MZ, Enoka P, Tamo C V, Tadenfok C N.Prevalence of intestinal parasites among HIV patients at the Yaoundé Central Hospital, Cameroon. Pan African Medical Journal. 2014;18:136 DOI: 10.11604/pamj.2014.18.136.3052

39. Kwenti TE, Nkume FA, Tanjeko AT and Kwenti TDB. The Effect of Intestinal Parasitic Infection on the Clinical Outcome of Malaria in Coinfected Children in Cameroon. PLoS Negl Trop Dis. 2016; 10(4): e0004673. doi:10.1371/journal.pntd.0004673.

40. Angora KE, Djohan V, Konaté A, Kiki-Barro PC and Bedia-Tanoh AV. Uncomplicated Malaria and Intestinal Helminth Co-Infections among Schoolchildren in Abobo District, Abidjan (Côte d'Ivoire). J Infect Dis Epidemiol. 2019;5:064. doi.org/10.23937/2474-3658/1510064

41. Assefa M, Abegaz WE and Mulugeta Belay. Prevalence and correlates of anemia among HIV infected patients on highly active anti-retroviral therapy at Zewditu Memorial Hospital, Ethiopia. BMC Hematol. 2015; 15: 6.

42. Ako SE, Njunda LA, Akum EA, Benjamin PT, Assob JC. Hematological Related Disorders and Transfusion of HIV Patients on Highly Active Antiretroviral Therapy (HAART) in the South West Region of Cameroon: Hematological Monitory Parameters for HIV Follow-Up. J HIV Retrovirus.2018;4;1:5

43. Lai JL, Chen YH, Liu YM, Yuan JJ, Lin J, Huang AQ and Ye HH. Prevalence and risk factors of anaemia in hospitalised HIV-infected patients in southeast China: a retrospective study. Epidemiol Infect 2019; 147:e81.

44. Gebremedhin KB and Haye TB. Factors Associated with Anemia among People Living with HIV/AIDS Taking ART in Ethiopia. Hindawi Advances in Hematology. 2019; 8 https://doi.org/10.1155/2019/9614205

45. Ageru TA, Koyra MM, Gidebo KD, Abiso TL. Anemia and its associated factors among adult people living with human immunodeficiency virus at Wolaita Sodo University teaching referral hospital. PLoS ONE. 2019;14(10) https://doi.org/10.1371/journal.pone.0221853

46. Volberding PA, Levine AM, Dieterich D, Mildvan D, Mitsuyasu R, Saag M for the Anemia in HIV Working Group. Anemia in HIV Infection: Clinical impact and evidence-based management strategies. Clin Infect Dis. 2004;38:1454–63.

47. Blake M and Sherer R. Management of the adverse effects of antiretroviral therapy and medication adherence. Clin Infect Dis. 2000;30(Suppl2):S96–116. https://doi.org/10.1086/313859.

48. Ojurongbe O, Oyeniran O, Alli O, Taiwo S, Ojurongbe T, Olowe A, Opaleye O, Adeyeba O. Prevalence of Plasmodium falciparum Parasitaemia and its correlation with haematological parameters among HIV-Positive individuals in Nigeria. J Trop Med Hyg. 2014:Article ID 161284. https://doi.org/10.1155/2014/161284.

49. Breymann C. Iron deficiency anemia in pregnancy. Semin Hematol. 2015; 52(4):339–47.

50. Tay, S.C., Badu, K., Mensah, A.A. and Gbedema, SY. The prevalence of malaria among HIV seropositive individuals and the impact of the co- infection on their hemoglobin levels. Ann Clin Microbiol Antimicrob. 2015;14, 10. https://doi.org/10.1186/s12941-015-0064-6

51. Njua-Yafi C, Achidi EA, Anchang-Kimbi JK, Apinjoh TO, Mugri RN, Chi HF, Tata RB, Njumkeng C , Nkock EN and Nkuo-AkenjI T. Malaria, helminths, co-infection and anaemia in a cohort of children from Mutengene, south western Cameroon. Malar J. 2016; 15:69 DOI 10.1186/s12936-016-1111-2

52. Erhabor O, Babatunde S, and Uko KE. Some haematological parameters in Plasmodial parasitized HIV-infected Nigerians. Nigeria Journal of Medicine. 2006; 15(1): 52-55.

53. Etusim PE, Ihemanma CA, Nduka FO, P.E. M, Ukpai O. Comparative study on the haematological characteristics of malaria infected and malaria non-infected persons referred to art/HIV laboratory, Abia State University teaching hospital, aba, Abia state. J Sci Multidiscip Res. 2013;5(1):100–12.

54. Rattanapunya S, Kuesap J, Chaijaroenkul W, Rueangweerayut R, and Na-Bangchang K. Prevalence of malaria and HIV coinfection and influence of HIV infection on malaria disease severity in population residing in malaria endemic area along the Thai-Myanmar border. Acta Tropica. 2015:145: pp. 55-60

Click here to access/download;Figure;Fig1.tif

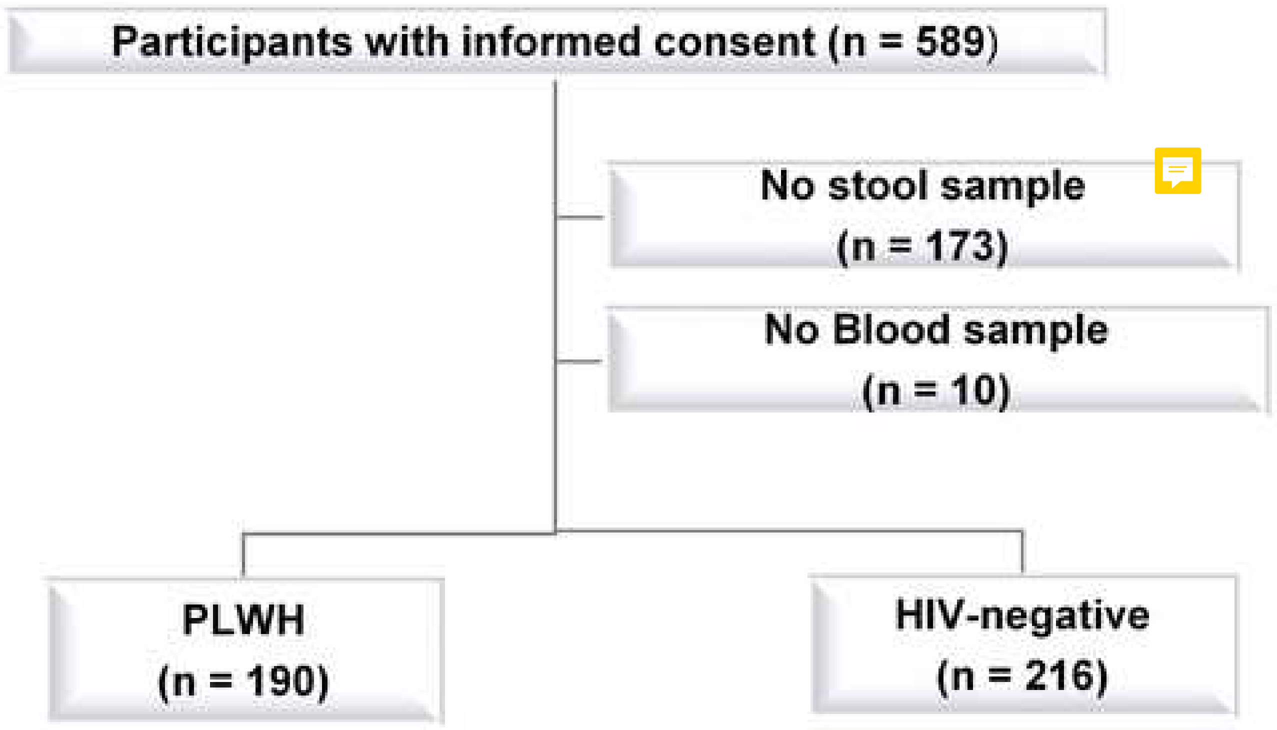

Figure

Click here to access/download;Figure;Fig2.tif

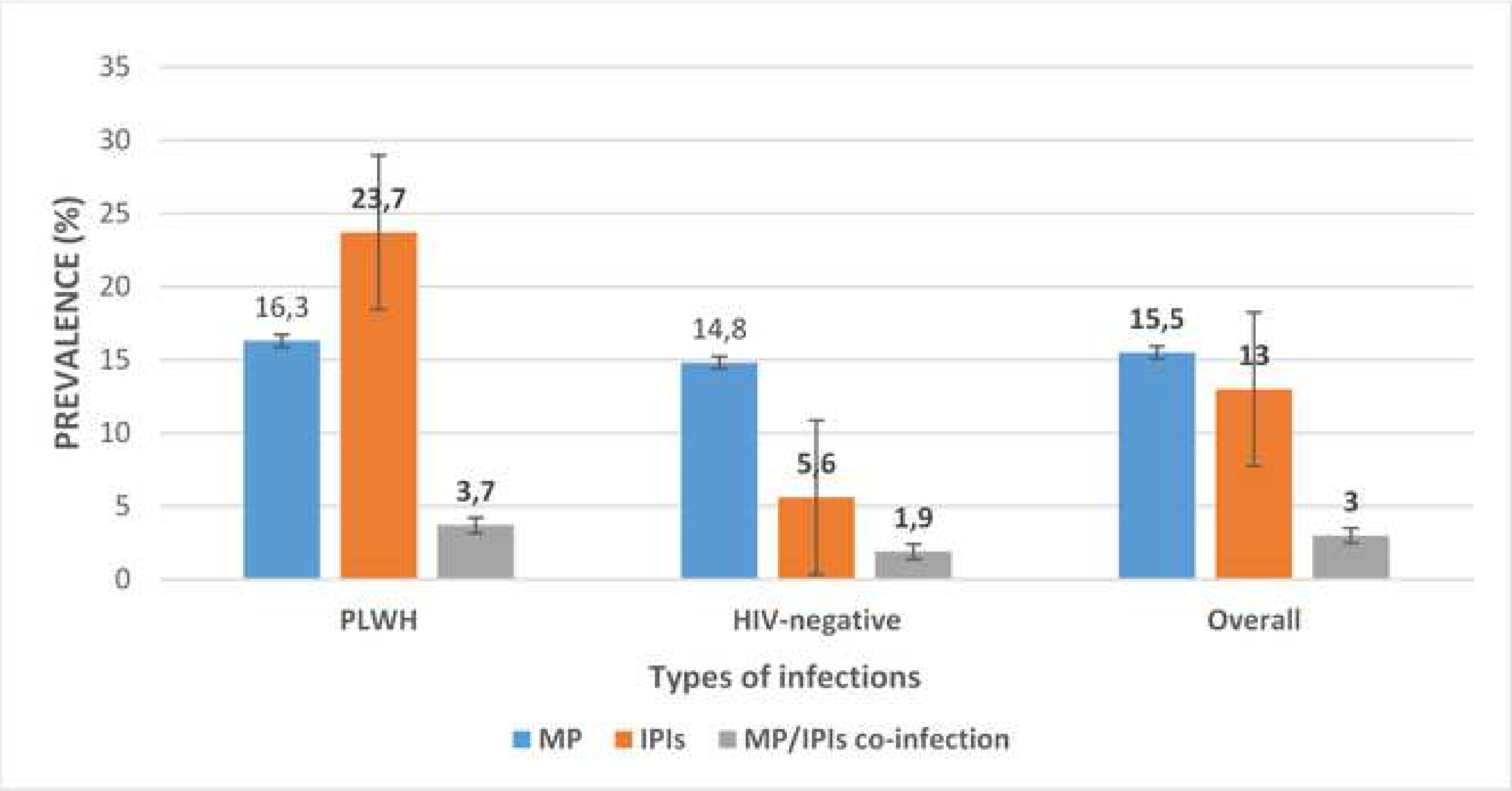

Figure

Click here to access/download;Figure;Fig3.tif

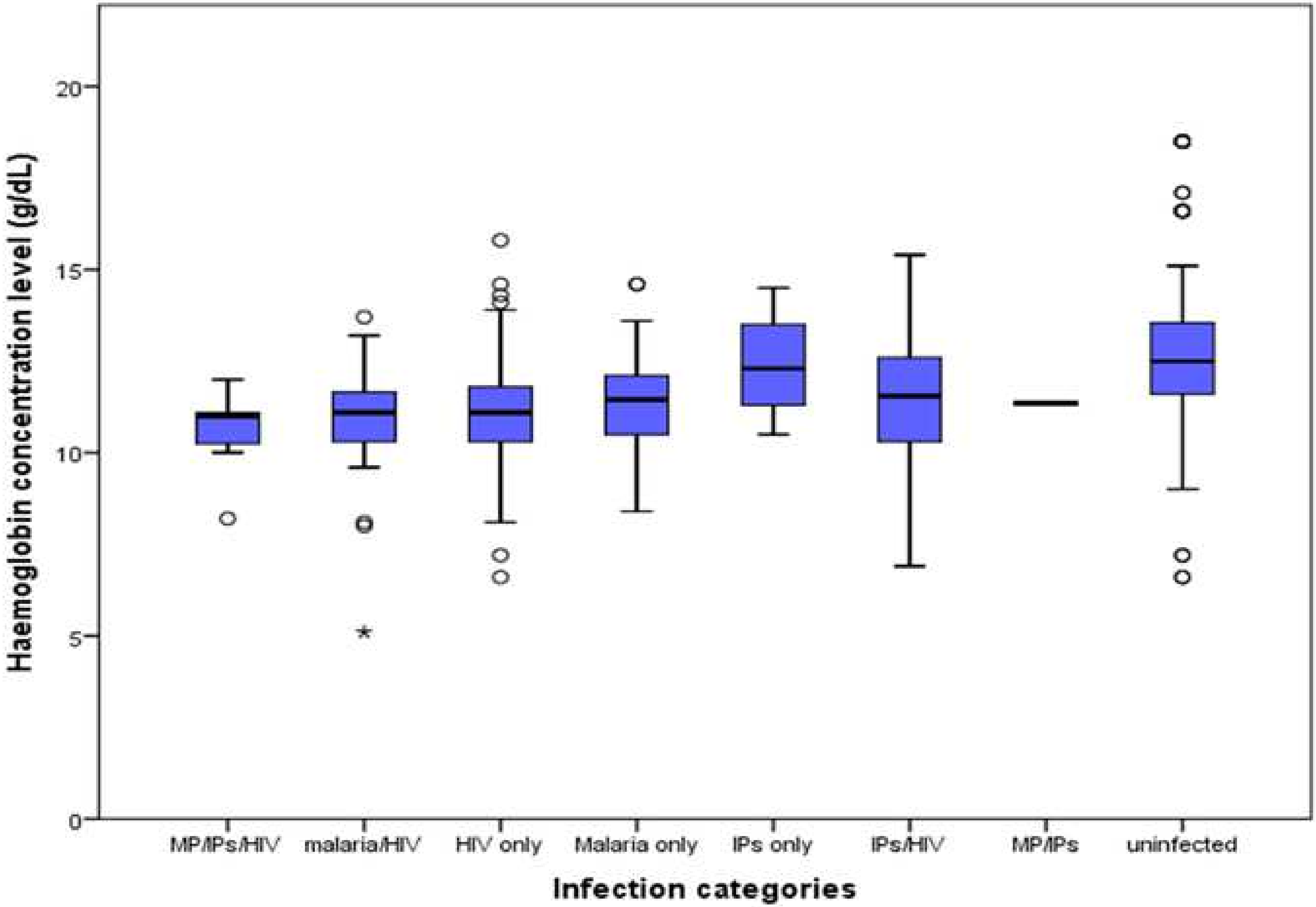

Supplement: Attachment — Submitted filename: For Review PONE-D-20-20708_reviewer.pdf [file pone.0245743.s002.pdf]
