# Supplementary material for: Malaria and intestinal parasite co-infection and its association with anaemia among people living with HIV in Buea, Southwest Cameroon: A community-based retrospective cohort study
Source: PLoS One. 2021 Jan 22;16(1):e0245743. doi: 10.1371/journal.pone.0245743 (PMC7822292; doi:10.1371/journal.pone.0245743)
Supplement: Attachment — Submitted filename: sept26 PONE-D-20-20708_R1.pdf [file pone.0245743.s004.pdf]

PLOS ONE

Malaria and intestinal parasite co-infection and its association with anaemia among people living with HIV in Buea, Southwest Cameroon: A community-based cross-sectional study

--Manuscript Draft--

**Manuscript Number:**	PONE-D-20-20708R1
**Article Type:**	Research Article
**Full Title:**	Malaria and intestinal parasite co-infection and its association with anaemia among people living with HIV in Buea, Southwest Cameroon: A community-based cross-sectional study
**Short Title:**	Malaria and Intestinal parasite co-infection and its association with anaemia in HIV patients.
**Corresponding Author:**	Sorelle Mekachie Sandie, MSc<br>University of Buea<br>Buea, CAMEROON
**Keywords:**	Malaria; Anaemia; Intestinal parasite; HIV, co-infection
**Abstract:**	Background: Both malaria and intestinal parasites are endemic in Cameroon, and their co-infection can be of great impact on anaemia among people living with HIV (PLWH). This community-based cross-sectional study determined the prevalence and impact of infections on anaemia in PLWH and HIV-negative individuals in Buea, Cameroon from March to August 2019.<br>Methods: The study population comprised of 190 PLWH and 216 consenting HIV negative individuals from the Buea community. Participants were examined clinically, the collected blood sample was used for malaria parasite (MP) detection and speciation, HIV diagnosis and haemoglobin (Hb) measurement while stool samples were examined for the detection of intestinal parasites (IPs). Proportions were compared using Pearson's Chi-square test and association of anaemia with independent variables was evaluated using logistic regression analysis.<br>Results: Out of the 406 participants, MP, IPs and MP/IP co-infection prevalences were 15.5%, 13.0% and 3.0% respectively. When compared with HIV negative participants, PLWH had a higher prevalence of MP (16.3%, P = 0.17), IPs ([illegible]%, P < 0.001) and MP/IPs co-infection (3.7%, P = 0.04). Participants with triple infections co-infected with HIV, MP and IP had a significantly lower mean haemoglobin value (10.6 ± 1.21 g/dL). When comparing single infections, the prevalence of anaemia was highest among those who had HIV only (79.3%) when compared with those infected with MP only (71.4%) and IPs only (25.0%). HIV positive patients had a significantly (P < 0.001) higher prevalence of mild (56.8%), moderate (18.4) and severe (1.6%) anaemia when compared with HIV negative counterparts. The significant risk factors associated with anaemia included being febrile (P = 0.03), MP-infected only (P = 0.001), HIV-infected only (P < 0.001), having dual (P < 0.001) or triple-infections (P = 0.03).<br>Conclusion: Multiple infections exacerbate the health condition of PLWH. Routine medical check-ups among PLWH are recommended.
**Order of Authors:**	Sorelle Mekachie Sandie, MSc
	Irene Ule Ngole Sumbele
	Helen Mbufong Kuokuo Kimbi
	Martin Mih Tasah
**Response to Reviewers:**	Responses to Editor's Concerns<br>When submitting your revision, we need you to address these additional requirements.<br>1. Please ensure that your manuscript meets PLOS ONE's style requirements, including those for file naming. The PLOS ONE style templates can be found at https://journals.plos.org/plosone/s/file?id=wjVg/PLOSOne_formatting_sample_main_body.pdf and https://journals.plos.org/plosone/s/file?id=ba62/PLOSOne_formatting_sample_title_authors_affiliations.pdf

Powered by Editorial Manager® and ProduXion Manager® from Aries Systems Corporation

The manuscript has been corrected to meet PLOS ONE's style requirements as suggested by the editor.

2. Thank you for indicating in the text of your manuscript that you obtained written informed consent from participants. Please also add this information to your ethics statement in the online submission form.
Information on ethics statement has been included in the online submission form.

3. Please include additional information regarding the survey or questionnaire used in the study and ensure that you have provided sufficient details that others could replicate the analyses. For instance, if you developed a questionnaire as part of this study and it is not under a copyright more restrictive than CC-BY, please include a copy, in both the original language and English, as Supporting Information.
The questionnaire for this study was developed by the principal investigator. A copy has been uploaded as Supporting Information in the online submission.

Corrections and Concerns of Reviewer #1
The corrections proposed by reviewer #1 have been effected and are all highlighted in yellow in the revised manuscript.

Reviewer #1: The manuscript is well written but the authors need to provide clear description on laboratory methods. The results are well presented but table three need rearrangement to be clear to the reader. The conclusions are based on the study findings
Thanks for the comments. Laboratory methods, Table 3 in the results section as well as the conclusions as suggested by the reviewer have been upgraded and reorganized.

Abstract
Results
Line 37: But this is not statistically significant
Effectively, the difference was not statistically significant across the two groups; but as malaria parasite prevalence was one of the main outcomes in the study, it could not be left out in the abstract because of that. Apart from statistical significance, it is also important to know the trend of the various infections across the groups.
Methods
Study design and sample size
Line 138: This sample size was not intended/powered to compare two proportions
You raised a pertinent issue concerning sample size calculation. To address that, the following sample size formula was used as we are comparing two proportions (See lines 138-143).
N=2(Zα + Zβ)2 P(1-P)/(P0-P1)2
Where;
N=sample size
Zα= 1.96
Zβ= 0.84
P = Pooled prevalence = (P0 + P1)/2
P0 = 50% (unknown prevalence)
P1 = 67.2% (Chanyalew and Gurara, 2014)
The calculated sample size (N) was 128 for each group.
Sample collection and processing
Line 164: Briefly describe how you quantified the parasites
A statement on quantification of parasites has been included in the revised manuscript (See lines 164 – 166).
Line 167: Provide details how quantification was done and quality assurance of the readings
A statement on malaria parasite quantification has been included in the revised manuscript (See lines 166 – 172).
The authors omitted to mention that blood smears were prepared and read by 2 qualified microscopists, and that a third microscopist was requested in case of discrepancy in some results; although that was effectively done. This information has been included under sample collection and processing (See lines 166 -169).
For quality assurance, a negative result was declared after checking at least 200 high power microscopic fields.

Powered by Editorial Manager® and ProduXion Manager® from Aries Systems Corporation

Line 185: Please provide reference
On line 185, the authors just stated the apparatus used for haemoglobin measurement; giving details on the location of the manufacturing company. The authors do not understand which reference should be added on that statement.
Table 3
Line 276: It will be easier to interpret this table if rearranged into two categories HIV+ve and HIV –ve like table2, then see how anemia and co-infection vary across groups.
In Table 3, the authors wanted to bring out clearly the difference in the prevalence of anaemia and its severity with respect to the various categories of infections (single, double and triple infections); so if arranged into two categories, that difference will not be clearly seen.
Figure 1: Please indicate that these participants were excluded/not enrolled.
A statement on that has been included under the description of socio-demographic data and referred to Figure 1 (See lines 216 – 218).

Corrections and Concerns of Reviewer #2
The corrections proposed by reviewer #2 (Rodolfo Rossi) have been effected and are all highlighted in turquoise in the revised manuscript.
Reviewer #2: Thank you for giving me the opportunity to review this manuscript, which is rather well written and clear. The manuscript adds on the evidence of various conditions common is sub-Saharan Africa and links them to the HIV pandemic. However, in spite of its merits, the manuscript should undergo some review prior potential publication.
Both major and minor comments are explained below in order of the page of the manuscript:

Page 3 – Lines 52-55: the authors start the manuscript by trying to give evidence of what is known on the issue, which is good. However, this part could be expanded; there is literature that the authors can consider to make their statements stronger (as an example: https://doi.org/10.1016/j.gore.2019.07.002). Therefore, I suggest an additional search in the literature.
Thank you for the suggestion, which has been taken into consideration by the authors. After literature search, the background has been expanded to make the statements stronger (See lines 54 – 62).

Page 6 – Lines 126-127: It is not clear how the participants were selected, specifically in terms of their HIV status. Were 2 different selections done on the basis of HIV status, meaning, were the authors specifically going to look for HIV+ and HIV- participants? If so, this study should be redesigned as a (retrospective) cohort since participants are selected on the base of an exposure (i.e. HIV) and see the differences in outcomes (i.e. anaemia, IP, etc.). In cross-sectional studies, a sample of the population is selected, not necessarily on the base of some individual variables. Please, clarify this point and rectify it all along the manuscript.
The authors believe this study is a cross-sectional study because the participants were recruited from the communities not based on their exposure as well as from the HIV treatment centre in the community to make up the numbers of HIV positive cases. Hence, we had both exposed (HIV patients who already knew their status) and unexposed participants (HIV negatives who had no knowledge of their status and were tested to confirm their HIV status) recruited from both the community and HIV treatment centre. Those who were tested positive for HIV in the community outreach were included in the HIV positive group.

Page 9 – Lines 194-196: the authors stated that they conducted a binary logistic regression, which appear somehow in contradiction with what written on line 281 ("A multinomial regression analysis was used…"). In logistic regression, usually we talk about "crude" (or univariate) analysis if there is only one exposure and the outcome in the model, or "adjusted" (or multivariable) analysis if there are more than one exposure in the same model. Please, specify which one you used, and if it is crude, please explain (with good reason) why an adjusted analysis was not conducted (which should be). In addition, please, describe which statistical test was used in the logistic regression (which can either be the Wald test or the Likelihood Ratio test).
The authors noticed the contradiction raised by the reviewer concerning the analysis that was used to determine odds ratio and it has been addressed in the revised

Powered by Editorial Manager® and ProduXion Manager® from Aries Systems Corporation

	manuscript.<br>To address the concern regarding odd ratios, the data was reanalyzed and both the crude and adjusted odds ratios were included in table 4 in the revised manuscript. The statistical test used was the Likelihood Ratio test.<br><br>Page 10-11 (Table 1) and page 12-13 (Table 2): you provide descriptive statistics for various variables of participants. Please, provide the p-value from the Chi2 test to see if there are statistically significant differences in the various variables between HIV+ and HIV- participants.<br>As seen in Tables 1 and 2, the P- and Chi-square values have been included to show whether there were statistical significance differences in the various variables between HIV positive and negative participants.<br><br>Page 16 – Table 4: Please, again explain whether the OR are crude or adjusted (see point above); please, include (for clarity) what is the reference for each variable (e.g. if males have a OR of 0.37 for anaemia, it is in comparison to women, which will be the reference) in the table, rather than in the table legenda; please, also include which statistical test was used (see above).<br>The reference for each variable has been included in table 4 for better understanding of the results (See line 308).<br><br>Page 17 onwards (all Discussion section): please reorganize the whole Discussion/Conclusion section. This should be according to a logical sequence. Start by summarizing the findings of your study (no need to repeat values such as percentages and means in the discussion section), how they compare with other similar studies, what are the reasons for differences with other studies. Provide a plausibility for your findings. Provide Strengths and Limitations of your study (this is essential to see to what extent you can give recommendations following your results). Once you have gone through the strengths and limitations of your study, you should provide which are the implications of your results, these may be clinical, public health/programmatic, advocacy etc. as recommendations plus all what you consider to be done as additional research on the topic.<br>The discussion section has been improved as requested by the reviewer (See lines 423-433).
**Additional Information:**	
**Question**	**Response**
**Financial Disclosure**<br><br>Enter a financial disclosure statement that describes the sources of funding for the work included in this submission. Review the submission guidelines for detailed requirements. View published research articles from *PLOS ONE* for specific examples.<br><br>This statement is required for submission and **will appear in the published article** if the submission is accepted. Please make sure it is accurate.	The author(s) received no specific funding for this work.

**Unfunded studies**<br>Enter: *The author(s) received no specific funding for this work.*<br><br>**Funded studies**<br>Enter a statement with the following details:<br>• Initials of the authors who received each award<br>• Grant numbers awarded to each author<br>• The full name of each funder<br>• URL of each funder website<br>• Did the sponsors or funders play any role in the study design, data collection and analysis, decision to publish, or preparation of the manuscript?<br>• **NO** - Include this sentence at the end of your statement: *The funders had no role in study design, data collection and analysis, decision to publish, or preparation of the manuscript.*<br>• **YES** - Specify the role(s) played.<br><br>* typeset	
**Competing Interests**<br><br>Use the instructions below to enter a competing interest statement for this submission. On behalf of all authors, disclose any competing interests that could be perceived to bias this work—acknowledging all financial support and any other relevant financial or non-financial competing interests.<br><br>This statement **will appear in the published article** if the submission is accepted. Please make sure it is accurate. View published research articles from *PLOS ONE* for specific examples.	The authors have declared that no competing interests exist.

Powered by Editorial Manager® and ProduXion Manager® from Aries Systems Corporation

**NO authors have competing interests**<br><br>Enter: *The authors have declared that no competing interests exist.*<br><br>**Authors with competing interests**<br><br>Enter competing interest details beginning with this statement:<br><br>*I have read the journal's policy and the authors of this manuscript have the following competing interests: [insert competing interests here]*<br><br>* typeset	
**Ethics Statement**<br><br>Enter an ethics statement for this submission. This statement is required if the study involved:<br><br>• Human participants<br>• Human specimens or tissue<br>• Vertebrate animals or cephalopods<br>• Vertebrate embryos or tissues<br>• Field research<br><br>Write "N/A" if the submission does not require an ethics statement.<br><br>General guidance is provided below. Consult the submission guidelines for detailed instructions. **Make sure that all information entered here is included in the Methods section of the manuscript.**	Using an information sheet, a brief talk was given to the participants in the English Language (and exceptionally in Pidgin English) and the participants were then invited to participate in the study by signing an informed consent form. In the case of children (≤ 15years), their parents signed proxy informed consent form on their behalf. Ethical clearance was obtained from the Institutional Review Board hosted by the Faculty of Health Sciences of the University of Buea (Reference Number: 661-09). For confidentiality purposes, patients were identified by special identification numbers. Results of participants with parasitic infections in the neighborhood were referred to a clinician for their case management.

Powered by Editorial Manager® and ProduXion Manager® from Aries Systems Corporation

**Format for specific study types**<br><br>**Human Subject Research (involving human participants and/or tissue)**<br>• Give the name of the institutional review board or ethics committee that approved the study<br>• Include the approval number and/or a statement indicating approval of this research<br>• Indicate the form of consent obtained (written/oral) or the reason that consent was not obtained (e.g. the data were analyzed anonymously)<br><br>**Animal Research (involving vertebrate animals, embryos or tissues)**<br>• Provide the name of the Institutional Animal Care and Use Committee (IACUC) or other relevant ethics board that reviewed the study protocol, and indicate whether they approved this research or granted a formal waiver of ethical approval<br>• Include an approval number if one was obtained<br>• If the study involved *non-human primates*, add *additional details* about animal welfare and steps taken to ameliorate suffering<br>• If anesthesia, euthanasia, or any kind of animal sacrifice is part of the study, include briefly which substances and/or methods were applied<br><br>**Field Research**<br><br>Include the following details if this study involves the collection of plant, animal, or other materials from a natural setting:<br>• Field permit number<br>• Name of the institution or relevant body that granted permission	
**Data Availability**<br><br>Authors are required to make all data underlying the findings described fully available, without restriction, and from the time of publication. PLOS allows rare exceptions to address legal and ethical concerns. See the PLOS Data Policy and FAQ for detailed information.	No - some restrictions will apply

A Data Availability Statement describing where the data can be found is required at submission. Your answers to this question constitute the Data Availability Statement and **will be published in the article**, if accepted.<br><br>**Important:** Stating 'data available on request from the author' is not sufficient. If your data are only available upon request, select 'No' for the first question and explain your exceptional situation in the text box.<br><br>Do the authors confirm that all data underlying the findings described in their manuscript are fully available without restriction?	
**Describe where the data may be found in full sentences. If you are copying our sample text, replace any instances of XXX with the appropriate details.**<br><br>• If the data are **held or will be held in a public repository**, include URLs, accession numbers or DOIs. If this information will only be available after acceptance, indicate this by ticking the box below. For example: *All XXX files are available from the XXX database (accession number(s) XXX, XXX.*).<br>• If the data are all contained **within the manuscript and/or Supporting Information files**, enter the following: *All relevant data are within the manuscript and its Supporting Information files.*<br>• If neither of these applies but you are able to provide **details of access elsewhere**, with or without limitations, please do so. For example:<br><br>*Data cannot be shared publicly because of [XXX]. Data are available from the XXX Institutional Data Access / Ethics Committee (contact via XXX) for researchers who meet the criteria for access to confidential data.*<br><br>*The data underlying the results presented in the study are available from (include the name of the third party*	All relevant data are within the manuscript

and contact information or URL).<br>• This text is appropriate if the data are owned by a third party and authors do not have permission to share the data.<br><br>* typeset	
Additional data availability information:	

Manuscript Click here to access/download;Manuscript;Manuscript.docx

Malaria and intestinal parasite co-infection and its association with anaemia among people living with HIV in Buea, Southwest Cameroon: A community-based cross-sectional study

Sorelle Mekachie Sandie[1*¶], Irene Ule Ngole Sumbele[1, 2¶], Martin Mih Tasah[1] and Helen Kuokuo Kimbi[1,3¶]

1 Department of Zoology and Animal Physiology, University of Buea, Buea, Cameroon.

2 Department of Microbiology and Immunology, Cornell College of Veterinary Medicine, Ithaca, New York, USA.

3 Department of Medical Laboratory Science, The University of Bamenda, Bambili, Cameroon

*Corresponding author

E- mail: **sandiesorelle2@gmail.com** **(SMS)**

¶These authors contributed equally to this work.

Abstract

Background: Both malaria and intestinal parasites are endemic in Cameroon, and their co-infection can be of great impact on anaemia among people living with HIV (PLWH). This community-based cross-sectional study determined the prevalence and impact of infections on anaemia in PLWH and HIV-negative individuals in Buea, Cameroon from March to August 2019.

Methods: The study population comprised of 190 PLWH and 216 consenting HIV negative individuals from the Buea community. Participants were examined clinically, the collected blood sample was used for malaria parasite (MP) detection and speciation, HIV diagnosis and haemoglobin (Hb) measurement while stool samples were examined for the detection of intestinal parasites (IPs). Proportions were compared using Pearson's Chi-square test and association of anaemia with independent variables was evaluated using logistic regression analysis.

Results: Out of the 406 participants, MP, IPs and MP/IP co-infection prevalences were 15.5%, 13.0% and 3.0% respectively. When compared with HIV negative participants, PLWH had a higher prevalence of MP (16.3%, P = 0.17), IPs (23.7%, P < 0.001) and MP/IPs co-infection (3.7%, P = 0.04). Participants with triple infections co-infected with HIV, MP and IP had a significantly lower mean haemoglobin value (10.6 ± 1.21 g/dL). When comparing single infections, the prevalence of anaemia was highest among those who had HIV only (79.3%) when compared with those infected with MP only (71.4%) and IPs only (25.0%). HIV positive patients had a significantly (P < 0.001) higher prevalence of mild (56.8%), moderate (18.4) and severe (1.6%) anaemia when compared with HIV negative counterparts. The significant risk factors associated with anaemia included being febrile (P = 0.03), MP-infected only (P = 0.001), HIV-infected only (P < 0.001), having dual (P < 0.001) or triple-infections (P = 0.03).

Conclusion: Multiple infections exacerbate the health condition of PLWH. Routine medical check-ups among PLWH are recommended.

Background

Parasitic infections are a major cause of morbidity and mortality in Africa; especially in resource-limited tropical and sub-tropical regions like sub-Saharan Africa [1] where the majority of HIV/AIDS cases are concentrated. In 2018, there were roughly 1.7 million new HIV infections, which is an insignificant improvement when compared to 2017 (1.8 million) [2]. The same year, Cameroon reported an HIV incidence of 1.02 and a national prevalence of 3.6% [2]. People living with HIV (PLWH) are threatened by a great number of diseases caused by different types of biological agents. Although the morbidity and mortality due to malaria and intestinal parasites can be controlled by the application of various prevention measures put in place by the Cameroon government as well as the delivery of chemotherapy, elimination and final eradication will not be achieved until populations live in mosquito-free environments and have access to effective sanitation, sewage treatment and waste disposal; which remain a trending problem in Cameroon.

HIV infection is characterized by immunosuppression that exposes the infected individual to a variety of microbial and parasitic infections such as *Plasmodium*, *Mycobacterium tuberculosis* and intestinal parasitic infections [3]. These infections (opportunistic or not) are a serious threat to the health status of people living with HIV, especially in developing countries like Cameroon. In an attempt to achieve HIV epidemic control in 2021, the Cameroon Government has been implementing various strategies that led to an encouraging increase in antiretroviral therapy (ART) coverage (53%) and viral load (VL) suppression (44.7%) nationally in 2019 [4].

African countries still bear the largest burden of malaria morbidity (93%), with *Plasmodium falciparum* accounting for 99.7% of malaria cases in 2018 [5]. In 2018, WHO reported that Cameroon featured the fourth country behind Nigeria, Democratic Republic of Congo and Mozambique with the highest malaria prevalence [6]. Many studies have consistently reported an association between HIV infection and malaria in Cameroon [7, 8, 9, 10, 11], with each region showing a varied prevalence of both malaria and HIV infection. Malaria transmission is stable throughout the year, with an overall malaria test positivity rate of 62% registered in 2017 [12].

The burden of intestinal parasite infections (IPIs) is greatest in low-income areas and are major public health problems in certain areas of Sub-Saharan Africa. These IPIs are associated with a humid climate, unsanitary environments and poor socio-economic conditions. Parasites causing these IPIs are protozoans (*Cryptosporidium species, Cyclospora cayetanensis, Isospora belli, Microsporidia spp* and *Entamoeba histolytica*) and helminths (*Ascaris lumbricoides, Trichuris trichuiria,* hookworms, *Schistosoma mansoni* and *Taenia species*). *Cryptosporidium species, Cyclospora cayetanensis*, *Isospora belli*, and *Microsporidia spp* are often referred to as opportunistic parasites in people living with HIV because they cause great morbidity especially when CD4 T cell count falls below 200 cells/μL [13]. Various studies carried out among African adults living with HIV have reported IPI rates ranging from 10% to 45% [14, 15]. Majority of the research on the impact of IPIs on HIV progression in Cameroon, occurred before the wide availability of ART [16] hence, the need for further evaluation.

In Cameroon, PLWH face similar environmental realities as those not infected with HIV and so can easily get co-infected with combinations of intestinal and malaria parasites. Such co-infections may have considerable health consequences which could lead to severe clinical symptoms and pathology in PLWH. Studies have shown that individuals with multiple infections have an increased risk of developing more frequent and severe diseases due to interactions among infecting parasite species [17-19].

Malaria and intestinal parasites in PLWH can both lead to severe haematological abnormalities. Although ART in combination with cotrimoxazole confers a level of immunity to infections in PLWH, it has been reported to have adverse effects on the red blood cell indices of these patients, thereby causing anaemia [20]. Although malaria and intestinal parasites, as well as ART, are known etiological factors in anaemia [11, 16, 21, 22], the extent to which their interaction might enhance the risk of anaemia merits further investigations. Previous studies have reported on the influence of malaria/HIV co-infection on the occurrence of anaemia [11, 19, 23]. Co-infections in HIV are numerous and important causes of morbidity and mortality. Combating co-infections has been identified as an important public health goal. The situation regarding PLWH co-infected with malaria and intestinal parasites in the area following the intensification of different control strategies such as free availability of ART and cotrimoxazole, access to viral load testing and community ART dispensation is unknown. Therefore, the present study was undertaken to determine the prevalence of malaria parasite and intestinal parasites in PLWH compared to HIV negative counterparts as well as to assess the effects of these infections on anaemia to provide relevant information to policymakers in the country.

Methods

Study area and participants

The study was carried out in the HIV Treatment and Care Centre at the Regional Hospital and different neighbourhoods in the Buea community. The study area has been described in detail by Sumbele et al [24]. The Buea Regional Hospital is the referral HIV treatment centre in Fako Division serving the population and hence receives HIV positive patients daily from the various neighbourhoods for their monthly distribution of ART. With the presence of the Mount Cameroon, Buea is a major touristic site and has a business-friendly environment. In the national HIV prevalence ranking, the South West Region is the 6th Region with a prevalence of 3.6% which equates the national prevalence (3.7%) [25] as well as the second to the last Region (33.8%) with the least viral load suppression. Buea is an area of moderate malaria transmission, with a peak time for malaria transmission during and just after the rainy season (March to September) [26]. Its tropical climate provides a suitable environment for the development of the malaria vectors and this therefore, contributes to increased malaria transmission. There is also inadequate access to safe water in this region, predisposing the population to water-borne infections including IPIs.

The two categories of participants, PLWH and HIV-negative individuals were enrolled in the study at the HIV Treatment and Care Centre and from the different neighbourhoods. Only participants who signed the informed consent form and willingly accepted to be counselled and tested for malaria, IPIs and HIV were enrolled in the study. Participants on antibiotics, malaria treatment or antiparasitic agents two weeks before enrolment were excluded from the study.

Study design and sample size

This study was a community-based cross-sectional study carried out between April 2018 and August 2019. HIV-negative participants included persons who consented to be screened for HIV during the process and were negative. A convenience method of sampling was employed and participants were enrolled through HIV outreach programs in their homes and offices in the Buea neighbourhoods (Molyko, Bonduma, Great Soppo, Government reserve area (GRA), Buea town, Bokwaongo, Bokova, Mile 18, Clerks and Federal quarters). The sample size was determined using the formula $N=2(Z_{\alpha} + Z_{\beta})^2 P(1-P)/(P_0-P_1)^2$ [27] where N represented the sample size evaluated, Z_{α} was 1.96 and Z_{β} was 0.84 which are the standard normal deviates (for the 95% confidence interval, CI), P_0 (Previous prevalence of MP/IPs co-infection in PLWH) was 50% and P_1 (Previous prevalence of MP/IPs co-infection in HIV negative individuals) was 67.2% [28]. The sample size calculated was 128 for each group giving an optimum sample size of 256 participants for the study.

Data collection

Questionnaire

A pre-structured questionnaire was used to collect socio-demographic information such as age, sex, location of residence, level of education, marital status and occupation while clinical data such as ART usage, duration on treatment, ART regimen and viral load (VL) were collected from each HIV patient's file at the HIV Treatment and Care Centre. Health and preventive practices towards malaria and IPIs as well as behavioural habits regarding health conditions with a history of symptoms were recorded.

Sample collection and processing

Participants were instructed on how to collect stool sample in universal transparent faecal containers with a screw cap lid. After the provision of a stool sample, the participant's body temperature was measured using a digital thermometer and fever was defined as temperature ≥ 37.5ºC. Blood was collected under sterile conditions into a well-labelled Ethylenediaminetetraacetate (EDTA) tube for each participant for HIV screening and confirmation, malaria diagnosis and haemoglobin (Hb) measurement. The collected samples were transported to the Malaria Research Laboratory of the University of Buea for further analysis.

On arrival in the laboratory, the stool samples were analysed macroscopically and preserved in 10% formalin. Formol-ether concentration technique and Modified Ziehl-Neelsen (ZN) methods [29] were performed for the detection of intestinal parasites (protozoans and helminths). To estimate parasite intensity, egg counts per slide were converted to egg per gram of faeces (epg) by multiplying the number of eggs on the slide by 24. The blood sample was used to prepare thick and thin blood films on a labelled slide that was Giemsa-stained, analysed following standard procedures and the parasite density per microliter of blood was calculated based on the number of parasites per 200 leucocytes on the thick blood film, assuming a leucocyte count of 8000 leucocytes/µL of blood as described by Cheesbrough [29]. All blood and stool slides were examined by two microscopists who were blinded to the results, and where positive/negative discrepancy occurred, slides were counter-read by a third microscopist, who was blinded regarding the two previous results. A negative result was declared after checking at least 200 microscope fields. The blood was also used for HIV rapid test (in the case of presumed HIV-negative participants) and Hb measurement. Viral loads were obtained from the HIV positive participant's file at the HIV Care Centre. Viral load < 50 copies /mL of blood was considered undetectable [30].

CD4T cell counts were determined using the BD FACS Count™ System (BD Biosciences, USA) as per the manufacturer's instructions. To assure the quality of the laboratory data, standard operating procedure for each test was followed. Reagents were checked for expiry date and prepared according to the manufacturer's instruction. The Determine Rapid Test Kit (Abott Laboratories, CO., Ltd. Minato-Ku, Tokyo Japan) was used to test the supposed HIV negative subjects for HIV-1 following the manufacturer's instructions.

Haemoglobin (Hb) measurement

The full blood count (FBC) analysis was carried out using the URIT-3300 automated haematology analyser (URIT Medical Electronic CO., LTD. Jiuhua Road, Gungxi, China), following the manufacturer's instructions. Haemoglobin concentration was obtained from the FBC results. Anaemia was defined as Hb concentrations below the World Health Organization (WHO) reference values corresponding to age or gender [31]. Further classification was done to determine severe (Hb < 7g/dL), moderate (Hb between 7.0 and 10.0 g/dL) and mild anaemia (> 10 g/dL Hb < 11g/dL) cases [29].

Statistical Analysis

Data were analysed using IBM-Statistical Package for the Social Science (SPSS) version 20.0 software (SPSS Inc., Illinois, USA). Baseline characteristics were summarized as mean with 95% CI and compared using the independent *t*-test. Malaria and intestinal parasites data were summarized into means and standard deviation (SD) and percentages were used in the evaluation of the descriptive statistics. The significance of differences in prevalence was explored using Pearson's Chi-square while Analysis of Variance (ANOVA) and Student's t-test was used where

appropriate to assess difference in group means. The association of anaemia with the independent variables was evaluated using logistic regression model. $P < 0.05$ was considered for statistical significance.

Ethical considerations

Using an information sheet, a brief talk was given to the participants in the English Language (and exceptionally in Pidgin English) and the participants were then invited to participate in the study by signing an informed consent form. In the case of children (≤ 15years), their parents signed proxy informed consent form on their behalf. Ethical clearance was obtained from the Institutional Review Board hosted by the Faculty of Health Sciences of the University of Buea (Reference Number: 661-09). For confidentiality purposes, patients were identified by special identification numbers. Results of participants with parasitic infections in the neighbourhoods were referred to a clinician for their case management.

Results

Socio-demographic and clinical characteristics of the participants

Five hundred and eighty-nine (589) participants were approached to participate in the study, those who could not provide stool sample and whose blood sample could not be collected were excluded from the study; giving a total of 406 (68.9%) successfully enrolled participants. Out of the 406 participants, 190 were HIV positive patients from the HIV Treatment and Care Centre and 216 were HIV-negative from the communities as shown in Fig 1.

Fig 1: Study flow diagram showing compliance of the participants

Overall, the mean ± SD age of the participants was 31.2 ± 16.7 (range = 1–70) years. There were more females (68.5%) than males (31.5%). A greater proportion of the study population belonged to the > 40 years age group (31.0%) and the least to the 31–40 years age group (18.7%). Majority of the PLWH had primary level of education (66.7%), were self-employed (31.1%) and were single (62.0%). Among HIV negative participants, majority had university level of education (35.2%), were students (56.5%) and single (63.0%) as revealed in Table 1. Less than half of the study population (45.6%) used ITNs as a preventive measure against malaria. A greater proportion of PLWH used ITNs (57.4%) more than the HIV negative counterparts (35.2%). As seen in Table 1, there were statistically significant differences in the various variables between HIV+ and HIV- participants apart from marital status.

Table 1. Characteristics of the study population

**Parameters**		**HIV Positive**	**HIV Negative**	**Overall**	χ^2
		**n (%)**	**n (%)**	**n (%)**	**P-value**
**Age groups (years)**	≤ 20	42 (22.1)	72 (33.3)	114 (28.1)	48.64
	21-30	20 (10.5)	70 (32.4)	90 (22.2)	< 0.001*
	31-40	48 (25.3)	28 (13.0)	76 (18.7)	
	> 40	80 (42.1)	46 (21.3)	126 (31.0)	
**Mean age ± SD (years)**		35.5±16.5	27.6±15.8	32.3±16.7	
**Sex**	Female	140 (73.7)	138 (63.9)	278 (68.5)	4.49
	Male	50 (26.3)	78 (36.1)	128 (31.5)	0.02*
	No formal	6 (3.7)	18 (8.6)	24 (6.0)	

**Educational Level**	Primary	126 (66.3)	54 (25.7)	180 (45.1)	
	Secondary	43 (22.6)	64 (30.5)	107 (26.8)	78.94
	Tertiary	14 (7.4)	74 (35.2)	88 (22.1)	< 0.001*
**Occupation**	Student	48 (25.4)	122 (56.5)	171 (42.1)	
	Unemployed	25 (13.2)	12 (5.2)	37 (9.1)	45.38
	Self-employed	59 (31.2)	28 (13.0)	87 (21.4)	< 0.001*
	Employee	57 (30.2)	54 (25.0)	111 (27.3)	
**Marital status**	Single	93 (62.0)	102 (63.0)	197(62.5	0.03
	Married	61 (38.0)	60 (37.0)	117(37.5)	0.47
**ITN usage**	Yes	109 (57.4)	76 (35.2)	185 (45.6)	20.05
	No	81 (42.6)	140 (64.8)	221 (54.4)	< 0.001*

Axillary temperature measurement revealed that 14.0% (57) of the general population were feverish, with the highest proportion (15.7%) among HIV negative participants. Malaria clinical signs (headache, joint pains, nausea, vomiting etc…) were less frequent among HIV negative participants than among PLWH (23.4%). Anaemia occurred in 57.1% (232) of the participants and PLWH had the highest occurrence (76.8%) when compared with their HIV negative counterparts and the difference was statistically significant ($\chi^2 = 56.59$, $P < 0.001$). Among PLWH, half of the population (50.0%) had CD4 T cell count between 200-500 cells/µL of blood, the majority (97.9%) were on ART, 91.9% (171) were taking zidovudine-based ART and 77.5% (141) had attained viral suppression(< 50 copies/mL of blood) as shown in Table 2.

Table 2. Clinical characteristics and laboratory profile of the study population

Parameters		HIV Positive n (%)	HIV Negative n (%)	Overall n (%)	χ^2 P-value
**Fever status**	Febrile	23 (12.1)	34 (15.7)	57 (14.0)	1.12
	Afebrile	167 (87.9)	182 (84.3)	349 (86.0)	0.18
**Mean temperature ±SD (ºC)**		37.1 ±0.5	37.1 ±0.5	37.1 ±0.5	
**Malaria clinical signs**	Yes	39 (23.5)	32 (18.2)	71 (20.7)	1.40
	No	127 (76.5)	144 (81.8)	272 (79.3)	0.15
**Anaemic status**	Anaemic	146 (76.8)	86 (39.8)	232 (57.1)	56.59
	Non-anaemic	44 (23.2)	130 (60.2)	174(42.9)	< 0.001*
**Mean Hb ±SD** (g/dL)		11.1±1.5	12.5 ±2.1	11.8 ±1.9	
**CD4 T-cell count(cells/µL)**	< 200	15 (14.2)	-	15 (14.2)	-
	200 – 500	51 (48.1)	-	51 (48.1)	
	>500	40 (37.7)	-	40 (37.7)	
**Mean CD4 T cell count ±SD (cells/µL)**		466.0 ±275.9	-	466.0±275.9	
**ART usage**	Yes	186 (97.9)	-	186 (97.9)	-
	No	4 (2.1)	-	4 (2.1)	
**Zidovudine-based ART**	Yes	171 (91.9)	-	171 (91.9)	-
	No	15 (8.1)	-	15 (8.1)	
**ART Duration (years)**	0	4 (2.1)		4 (2.1)	-
	< 1	36 (19.4)	-	36 (19.4)	
	1 -3	61 (32.8)	-	61 (32.8)	
	>3	89 (47.8)	-	89 (47.8)	
**Viral load (RNA copies/ml)**	Not detectable (<50)	141 (77.5)	-	141 (77.5)	-
	≥ 50	41 (22.5)	-	41 (22.5)	

Malaria parasite, intestinal parasite infection and MP/IPI co-infection prevalence

Out of the 406 participants, 63 (15.5%) were positive for MP with *Plasmodium falciparum* as the only species observed. MP prevalence was higher in HIV positive pa[illegible]ipants (16.3%) than those negative (14.8%) although not statistically significant (P = 0.39). Overall, 1[illegible]% (65) of the participants were positive for IPIs with a higher occurrence of protozoa (10.4%) than helminths (3.8%). The most prevalent protozoa parasite was *Cryptosporidium* species while that of helminths was hookworm. The prevalence of IPs was significantly higher ($\chi^2 = 31.73$, $P < 0.001$) among PLWH (23.7%) than in those negative (5.6%). Malaria/IPs co-infection prevalence was 2.7% (11) and was comparable (P = 0.20) among PLWH (3.7%) and HIV negative particip[illegible]s (1.9%) as shown in Fig. 2.

Fig 2: Prevalence of MP, IPIs and MP/IPIs co-infection in the study population

Infection categories, haemoglobin level and anaemia prevalence

Out of the 406 participants, 1.7% (7) had triple infection (MP, HIV and IPIs), 9.4% (38), 5.9% (24) and 1.0% (4) were co-infected with IPIs/HIV, MP/HIV and MP/IPIs respectively. Equally, 29.8% (121) had HIV only, 6.9% (28) had malaria only and 2.0 (8) had IPIs only. The rest of the participants (43.3%, 176) were uninfected. As shown in Fig 3, there was a significant difference ($H = 68.24$, $P < 0.001$) when comparing mean haemoglobin values between the different infection categories; as those triple infected had the lowest mean haemoglobin value (10.55 ± 1.20g/dL) when compared with dual and single infected patients accordingly.

Fig. 3: Box and whisker plots showing mean haemoglobin levels by infection categories

The prevalence of anaemia in the study population was 57.1% (232) and PLWH had a significantly higher (χ^2= 56.59, P < 0.001) prevalence of anaemia (76.8%) than their HIV negative counterparts (39.8%). When comparing single infections, the prevalence of anaemia was highest among those who had HIV only (79.3%), followed by those infected with malaria parasite only (71.4%) and then those with IPs only (25.0%) and the difference was statistically significant (P = 0.002). Participants infected with MP, HIV and IPs simultaneously were observed to have the highest prevalence of anaemia (85.7%) when compared with other co-infection categories as shown in Table 3.

With respect to anaemia severity, HIV positive patients had a significantly (P < 0.001) higher prevalence of mild (56.8%), moderate (18.4) and severe (1.6%) anaemia when compared with HIV negatives. Severity of anaemia with respect to infection categories is seen in Table 3.

Table 3. Prevalence of anaemia, anaemia severity and mean (SD) Hb levels by infection category

Infection category	Number examined N	Prevalence of anaemia n (%)	Mean (SD) Hb (g/dL)	Prevalence of anaemia severity		
				Mild anaemia n (%)	Moderate anaemia n (%)	Severe anaemia n (%)
**HIV positive**	190	146 (76.8)	11.11 (1.54)	108 (56.8)	35 (18.5)	3 (1.6)
**HIV negative**	216	86 (39.8)	12.45 (2.06)	72 (33.3)	12 (5.6)	2 (0.9)
**Test statistic**	χ^2= 56.59, P < 0.001*		U = 29316 P < 0.001*	χ^2= 58.74, P < 0.001*		
**HIV only**	121	96 (79.3)	11.06 (1.44)	72 (59.5)	23 (19.0)	1 (0.8)
**MP only**	28	20 (71.4)	11.57 (1.52)	18 (64.3)	2 (7.1)	0 (0.0)

**IPs only**	8	2 (25.0)	12.40 (1.52)	2 (25.0)	0 (0.0)	0 (0.0)
**Test statistic**	χ^2= 12.12, P = 0.002*		H = 6.87 P < 0.001*	χ^2= 14.47, P = 0.02*		
**MP/HIV**	24	19 (79.2)	10.83 (1.81)	15 (62.5)	3 (12.5)	1 (4.2)
**HIV/IPs**	38	25 (65.8)	11.49 (1.68)	17 (44.7)	7 (18.4)	1 (2.6)
**MP/IPs**	4	2 (50.0)	11.35 (0.06)	2 (50.0)	0 (0.0)	0 (0.0)
**MP/HIV/IPs**	7	6 (85.7)	10.55 (1.20)	4 (57.1)	2 (28.6)	0 (0.0)
**Test statistic**	χ^2= 2.88, P = 0.83		H = 3.84 P = 0.28	χ^2= 5.07, P = 0.82		

H: Kruskal Wallis test; U Mann Whitney U test.

Risk factors of anaemia

A logistic regression model was used to determine the factors associated with anaemia prevalence. The model revealed sex (P < 0.001), age group (P = 0.003), fever status (P = 0.035), infection with HIV only (P = 0.024), infection with MP only (P = 0.004) as well as HIV/MP co-infection (P = 0.027) as significant predictors of anaemia in the study population (Table 4). Male participants and those aged 21–30 years of age were respectively 3 and 2 times more likely to be anaemic than their respective counterparts.

Table 4: Logistic regression model examining factors that influence the prevalence of anaemia in the study population

Variables	Bivariate		Multivariate	
	**COR**	**P-value**	**AOR**	**P value**
**Age group (years)**				
**≤ 20**	1.18	0.521	-	-
**21 – 30**	1.79	0.032*	-	-

**31 – 40**	0.92	0.771	-	-
**> 40**	Reference	-	-	-
**Sex**				
**Male**	2.68	< 0.001*	0.37	< 0.001*
**Female**	Reference	-	reference	-
**Febrile status**				
**Febrile**	0.52	0.035*	1.92	0.010*
**Non-febrile**	Reference	-	reference	-
**Single infection**				
**HIV only**	0.13	0.024*	0.18	0.020*
**MP only**	0.08	0.004*	0.10	0.003*
**IP only**	Reference	-	reference	-
**Dual infection**				
**HIV/MP**	0.31	0.027*	-	-
**HIV/IP**	0.63	0.192	-	
**MP/IP**	1.21	0.851	-	-
**None**	Reference	-	-	-
**Triple infection (HIV/MP/IP)**				
**Yes**	0.30	0.282	-	-
**No**	Reference	-	-	-
**Suppressed viral load**				
**Yes**	1.26	0.600	-	-
**No**	Reference	-	-	-
**Zidovudine-based ART**				
**Yes**	0.78	0.695	-	-
**No**	Reference	-	-	-
**CD4 T-cell count (cells/μL)**				
**< 200**	0.35	0.212	-	-
**200–500**	0.64	0.361	-	-
**> 500**	Reference	-	-	-

*Statistically significant P-value
The test used in the logistic regression was the Likelihood Ratio test.

Discussion

Parasitic infections (especially malaria and intestinal parasites) are major public health concerns among PLWH in developing countries, particularly Sub-Saharan Africa which has been reported to have the highest burden of HIV [2]. This study was a community-based cross-sectional study carried out to determine the prevalence of malaria and intestinal parasites and its association with anaemia among PLWH and HIV negative individuals in Buea, in the South West Region of Cameroon.

In the present study, the overall prevalence of malaria parasite was 15.5%, irrespective of the HIV status of the participants. This result is lower than the recently reported national prevalence (24.4%) [32]. This decrease could be attributed to the restless efforts of the Cameroon Government over the years to implement malaria control strategies to curb down malaria morbidity and mortality on the entire territory; knowing that Cameroon comes fourth on the list of countries that account for the burden of malaria in the WHO African Region [6].

The prevalence of malaria parasite among PLWH was 16.3%, which is high compared to the prevalence reported in PLWH in other areas of the South West Region of Cameroon. This includes the 7.8% reported in Limbe [11] and 2.3% in Buea [33] in 2019 and 2018 respectively. It was also observed in this study that despite the efforts made to scale up insecticide-treated nets (ITNs) distribution so that universal coverage can be attained, coverage remains low (57.4%) and this

could have contributed to this rise in malaria prevalence among PLWH. Another reason for this difference could be the difference in the age distribution of the study population.

The prevalence of malaria parasite was slightly higher in HIV positive patients than in their HIV negative counterparts in line with findings in studies carried out in Nigeria [34]. Interactions between malaria and HIV have been studied extensively, which consider HIV positive patients as a high-risk group for malaria in endemic areas like Cameroon [10, 11, 33, 35]. This slight difference is not significant and this could be as a result of the government actions through the availability of different ACTs and ITNs for both PLWH and HIV-negative Cameroonians; as well as the increasing availability and rapid scale-up of ART and cotrimoxazole to HIV patients in particular. It is worth noting that the HIV negative participants in the communities with malaria parasite are asymptomatic carriers and could greatly fuel the transmission of the malaria parasite in the community. However, the low prevalence shows that strategies implemented by the Cameroon government over the years are effective as this prevalence is lower than the reported national prevalence of malaria (29%) in 2018 [6].

The febrile status of most of the participants in the study irrespective of their HIV status was attributed to the presence of malaria parasite. This is in line with other studies carried out in Cameroon [11] and in Gabon [36]. Also, feverish PLWH had the highest prevalence of MP/IPs co-infection asserting the fact that fever remains an important feature in the clinical diagnosis of infections. Fever is one of the most common clinical signs and characteristic feature of both parasitic and microbial infections.

The prevalence of IPs in Cameroon varied from 27.8% in 2012 [37], to 14.6% in 2013 [38] and 13.0% in this present study. Considering the HIV status, PLWH had a higher prevalence of IPs when compared with their HIV negative counterparts. This prevalence among PLWH is lower than the 59.52% [38] in 2013 and 57.48% [39] in 2014 reported among HIV patients in the Centre and West Regions of Cameroon respectively. This observed decrease in the prevalence of IPs among HIV patients may be accredited to the attainment of viral suppression by a majority (77.5%) as well as their better awareness of IPs and their causes. It may also be due to the improvement in the care provided to PLWH by the government of Cameroon by ensuring a constant supply and provision of ART free of charge.

This study revealed 3.7% of PLWH were co-infected with MP and IPs. Limited information is available on co-infection among PLWH in Cameroon; but when compared with other studies carried out among children [19, 40, 41], the prevalence is low. The mean age of PLWH, as well as the undetectable viral load observed in the majority of these patients, could account for this low prevalence. The lower the viral load, the faster the patient's immune system will recover, increasing the chances of fighting any infection easily. Besides, the majority of these patients have been on ART regularly for more than a year which probably could have contributed in boosting their immune systems against these parasites.

PLWH had a higher prevalence of anaemia when compared with their HIV negative counterparts. This prevalence is higher than the less than 56.0% reported in several studies carried out among PLWH [10, 11, 42-46,]. The reason for the observed difference might be due to the heterogeneity of the study population as the previously reported anaemia prevalence were those of children or

adult HIV patients, while this study included both children and adult participants (1–70 years old). Malaria and HIV positivity, as well as fever, were identified in this study as risk factors of anaemia and therefore contributed significantly to the high prevalence of anaemia. Mixed nutritional deficiencies (iron, folic acid, or vitamin B_{12}), though not investigated in this study, could have contributed to this high prevalence of anaemia as reported by a study carried out by Volberding et al. [47]. A direct comparison of the prevalence of anaemia in different studies is difficult as the study population, inclusion and exclusion criteria and anaemia definitions were different.

Parasites like the malaria parasites and intestinal parasites have long been recognized as major contributors to reduced haemoglobin levels in endemic countries like Cameroon, thereby causing anaemia. Also, PLWH on Zidovudine-containing ART have been reported to influence the haemoglobin level in previous studies [10, 11, 48-50]. In this study, patients infected with HIV, malaria and IPs had a significantly lower mean haemoglobin value (10.6 ± 1.21 g/dL). This is in line with previous studies which reported though among children, low haemoglobin values in patients with co-infections when compared with those with single infections [51-52]. Aside from well-reported side eff ects of anaemia associated with ZDV-containing ART regimen, though not confirmed in this study, anaemia among HIV infected individuals is more broadly multifactorial in origin, and three basic pathophysiologic mechanisms of anaemia in HIV infection have been postulated [47]: decreased RBC production, increased RBC destruction, and ineff ective RBC production.

A high prevalence of severe anaemia was observed among patients co-infected with MP and HIV. This is consistent with studies carried out in Nigeria [53-54], Ethiopia [49] and more recently in

Cameroon [11]. This confirms the possibility that co-infections may increase malaria morbidity, further worsening malaria disease severity [55]. In addition, triple, dual, HIV and malaria parasite infections as well as fever were reported as risk factors of anaemia in this study.

One strength of this study is that it fills some gaps on the occurrence of MP/IPs co-infection among PLWH in the region which had not been reported previously as well as the association between these infections and anaemia among this vulnerable group. Further evidence is demonstrated on the outcome of the efforts in management implemented by the government which are encouraging in reducing the HIV/AIDS morbidity in the various communities. Notwithstanding, this study had as limitations the fact that nutritional deficiency as a cause of anaemia was not investigated, as well as the cross-sectional nature of the study that provides a picture of the health situation at a point in time. Nevertheless these findings are valuable to policymakers in guiding the management of these conditions. However, future longitudinal studies on the impact of iron supplementation and vitamin B_{12} levels among PLWH may be of great relevance to understand the cause of the high anaemia prevalence observed.

Conclusions

From this study, it can be concluded that malaria parasite and intestinal parasites remain a public health concern among PLWH with higher occurrences of both MP, IPs and MP/IP co-infections than in their HIV negative counterparts in the communities. Though a good majority of the HIV patients in the study had attained viral suppression, they are still more vulnerable to some of the parasites than HIV negative individuals and therefore can constitute a good source of contamination in their various communities. To achieve HIV epidemic control in 2021, the Cameroon Government needs to intensify the implementation of the various strategies. Anaemia

stands as a serious haematological abnormality among HIV patients and gets exacerbated even with the viral load suppression in the population of PLWH. Hence, there is a need for constant monitoring of the health status as well as the nutritional status of this at-risk group to better manage them.

Acknowledgements

The authors thank all the participants (both children and adults) who took part in the study as well as the staff at the Unit in-charge of patients with HIV (UPEC) in the Buea Regional Hospital. We are also thankful to the Buea community inhabitants who consented and parents who consented for their children to participate in the study.

Authors' Contributions

Conceptualization: Sorelle Mekachie Sandie.

Data curation: Irene Ule Ngole Sumbele.

Formal analysis: Sorelle Mekachie Sandie.

I**nvestigation:** Sorelle Mekachie Sandie, Irene Ule Ngole Sumbele, Martin Mih Tasah, Helen Kuokuo Kimbi.

Methodology: Sorelle Mekachie Sandie, Martin Mih Tasah.

Supervision: Irene Ule Ngole Sumbele, Helen Kuokuo Kimbi.

Validation: Irene Ule Ngole Sumbele, Helen Kuokuo Kimbi.

Writing – original draft: Sorelle Mekachie Sandie.

Writing – review & editing: Irene Ule Ngole Sumbele, Helen Kuokuo Kimbi.

References

1. Bangert M. The cross-cutting contribution of the end of neglected tropical diseases to the sustainable development goals. Infect Dis Poverty 2017; 6:73.

2. UNAIDS: UNAIDS report on the global AIDS epidemic 2018, Geneva, Switzerland.

3. Country Operational Plan (COP), Cameroon 2019. Strategic Direction Summary. https://www.state.gov/wp-content/uploads/2019/09/Cameroon_COP19-Strategic-Directional-Summary_public.pdf Accessed 07 Apr 2020.

4. Mabbott NA. The influence of parasite infections on host immunity to co-infection with other pathogens. Front Immunol. 2018. 8;9:2579.

5. World Health Organization. World Malaria Report 2019. Cameroon, African Region https://www.who.int/malaria/publications/country-profiles/profile_cmr_en.pdf?ua=1 2019. Accessed 28 Feb 2020.

6. World Health Organization. World Malaria Report 2018. Cameroon, African Region https://www.who.int/malaria/publications/country-profiles/profile_cmr_en.pdf?ua=1 2018. Accessed 04 Mar 2020.

7. Nkuo-Akenji T, Tevoufouet EE, Nzang F, Fon E, Ebong IN. HIV/AIDS and malaria in pregnant women from Cameroon short running title: HIV, malaria in pregnancy. Afr J Health Sci. 2011;18:105-109.

8. Njunda LA, Kamga HLF, Nsagha DS, Assoh JCN, Kwenti TE. Low malaria prevalence in HIV-positive patients in Bamenda, Cameroon. J Microbiol Res. 2012;2:56-59.

9. Kimbi HK, Njoh DT, Ndamukong KJN, Lehman LG. Malaria in HIV/AIDS patients at different CD4 T cell levels in Limbe, Cameroon. J Bacteriol Parasitol. 2013;4 (164). doi: 10.4172/2155-9597.1000164.

10. Bate B, Kimbi HK, Lum E, Lehman LG, Onyoh EF, Ndip LM, et al. Malaria infection and anaemia in HIV-infected children in Mutengene, Southwest Cameroon: a cross sectional study. BMC Infect Dis. 2016;16,523.

11. Sandie SM, Sumbele IUN, Tasah MM and Kimbi HK. Malaria parasite prevalence and Haematological parameters in HIV seropositive patients attending the regional hospital Limbe, Cameroon: a hospital-based cross-sectional study. BMC Infect Dis. 2019;19, 988.

12. Malaria Operational Plan (MOP) FY 2018 and FY 2019. President's Malaria Initiative Cameroon, 2019. https://www.pmi.gov/docs/default-source/default-document-library/malaria-operational-plans/fy-2018/fy-2018-cameroon-malaria-operational-plan.pdf?sfvrsn=5 Accessed: 07 Apr 2020.

13. Alemu G, Alelign D, Abossie A. Prevalence of opportunistic intestinal parasites and associated factors among HIV Patients while Receiving ART at Arba Minch Hospital in South Ethiopia: A Cross-sectional Study. Ethiop J Health Sci. 2018; 28(2):147.

14. Webb EL, Ekii AO, Pala P. Epidemiology and immunology of helminth = HIV interactions. Curr Opin HIV AIDS. 2012;7:245–253.

15. Adeleke OA, Yogeswaran P, Wright G. Intestinal helminth infections amongst HIV-infected adults in Mthatha General Hospital, South Africa. Afr J Prim Health Care Fam Med. 2015;7(1): 910.

16. Nsagha DS, Njunda AL, Assob NJC, Ayima CW, Tanue EA, kibu OD et al. Intestinal parasitic infections in relation to CD4+Tcell counts and diarrhoea in HIV/AIDS patients with or without antiretroviral therapy in Cameroon. BMC Infect Dis. 2016;16:9.

17. Nacher M, Singhasivanon P, Yimsamran S, Manibunyong W, Thanyavanich N, Wuthisen R. Intestinal helminth infections are associated with increased incidence of Plasmodium falciparum malaria in Thailand. J Parasitol. 2002;88(1):55–8.

18. Sokhna C, Le Hesran JY, Mbaye PA, Akiana J, Camara P, Diop M. Increased malaria attacks among children presenting concomitant infections by *S. mansoni* in Senegal. Malar J. 2004;3:43.

19. Njunda AL, Fon SG, Assob JCN, Nsagha DS, Kwenti TDB and Kwenti TE. Co-infection with malaria and intestinal parasites, and its association with anaemia in children in Cameroon. Infect Dis Poverty. 2015;4:43.

20. Wagnew F, Eshetie S, Alebel A, Tesema C, Kibret GD, Gebrie A, et al. Burden of anemia and its association with HAART in HIV infected children in Ethiopia: a systematic review and meta-analysis. BMC Infect Dis. 2019;19.

21. Brooker S, Akhwale W, Pullan R, Estambale B, Clarke SE, Snow RW. Epidemiology of *Plasmodium*-helminth co-infection in Africa: populations at risk, potential impact on anemia and prospects for combining control. Am J Trop Med Hyg.2007;77(6S):88–98.

22. Sumbele IU, Nkemnji GB. and Kimbi HK. Soil-transmitted helminths and plasmodium falciparum malaria among individuals living in different agroecosystems in two rural communities in the mount Cameroon area: a cross-sectional study. Infect Dis Poverty. 2017;6(1): 67.

23. Saracino A, Nacarapa EA, Massinga EAC, Martinelli D, Oliveira C, Antonich A, et al. Prevalence and clinical features of HIV and malaria co-infection in hospitalized adults in Beira, Mozambique. Malar J. 2012; 11:241.

24. Sumbele IUN, Sama SO, Kimbi HK and Taiwe GS. Malaria, moderate to severe anaemia, and malarial anaemia in children at presentation to hospital in the Mount Cameroon Area: a cross-sectional study. Anemia. 2016;Article ID 572.

25. CAMPHIA (Cameroon Population-based HIV Impact Assessment) 2017. Summary sheet: Preliminary findings. https://phia.icap.columbia.edu/wp-content/uploads/2018/07/3471CAMPHIA_Cameroon-SS_A4 v13_requests_7.25.18.pdf 2017. Accessed 09 Apr 2020.

26. World Health Organization. Malaria Factsheets 2020. https://www.who.int/news-room/fact-sheets/detail/malaria 2020. Accessed 02 Apr 2020.

27. Charan J and Biswas T. How to calculate sample size for different study designs in medical research? Indian J Psychol Med. 2013;35(2) 121-126.

28. Chanyalew MD and Gurara MK. Malaria and intestinal parasite infections and co-infections in Tach Gayint District, South Gondar Zone, Amhara Regional State 2010. Science J Public Health. 2014; 2(6): 546-553.

29. Cheesbrough M. District Laboratory Practice in Tropical Countries, 3Rd ed., part 1. United Kingdom: Cambridge University Press; 2014. p. 178–215.

30. UNAIDS. Undetectable = Untransmittable. Publich health and HIV load suppression. 2018.https://www.unaids.org/sites/default/files/media_asset/undetectable-untransmittable_en.pdf Accessed 09 Apr 2020.

31. WHO Nutritional anaemias: tools for effective prevention and control. Geneva, Switzerland: World Health Organization Publication; 2017. https://www.who.int/nutrition/publications/micronutrients/anaemias-tools-prevention-control/en/. Accessed 07 April 2020.

32. Minsante. XIième Journée mondiale de lutte contre le paludisme "prêt à vaincre le paludisme" Nous sommes la génération qui peut éliminer le paludisme. Dossier de Presse. Minsante. 2018:1–20.

33. Mbah-Mbole FG, Tufon KA, Meriki DH, Enow-Orock G, Mbah-Mbole P, Njunda LA, et al.. Malaria and human immunodeficiency virus coinfection in febrile patients attending the Regional Hospital of Buea, Southwest region, Cameroon. Int J Adv Med Health Res. 2018;6:46-51.

34. Sanyaolu AO, Fagbenro-Beyioku AF, Oyibo WA, Badaru OS, Onyeabor OS, Nnaemeka CI. Malaria and HIV co-infection and their effect on haemoglobin levels from three healthcare institutions in Lagos, southwest Nigeria. Afr Health Sci. 2013; 13(2):295-300.

35. Njunda AL, Njumkeng C, Nsagha SD, Assob JCN and Kwenti, TE. The prevalence of malaria in people living with HIV in Yaounde, Cameroon. BMC Public Health. 2016; 16:964.

36. Bouyou-Akotet M, Offouga C, Mawili-Mboumba D, Essola L, Madoungou B, Kombila M: Falciparum malaria as an emerging cause of fever in adults living in Gabon, Central Africa. BioMed Res Int. 2014; Article ID 351281.

37. Lehman LG, Kangam L, Nguepi E, Mbenoun ML and Bilong Bilong CF. Study of intestinal parasitic infections associated with HIV infection in Douala, Cameroon. Retrovirology. 2012;9 (Suppl 1): 48.

38. Nkenfou CN, Nana CT and Payne VK. Intestinal parasitic infections in HIV infected and non-infected patients in a low HIV prevalence region, West- Cameroon. PLoS ONE. 2013;8(2): e57914.

39. Vouking MZ, Enoka P, Tamo CV, Tadenfok CN. Prevalence of intestinal parasites among HIV patients at the Yaoundé Central Hospital, Cameroon. Pan African Med J. 2014;18:136.

40. Kwenti TE, Nkume FA, Tanjeko AT and Kwenti TDB. The effect of intestinal parasitic infection on the clinical outcome of malaria in coinfected children in Cameroon. PLoS Negl Trop Dis. 2016; 10(4): e0004673.

41. Angora KE, Djohan V, Konaté A, Kiki-Barro PC and Bedia-Tanoh AV. Uncomplicated malaria and intestinal helminth co-infections among schoolchildren in Abobo District, Abidjan (Côte d'Ivoire). J Infect Dis Epidemiol. 2019;5:064.

42. Assefa M, Abegaz WE and Mulugeta Belay. Prevalence and correlates of anemia among HIV infected patients on highly active anti-retroviral therapy at Zewditu Memorial Hospital, Ethiopia. BMC Hematol. 2015; 15: 6.

43. Ako SE, Njunda LA, Akum EA, Benjamin PT, Assob JC. Hematological related disorders and transfusion of HIV Patients on Highly Active Antiretroviral Therapy (HAART) in the South West Region of Cameroon: Hematological Monitory Parameters for HIV Follow-Up. J HIV Retrovirus.2018;4;1:5.

44. Lai JL, Chen YH, Liu YM, Yuan JJ, Lin J, Huang AQ et al. Prevalence and risk factors of anaemia in hospitalised HIV-infected patients in southeast China: a retrospective study. Epidemiol Infect 2019; 147:e81.

45. Gebremedhin KB and Haye TB. Factors associated with anemia among people living with HIV/AIDS taking art in Ethiopia. Hindawi Advances in Hematology. 2019; 8.

46. Ageru TA, Koyra MM, Gidebo KD, Abiso TL. Anemia and its associated factors among adult people living with human immunodeficiency virus at Wolaita Sodo University teaching referral hospital. PLoS ONE. 2019;14(10): 0221853.

47. Volberding PA, Levine AM, Dieterich D, Mildvan D, Mitsuyasu R, Saag M et al. Anemia in HIV Working Group. Anemia in HIV Infection: Clinical impact and evidence-based management strategies. Clin Infect Dis. 2004;38:1454–63.

48. Blake M and Sherer R. Management of the adverse effects of antiretroviral therapy and medication adherence. Clin Infect Dis. 2000;30(Suppl2):S96–116.

49. Ojurongbe O, Oyeniran O, Alli O, Taiwo S, Ojurongbe T, Olowe A, et al. Prevalence of *Plasmodium falciparum* parasitaemia and its correlation with haematological parameters among HIV-Positive individuals in Nigeria. J Trop Med Hyg. 2014:161284.

50. Breymann C. Iron deficiency anemia in pregnancy. Semin Hematol. 2015; 52(4):339–47.

51. Tay SC, Badu K, Mensah AA. and Gbedema SY. The prevalence of malaria among HIV seropositive individuals and the impact of the co- infection on their hemoglobin levels. Ann Clin Microbiol Antimicrob. 2015;14, 10.

52. Njua-Yafi C, Achidi EA, Anchang-Kimbi JK, Apinjoh TO, Mugri RN, Chi HF, et al. Malaria, helminths, co-infection and anaemia in a cohort of children from Mutengene, south western Cameroon. Malar J. 2016; 15:69.

53. Erhabor O, Babatunde S, and Uko KE. Some haematological parameters in Plasmodial parasitized HIV-infected Nigerians. Nigeria J Med. 2006; 15(1): 52-55.

54. Etusim PE, Ihemanma CA, Nduka FO, Melariri PE, Ukpai O. Comparative study on the haematological characteristics of malaria infected and malaria non-infected persons referred to art/HIV laboratory, Abia State University teaching hospital, aba, Abia state. Int J Sci Multidiscip Studies. 2013;5(1):100–12.

55. Rattanapunya S, Kuesap J, Chaijaroenkul W, Rueangweerayut R, and Na-Bangchang K. Prevalence of malaria and HIV coinfection and influence of HIV infection on malaria disease

severity in population residing in malaria endemic area along the Thai-Myanmar border. Acta Tropica. 2015:145:55

Figure

Click here to access/download;Figure;Fig2.tif

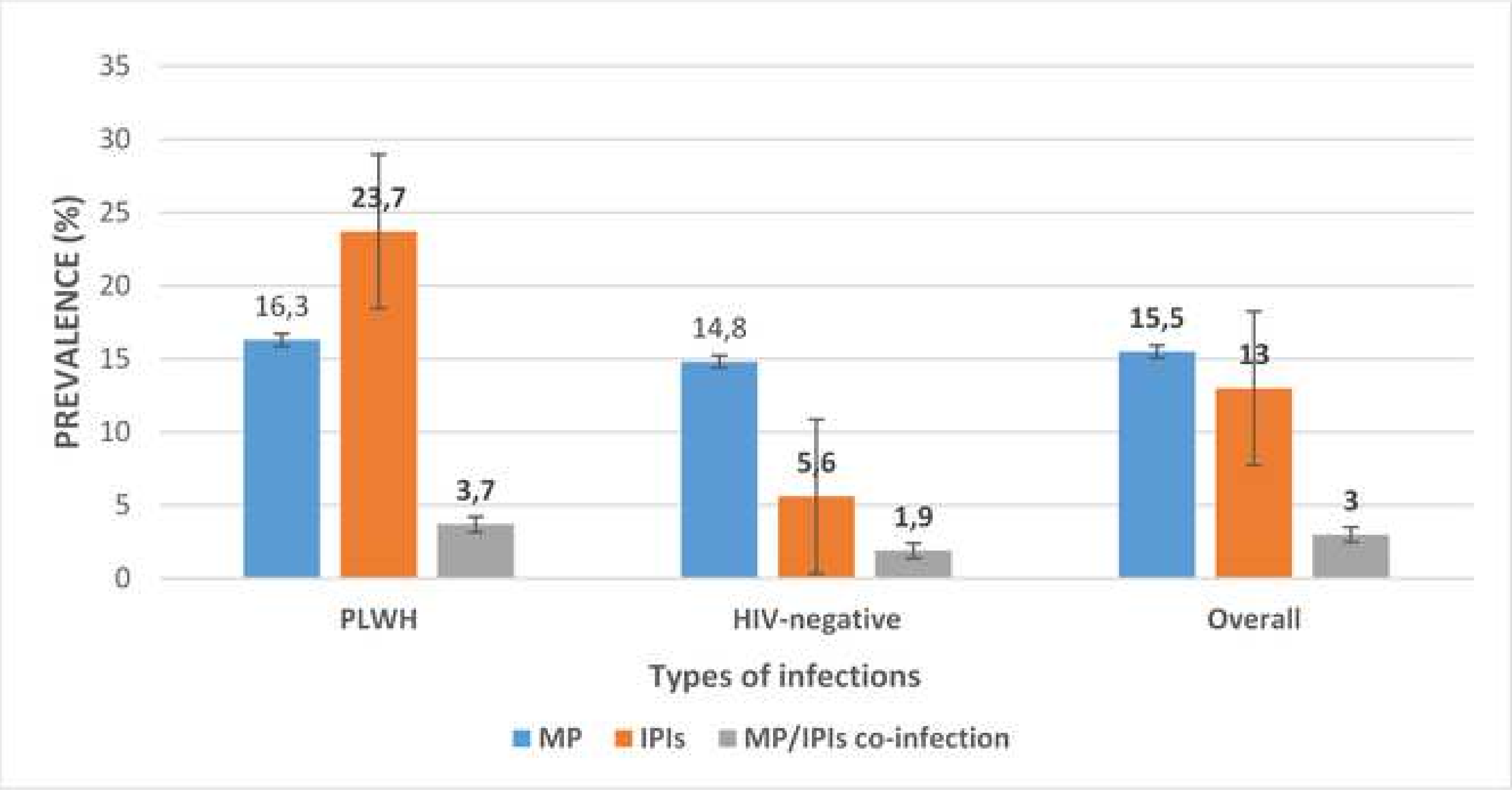

Figure

Click here to access/download;Figure;Fig3.tif

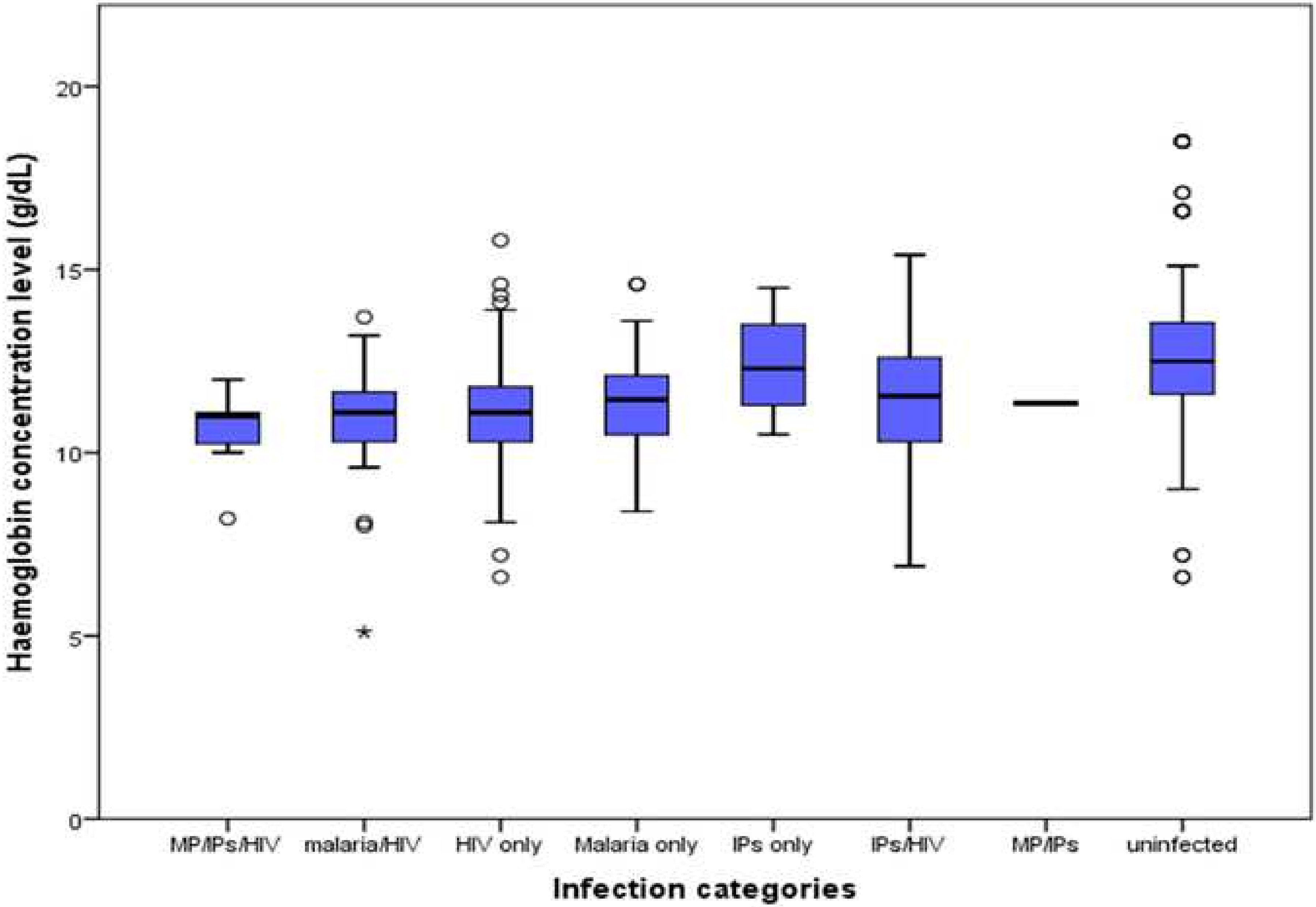

Figure

Click here to access/download;Figure;Fig1.tif

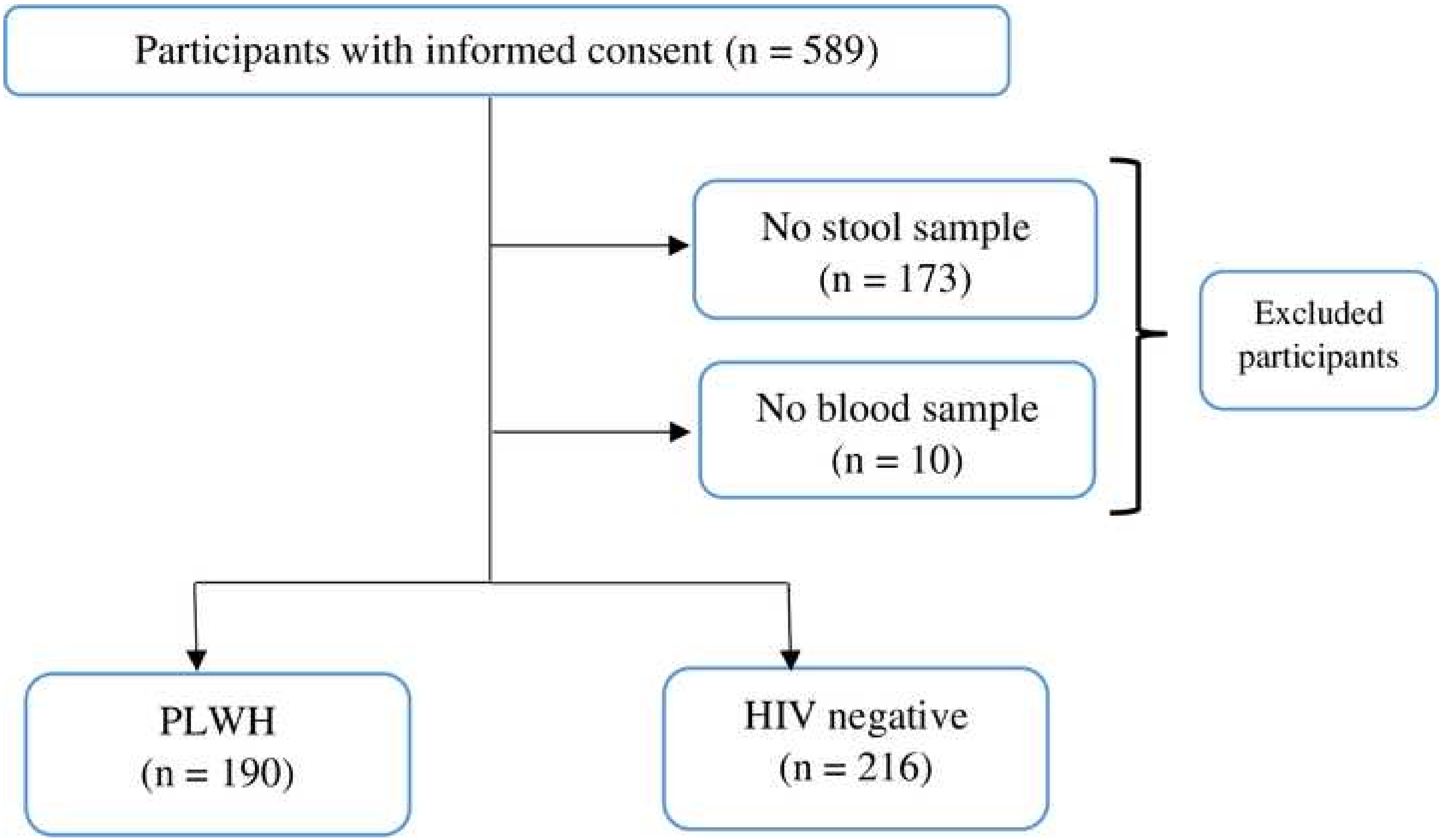

Click here to access/download
Supporting Information
Questionnaire.docx

Malaria and intestinal parasite co-infection and its association with anaemia among people living with HIV in Buea, Southwest Cameroon: A community-based cross-sectional study

Sorelle Mekachie Sandie[1*¶], Irene Ule Ngole Sumbele[1, 2¶], Martin Mih Tasah[1] and Helen Kuokuo Kimbi[1,3¶]

1 Department of Zoology and Animal Physiology, University of Buea, Buea, Cameroon.

2 Department of Microbiology and Immunology, Cornell College of Veterinary Medicine, Ithaca, New York, USA.

3 Department of Medical Laboratory Science, The University of Bamenda, Bambili, Cameroon

*Corresponding author

E- mail: **sandiesorelle2@gmail.com** **(SMS)**

[¶]These authors contributed equally to this work.

Abstract

Background: Both malaria and intestinal parasites are endemic in Cameroon, and their co-infection can be of great impact on anaemia among people living with HIV (PLWH). This community-based cross-sectional study determined the prevalence and impact of infections on anaemia in PLWH and HIV-negative individuals in Buea, Cameroon from March to August 2019.

Methods: The study population comprised of 190 PLWH and 216 consenting HIV negative individuals from the Buea community. Participants were examined clinically, the collected blood sample was used for malaria parasite (MP) detection and speciation, HIV diagnosis and haemoglobin (Hb) measurement while stool samples were examined for the detection of intestinal parasites (IPs). Proportions were compared using Pearson's Chi-square test and association of anaemia with independent variables was evaluated using logistic regression analysis.

Results: Out of the 406 participants, MP, IPs and MP/IP co-infection prevalences were 15.5%, 13.0% and 3.0% respectively. When compared with HIV negative participants, PLWH had a higher prevalence of MP (16.3%, $P = 0.17$), IPs (23.7%, $P < 0.001$) and MP/IPs co-infection (3.7%, $P = 0.04$). Participants with triple infections co-infected with HIV, MP and IP had a significantly lower mean haemoglobin value (10.6 ± 1.21 g/dL). When comparing single infections, the prevalence of anaemia was highest among those who had HIV only (79.3%) when compared with those infected with MP only (71.4%) and IPs only (25.0%). HIV positive patients had a significantly ($P < 0.001$) higher prevalence of mild (56.8%), moderate (18.4) and severe (1.6%) anaemia when compared with HIV negative counterparts. The significant risk factors associated with anaemia included being febrile ($P = 0.03$), MP-infected only ($P = 0.001$), HIV-infected only ($P < 0.001$), having dual ($P < 0.001$) or triple-infections ($P = 0.03$).

Conclusion: Multiple infections exacerbate the health condition of PLWH. Routine medical check-ups among PLWH are recommended.

Background

Parasitic infections are a major cause of morbidity and mortality in Africa; especially in resource-limited tropical and sub-tropical regions like sub-Saharan Africa [1] where the majority of HIV/AIDS cases are concentrated. In 2018, there were roughly 1.7 million new HIV infections, which is an insignificant improvement when compared to 2017 (1.8 million) [2]. The same year, Cameroon reported an HIV incidence of 1.02 and a national prevalence of 3.6% [2]. People living with HIV (PLWH) are threatened by a great number of diseases caused by different types of biological agents. Although the morbidity and mortality due to malaria and intestinal parasites can be controlled by the application of various prevention measures put in place by the Cameroon government as well as the delivery of chemotherapy, elimination and final eradication will not be achieved until populations live in mosquito-free environments and have access to effective sanitation, sewage treatment and waste disposal; which remain a trending problem in Cameroon.

HIV infection is characterized by immunosuppression that exposes the infected individual to a variety of microbial and parasitic infections such as *Plasmodium*, *Mycobacterium tuberculosis* and intestinal parasitic infections [3]. These infections (opportunistic or not) are a serious threat to the health status of people living with HIV, especially in developing countries like Cameroon. In an attempt to achieve HIV epidemic control in 2021, the Cameroon Government has been implementing various strategies that led to an encouraging increase in antiretroviral therapy (ART) coverage (53%) and viral load (VL) suppression (44.7%) nationally in 2019 [4].

African countries still bear the largest burden of malaria morbidity (93%), with *Plasmodium falciparum* accounting for 99.7% of malaria cases in 2018 [5]. In 2018, WHO reported that Cameroon featured the fourth country behind Nigeria, Democratic Republic of Congo and Mozambique with the highest malaria prevalence [6]. Many studies have consistently reported an association between HIV infection and malaria in Cameroon [7, 8, 9, 10, 11], with each region showing a varied prevalence of both malaria and HIV infection. Malaria transmission is stable throughout the year, with an overall malaria test positivity rate of 62% registered in 2017 [12].

The burden of intestinal parasite infections (IPIs) is greatest in low-income areas and are major public health problems in certain areas of Sub-Saharan Africa. These IPIs are associated with a humid climate, unsanitary environments and poor socio-economic conditions. Parasites causing these IPIs are protozoans (*Cryptosporidium species, Cyclospora cayetanensis, Isospora belli, Microsporidia spp* and *Entamoeba histolytica*) and helminths (*Ascaris lumbricoides, Trichuris trichuiria,* hookworms, *Schistosoma mansoni* and *Taenia species*). *Cryptosporidium species, Cyclospora cayetanensis*, *Isospora belli*, and *Microsporidia spp* are often referred to as opportunistic parasites in people living with HIV because they cause great morbidity especially when CD4 T cell count falls below 200 cells/µL [13]. Various studies carried out among African adults living with HIV have reported IPI rates ranging from 10% to 45% [14, 15]. Majority of the research on the impact of IPIs on HIV progression in Cameroon, occurred before the wide availability of ART [16] hence, the need for further evaluation.

In Cameroon, PLWH face similar environmental realities as those not infected with HIV and so can easily get co-infected with combinations of intestinal and malaria parasites. Such co-infections may have considerable health consequences which could lead to severe clinical symptoms and pathology in PLWH. Studies have shown that individuals with multiple infections have an increased risk of developing more frequent and severe diseases due to interactions among infecting parasite species [17-19].

Malaria and intestinal parasites in PLWH can both lead to severe haematological abnormalities. Although ART in combination with cotrimoxazole confers a level of immunity to infections in PLWH, it has been reported to have adverse effects on the red blood cell indices of these patients, thereby causing anaemia [20]. Although malaria and intestinal parasites, as well as ART, are known etiological factors in anaemia [11, 16, 21, 22], the extent to which their interaction might enhance the risk of anaemia merits further investigations. Previous studies have reported on the influence of malaria/HIV co-infection on the occurrence of anaemia [11, 19, 23]. Co-infections in HIV are numerous and important causes of morbidity and mortality. Combating co-infections has been identified as an important public health goal. The situation regarding PLWH co-infected with malaria and intestinal parasites in the area following the intensification of different control strategies such as free availability of ART and cotrimoxazole, access to viral load testing and community ART dispensation is unknown. Therefore, the present study was undertaken to determine the prevalence of malaria parasite and intestinal parasites in PLWH compared to HIV negative counterparts as well as to assess the effects of these infections on anaemia to provide relevant information to policymakers in the country.

Methods

Study area and participants

The study was carried out in the HIV Treatment and Care Centre at the Regional Hospital and different neighbourhoods in the Buea community. The study area has been described in detail by Sumbele et al [24]. The Buea Regional Hospital is the referral HIV treatment centre in Fako Division serving the population and hence receives HIV positive patients daily from the various neighbourhoods for their monthly distribution of ART. With the presence of the Mount Cameroon, Buea is a major touristic site and has a business-friendly environment. In the national HIV prevalence ranking, the South West Region is the 6th Region with a prevalence of 3.6% which equates the national prevalence (3.7%) [25] as well as the second to the last Region (33.8%) with the least viral load suppression. Buea is an area of moderate malaria transmission, with a peak time for malaria transmission during and just after the rainy season (March to September) [26]. Its tropical climate provides a suitable environment for the development of the malaria vectors and this therefore, contributes to increased malaria transmission. There is also inadequate access to safe water in this region, predisposing the population to water-borne infections including IPIs.

The two categories of participants, PLWH and HIV-negative individuals were enrolled in the study at the HIV Treatment and Care Centre and from the different neighbourhoods. Only participants who signed the informed consent form and willingly accepted to be counselled and tested for malaria, IPIs and HIV were enrolled in the study. Participants on antibiotics, malaria treatment or antiparasitic agents two weeks before enrolment were excluded from the study.

Study design and sample size

This study was a community-based cross-sectional study carried out between April 2018 and August 2019. HIV-negative participants included persons who consented to be screened for HIV during the process and were negative. A convenience method of sampling was employed and participants were enrolled through HIV outreach programs in their homes and offices in the Buea neighbourhoods (Molyko, Bonduma, Great Soppo, Government reserve area (GRA), Buea town, Bokwaongo, Bokova, Mile 18, Clerks and Federal quarters). The sample size was determined using the formula $N=2(Z_\alpha + Z_\beta)^2 P(1-P)/(P_0-P_1)^2$ [27] where N represented the sample size evaluated, Z_α was 1.96 and Z_β was 0.84 which are the standard normal deviates (for the 95% confidence interval, CI), P_0 (Previous prevalence of MP/IPs co-infection in PLWH) was 50% and P_1 (Previous prevalence of MP/IPs co-infection in HIV negative individuals) was 67.2% [28]. The sample size calculated was 128 for each group giving an optimum sample size of 256 participants for the study.

Data collection

Questionnaire

A pre-structured questionnaire was used to collect socio-demographic information such as age, sex, location of residence, level of education, marital status and occupation while clinical data such as ART usage, duration on treatment, ART regimen and viral load (VL) were collected from each HIV patient's file at the HIV Treatment and Care Centre. Health and preventive practices towards malaria and IPIs as well as behavioural habits regarding health conditions with a history of symptoms were recorded.

Sample collection and processing

Participants were instructed on how to collect stool sample in universal transparent faecal containers with a screw cap lid. After the provision of a stool sample, the participant's body temperature was measured using a digital thermometer and fever was defined as temperature ≥ 37.5^{o}C. Blood was collected under sterile conditions into a well-labelled Ethylenediaminetetraacetate (EDTA) tube for each participant for HIV screening and confirmation, malaria diagnosis and haemoglobin (Hb) measurement. The collected samples were transported to the Malaria Research Laboratory of the University of Buea for further analysis.

On arrival in the laboratory, the stool samples were analysed macroscopically and preserved in 10% formalin. Formol-ether concentration technique and Modified Ziehl-Neelsen (ZN) methods [29] were performed for the detection of intestinal parasites (protozoans and helminths). To estimate parasite intensity, egg counts per slide were converted to egg per gram of faeces (epg) by multiplying the number of eggs on the slide by 24. The blood sample was used to prepare thick and thin blood films on a labelled slide that was Giemsa-stained, analysed following standard procedures and the parasite density per microliter of blood was calculated based on the number of parasites per 200 leucocytes on the thick blood film, assuming a leucocyte count of 8000 leucocytes/µL of blood as described by Cheesbrough [29]. All blood and stool slides were examined by two microscopists who were blinded to the results, and where positive/negative discrepancy occurred, slides were counter-read by a third microscopist, who was blinded regarding the two previous results. A negative result was declared after checking at least 200 microscope fields. The blood was also used for HIV rapid test (in the case of presumed HIV-negative participants) and Hb measurement. Viral loads were obtained from the HIV positive participant's file at the HIV Care Centre. Viral load < 50 copies /mL of blood was considered undetectable [30].

CD4T cell counts were determined using the BD FACS Count™ System (BD Biosciences, USA) as per the manufacturer's instructions. To assure the quality of the laboratory data, standard operating procedure for each test was followed. Reagents were checked for expiry date and prepared according to the manufacturer's instruction. The Determine Rapid Test Kit (Abott Laboratories, CO., Ltd. Minato-Ku, Tokyo Japan) was used to test the supposed HIV negative subjects for HIV-1 following the manufacturer's instructions.

Haemoglobin (Hb) measurement

The full blood count (FBC) analysis was carried out using the URIT-3300 automated haematology analyser (URIT Medical Electronic CO., LTD. Jiuhua Road, Gungxi, China), following the manufacturer's instructions. Haemoglobin concentration was obtained from the FBC results. Anaemia was defined as Hb concentrations below the World Health Organization (WHO) reference values corresponding to age or gender [31]. Further classification was done to determine severe (Hb < 7g/dL), moderate (Hb between 7.0 and 10.0 g/dL) and mild anaemia (> 10 g/dL Hb < 11g/dL) cases [29].

Statistical Analysis

Data were analysed using IBM-Statistical Package for the Social Science (SPSS) version 20.0 software (SPSS Inc., Illinois, USA). Baseline characteristics were summarized as mean with 95% CI and compared using the independent *t*-test. Malaria and intestinal parasites data were summarized into means and standard deviation (SD) and percentages were used in the evaluation of the descriptive statistics. The significance of differences in prevalence was explored using Pearson's Chi-square while Analysis of Variance (ANOVA) and Student's t-test was used where

appropriate to assess difference in group means. The association of anaemia with the independent variables was evaluated using logistic regression model. $P < 0.05$ was considered for statistical significance.

Ethical considerations

Using an information sheet, a brief talk was given to the participants in the English Language (and exceptionally in Pidgin English) and the participants were then invited to participate in the study by signing an informed consent form. In the case of children (≤ 15years), their parents signed proxy informed consent form on their behalf. Ethical clearance was obtained from the Institutional Review Board hosted by the Faculty of Health Sciences of the University of Buea (Reference Number: 661-09). For confidentiality purposes, patients were identified by special identification numbers. Results of participants with parasitic infections in the neighbourhoods were referred to a clinician for their case management.

Results

Socio-demographic and clinical characteristics of the participants

Five hundred and eighty-nine (589) participants were approached to participate in the study, those who could not provide stool sample and whose blood sample could not be collected were excluded from the study; giving a total of 406 (68.9%) successfully enrolled participants. Out of the 406 participants, 190 were HIV positive patients from the HIV Treatment and Care Centre and 216 were HIV-negative from the communities as shown in Fig 1.

Fig 1: Study flow diagram showing compliance of the participants

Overall, the mean ± SD age of the participants was 31.2 ± 16.7 (range = 1–70) years. There were more females (68.5%) than males (31.5%). A greater proportion of the study population belonged to the > 40 years age group (31.0%) and the least to the 31–40 years age group (18.7%). Majority of the PLWH had primary level of education (66.7%), were self-employed (31.1%) and were single (62.0%). Among HIV negative participants, majority had university level of education (35.2%), were students (56.5%) and single (63.0%) as revealed in Table 1. Less than half of the study population (45.6%) used ITNs as a preventive measure against malaria. A greater proportion of PLWH used ITNs (57.4%) more than the HIV negative counterparts (35.2%). As seen in Table 1, there were statistically significant differences in the various variables between HIV+ and HIV- participants apart from marital status.

Table 1. Characteristics of the study population

Parameters		HIV Positive n (%)	HIV Negative n (%)	Overall n (%)	χ^2 P-value
**Age groups (years)**	≤ 20	42 (22.1)	72 (33.3)	114 (28.1)	48.64 < 0.001*
	21-30	20 (10.5)	70 (32.4)	90 (22.2)	
	31-40	48 (25.3)	28 (13.0)	76 (18.7)	
	> 40	80 (42.1)	46 (21.3)	126 (31.0)	
**Mean age ± SD (years)**		35.5±16.5	27.6±15.8	32.3±16.7	
**Sex**	Female	140 (73.7)	138 (63.9)	278 (68.5)	4.49 0.02*
	Male	50 (26.3)	78 (36.1)	128 (31.5)	
	No formal	6 (3.7)	18 (8.6)	24 (6.0)	

**Educational Level**	Primary	126 (66.3)	54 (25.7)	180 (45.1)	
	Secondary	43 (22.6)	64 (30.5)	107 (26.8)	78.94
	Tertiary	14 (7.4)	74 (35.2)	88 (22.1)	< 0.001*
**Occupation**	Student	48 (25.4)	122 (56.5)	171 (42.1)	
	Unemployed	25 (13.2)	12 (5.2)	37 (9.1)	45.38
	Self-employed	59 (31.2)	28 (13.0)	87 (21.4)	< 0.001*
	Employee	57 (30.2)	54 (25.0)	111 (27.3)	
**Marital status**	Single	93 (62.0)	102 (63.0)	197(62.5	0.03
	Married	61 (38.0)	60 (37.0)	117(37.5)	0.47
**ITN usage**	Yes	109 (57.4)	76 (35.2)	185 (45.6)	20.05
	No	81 (42.6)	140 (64.8)	221 (54.4)	< 0.001*

Axillary temperature measurement revealed that 14.0% (57) of the general population were feverish, with the highest proportion (15.7%) among HIV negative participants. Malaria clinical signs (headache, joint pains, nausea, vomiting etc…) were less frequent among HIV negative participants than among PLWH (23.4%). Anaemia occurred in 57.1% (232) of the participants and PLWH had the highest occurrence (76.8%) when compared with their HIV negative counterparts and the difference was statistically significant ($\chi^2 = 56.59$, $P < 0.001$). Among PLWH, half of the population (50.0%) had CD4 T cell count between 200-500 cells/µL of blood, the majority (97.9%) were on ART, 91.9% (171) were taking zidovudine-based ART and 77.5% (141) had attained viral suppression(< 50 copies/mL of blood) as shown in Table 2.

Table 2. Clinical characteristics and laboratory profile of the study population

Parameters		HIV Positive n (%)	HIV Negative n (%)	Overall n (%)	χ^2 P-value
**Fever status**	Febrile	23 (12.1)	34 (15.7)	57 (14.0)	1.12
	Afebrile	167 (87.9)	182 (84.3)	349 (86.0)	0.18
**Mean temperature ±SD (°C)**		37.1 ±0.5	37.1 ±0.5	37.1 ±0.5	
**Malaria clinical signs**	Yes	39 (23.5)	32 (18.2)	71 (20.7)	1.40
	No	127 (76.5)	144 (81.8)	272 (79.3)	0.15
**Anaemic status**	Anaemic	146 (76.8)	86 (39.8)	232 (57.1)	56.59
	Non-anaemic	44 (23.2)	130 (60.2)	174(42.9)	$< 0.001^*$
**Mean Hb ±SD** (g/dL)		11.1±1.5	12.5 ±2.1	11.8 ±1.9	
**CD4 T-cell count(cells/µL)**	< 200	15 (14.2)	-	15 (14.2)	-
	200 – 500	51 (48.1)	-	51 (48.1)	
	>500	40 (37.7)	-	40 (37.7)	
**Mean CD4 T cell count ±SD (cells/µL)**		466.0 ±275.9	-	466.0±275.9	
**ART usage**	Yes	186 (97.9)	-	186 (97.9)	-
	No	4 (2.1)	-	4 (2.1)	
**Zidovudine-based ART**	Yes	171 (91.9)	-	171 (91.9)	-
	No	15 (8.1)	-	15 (8.1)	
**ART Duration (years)**	0	4 (2.1)		4 (2.1)	-
	< 1	36 (19.4)	-	36 (19.4)	
	1 -3	61 (32.8)	-	61 (32.8)	
	>3	89 (47.8)	-	89 (47.8)	
**Viral load (RNA copies/ml)**	Not detectable (<50)	141 (77.5)	-	141 (77.5)	-
	≥ 50	41 (22.5)	-	41 (22.5)	

Malaria parasite, intestinal parasite infection and MP/IPI co-infection prevalence

Out of the 406 participants, 63 (15.5%) were positive for MP with *Plasmodium falciparum* as the only species observed. MP prevalence was higher in HIV positive participants (16.3%) than those negative (14.8%) although not statistically significant (P = 0.39). Overall, 13.0% (65) of the participants were positive for IPIs with a higher occurrence of protozoa (10.4%) than helminths (3.8%). The most prevalent protozoa parasite was *Cryptosporidium* species while that of helminths was hookworm. The prevalence of IPs was significantly higher ($\chi^2 = 31.73$, $P < 0.001$) among PLWH (23.7%) than in those negative (5.6%). Malaria/IPs co-infection prevalence was 2.7% (11) and was comparable (P = 0.20) among PLWH (3.7%) and HIV negative participants (1.9%) as shown in Fig. 2.

Fig 2: Prevalence of MP, IPIs and MP/IPIs co-infection in the study population

Infection categories, haemoglobin level and anaemia prevalence

Out of the 406 participants, 1.7% (7) had triple infection (MP, HIV and IPIs), 9.4% (38), 5.9% (24) and 1.0% (4) were co-infected with IPIs/HIV, MP/HIV and MP/IPIs respectively. Equally, 29.8% (121) had HIV only, 6.9% (28) had malaria only and 2.0 (8) had IPIs only. The rest of the participants (43.3%, 176) were uninfected. As shown in Fig 3, there was a significant difference ($H = 68.24$, $P < 0.001$) when comparing mean haemoglobin values between the different infection categories; as those triple infected had the lowest mean haemoglobin value (10.55 ± 1.20g/dL) when compared with dual and single infected patients accordingly.

Fig. 3: Box and whisker plots showing mean haemoglobin levels by infection categories

The prevalence of anaemia in the study population was 57.1% (232) and PLWH had a significantly higher (χ^2= 56.59, P < 0.001) prevalence of anaemia (76.8%) than their HIV negative counterparts (39.8%). When comparing single infections, the prevalence of anaemia was highest among those who had HIV only (79.3%), followed by those infected with malaria parasite only (71.4%) and then those with IPs only (25.0%) and the difference was statistically significant (P = 0.002). Participants infected with MP, HIV and IPs simultaneously were observed to have the highest prevalence of anaemia (85.7%) when compared with other co-infection categories as shown in Table 3.

With respect to anaemia severity, HIV positive patients had a significantly (P < 0.001) higher prevalence of mild (56.8%), moderate (18.4) and severe (1.6%) anaemia when compared with HIV negatives. Severity of anaemia with respect to infection categories is seen in Table 3.

Table 3. Prevalence of anaemia, anaemia severity and mean (SD) Hb levels by infection category

Infection category	Number examined N	Prevalence of anaemia n (%)	Mean (SD) Hb (g/dL)	Prevalence of anaemia severity		
				Mild anaemia n (%)	Moderate anaemia n (%)	Severe anaemia n (%)
**HIV positive**	190	146 (76.8)	11.11 (1.54)	108 (56.8)	35 (18.5)	3 (1.6)
**HIV negative**	216	86 (39.8)	12.45 (2.06)	72 (33.3)	12 (5.6)	2 (0.9)
**Test statistic**	χ^2= 56.59, P < 0.001*		U = 29316 P < 0.001*	χ^2= 58.74, P < 0.001*		
**HIV only**	121	96 (79.3)	11.06 (1.44)	72 (59.5)	23 (19.0)	1 (0.8)
**MP only**	28	20 (71.4)	11.57 (1.52)	18 (64.3)	2 (7.1)	0 (0.0)

**IPs only**	8	2 (25.0)	12.40 (1.52)	2 (25.0)	0 (0.0)	0 (0.0)
**Test statistic**	χ^2= 12.12, P = 0.002*		H = 6.87 P < 0.001*	χ^2= 14.47, P = 0.02*		
**MP/HIV**	24	19 (79.2)	10.83 (1.81)	15 (62.5)	3 (12.5)	1 (4.2)
**HIV/IPs**	38	25 (65.8)	11.49 (1.68)	17 (44.7)	7 (18.4)	1 (2.6)
**MP/IPs**	4	2 (50.0)	11.35 (0.06)	2 (50.0)	0 (0.0)	0 (0.0)
**MP/HIV/IPs**	7	6 (85.7)	10.55 (1.20)	4 (57.1)	2 (28.6)	0 (0.0)
**Test statistic**	χ^2= 2.88, P = 0.83		H = 3.84 P = 0.28	χ^2= 5.07, P = 0.82		

H: Kruskal Wallis test; U Mann Whitney U test.

Risk factors of anaemia

A logistic regression model was used to determine the factors associated with anaemia prevalence. The model revealed sex (P < 0.001), age group (P = 0.003), fever status (P = 0.035), infection with HIV only (P = 0.024), infection with MP only (P = 0.004) as well as HIV/MP co-infection (P = 0.027) as significant predictors of anaemia in the study population (Table 4). Male participants and those aged 21–30 years of age were respectively 3 and 2 times more likely to be anaemic than their respective counterparts.

Table 4: Logistic regression model examining factors that influence the prevalence of anaemia in the study population

**Variables**	**Bivariate**		**Multivariate**	
	**COR**	**P-value**	**AOR**	**P value**
**Age group (years)**				
**≤ 20**	1.18	0.521	-	-
**21 – 30**	1.79	0.032*	-	-

**31 – 40**	0.92	0.771	-	-
**> 40**	Reference	-	-	-
**Sex**				
**Male**	2.68	< 0.001*	0.37	< 0.001*
**Female**	Reference	-	reference	-
**Febrile status**				
**Febrile**	0.52	0.035*	1.92	0.010*
**Non-febrile**	Reference	-	reference	-
**Single infection**				
**HIV only**	0.13	0.024*	0.18	0.020*
**MP only**	0.08	0.004*	0.10	0.003*
**IP only**	Reference	-	reference	-
**Dual infection**				
**HIV/MP**	0.31	0.027*	-	-
**HIV/IP**	0.63	0.192	-	
**MP/IP**	1.21	0.851	-	-
**None**	Reference	-	-	-
**Triple infection (HIV/MP/IP)**				
**Yes**	0.30	0.282	-	-
**No**	Reference	-	-	-
**Suppressed viral load**				
**Yes**	1.26	0.600	-	-
**No**	Reference	-	-	-
**Zidovudine-based ART**				
**Yes**	0.78	0.695	-	-
**No**	Reference	-	-	-
**CD4 T-cell count (cells/µL)**				
**< 200**	0.35	0.212	-	-
**200–500**	0.64	0.361	-	-
**> 500**	Reference	-	-	-

*Statistically significant P-value
The test used in the logistic regression was the Likelihood Ratio test.

Discussion

Parasitic infections (especially malaria and intestinal parasites) are major public health concerns among PLWH in developing countries, particularly Sub-Saharan Africa which has been reported to have the highest burden of HIV [2]. This study was a community-based cross-sectional study carried out to determine the prevalence of malaria and intestinal parasites and its association with anaemia among PLWH and HIV negative individuals in Buea, in the South West Region of Cameroon.

In the present study, the overall prevalence of malaria parasite was 15.5%, irrespective of the HIV status of the participants. This result is lower than the recently reported national prevalence (24.4%) [32]. This decrease could be attributed to the restless efforts of the Cameroon Government over the years to implement malaria control strategies to curb down malaria morbidity and mortality on the entire territory; knowing that Cameroon comes fourth on the list of countries that account for the burden of malaria in the WHO African Region [6].

The prevalence of malaria parasite among PLWH was 16.3%, which is high compared to the prevalence reported in PLWH in other areas of the South West Region of Cameroon. This includes the 7.8% reported in Limbe [11] and 2.3% in Buea [33] in 2019 and 2018 respectively. It was also observed in this study that despite the efforts made to scale up insecticide-treated nets (ITNs) distribution so that universal coverage can be attained, coverage remains low (57.4%) and this

could have contributed to this rise in malaria prevalence among PLWH. Another reason for this difference could be the difference in the age distribution of the study population.

The prevalence of malaria parasite was slightly higher in HIV positive patients than in their HIV negative counterparts in line with findings in studies carried out in Nigeria [34]. Interactions between malaria and HIV have been studied extensively, which consider HIV positive patients as a high-risk group for malaria in endemic areas like Cameroon [10, 11, 33, 35]. This slight difference is not significant and this could be as a result of the government actions through the availability of different ACTs and ITNs for both PLWH and HIV-negative Cameroonians; as well as the increasing availability and rapid scale-up of ART and cotrimoxazole to HIV patients in particular. It is worth noting that the HIV negative participants in the communities with malaria parasite are asymptomatic carriers and could greatly fuel the transmission of the malaria parasite in the community. However, the low prevalence shows that strategies implemented by the Cameroon government over the years are effective as this prevalence is lower than the reported national prevalence of malaria (29%) in 2018 [6].

The febrile status of most of the participants in the study irrespective of their HIV status was attributed to the presence of malaria parasite. This is in line with other studies carried out in Cameroon [11] and in Gabon [36]. Also, feverish PLWH had the highest prevalence of MP/IPs co-infection asserting the fact that fever remains an important feature in the clinical diagnosis of infections. Fever is one of the most common clinical signs and characteristic feature of both parasitic and microbial infections.

The prevalence of IPs in Cameroon varied from 27.8% in 2012 [37], to 14.6% in 2013 [38] and 13.0% in this present study. Considering the HIV status, PLWH had a higher prevalence of IPs when compared with their HIV negative counterparts. This prevalence among PLWH is lower than the 59.52% [38] in 2013 and 57.48% [39] in 2014 reported among HIV patients in the Centre and West Regions of Cameroon respectively. This observed decrease in the prevalence of IPs among HIV patients may be accredited to the attainment of viral suppression by a majority (77.5%) as well as their better awareness of IPs and their causes. It may also be due to the improvement in the care provided to PLWH by the government of Cameroon by ensuring a constant supply and provision of ART free of charge.

This study revealed 3.7% of PLWH were co-infected with MP and IPs. Limited information is available on co-infection among PLWH in Cameroon; but when compared with other studies carried out among children [19, 40, 41], the prevalence is low. The mean age of PLWH, as well as the undetectable viral load observed in the majority of these patients, could account for this low prevalence. The lower the viral load, the faster the patient's immune system will recover, increasing the chances of fighting any infection easily. Besides, the majority of these patients have been on ART regularly for more than a year which probably could have contributed in boosting their immune systems against these parasites.

PLWH had a higher prevalence of anaemia when compared with their HIV negative counterparts. This prevalence is higher than the less than 56.0% reported in several studies carried out among PLWH [10, 11, 42-46,]. The reason for the observed difference might be due to the heterogeneity of the study population as the previously reported anaemia prevalence were those of children or

adult HIV patients, while this study included both children and adult participants (1–70 years old). Malaria and HIV positivity, as well as fever, were identified in this study as risk factors of anaemia and therefore contributed significantly to the high prevalence of anaemia. Mixed nutritional deficiencies (iron, folic acid, or vitamin B_{12}), though not investigated in this study, could have contributed to this high prevalence of anaemia as reported by a study carried out by Volberding et al. [47]. A direct comparison of the prevalence of anaemia in different studies is difficult as the study population, inclusion and exclusion criteria and anaemia definitions were different.

Parasites like the malaria parasites and intestinal parasites have long been recognized as major contributors to reduced haemoglobin levels in endemic countries like Cameroon, thereby causing anaemia. Also, PLWH on Zidovudine-containing ART have been reported to influence the haemoglobin level in previous studies [10, 11, 48-50]. In this study, patients infected with HIV, malaria and IPs had a significantly lower mean haemoglobin value (10.6 ± 1.21 g/dL). This is in line with previous studies which reported though among children, low haemoglobin values in patients with co-infections when compared with those with single infections [51-52]. Aside from well-reported side effects of anaemia associated with ZDV-containing ART regimen, though not confirmed in this study, anaemia among HIV infected individuals is more broadly multifactorial in origin, and three basic pathophysiologic mechanisms of anaemia in HIV infection have been postulated [47]: decreased RBC production, increased RBC destruction, and ineffective RBC production.

A high prevalence of severe anaemia was observed among patients co-infected with MP and HIV. This is consistent with studies carried out in Nigeria [53-54], Ethiopia [49] and more recently in

Cameroon [11]. This confirms the possibility that co-infections may increase malaria morbidity, further worsening malaria disease severity [55]. In addition, triple, dual, HIV and malaria parasite infections as well as fever were reported as risk factors of anaemia in this study.

One strength of this study is that it fills some gaps on the occurrence of MP/IPs co-infection among PLWH in the region which had not been reported previously as well as the association between these infections and anaemia among this vulnerable group. Further evidence is demonstrated on the outcome of the efforts in management implemented by the government which are encouraging in reducing the HIV/AIDS morbidity in the various communities. Notwithstanding, this study had as limitations the fact that nutritional deficiency as a cause of anaemia was not investigated, as well as the cross-sectional nature of the study that provides a picture of the health situation at a point in time. Nevertheless these findings are valuable to policymakers in guiding the management of these conditions. However, future longitudinal studies on the impact of iron supplementation and vitamin B_{12} levels among PLWH may be of great relevance to understand the cause of the high anaemia prevalence observed.

Conclusions

From this study, it can be concluded that malaria parasite and intestinal parasites remain a public health concern among PLWH with higher occurrences of both MP, IPs and MP/IP co-infections than in their HIV negative counterparts in the communities. Though a good majority of the HIV patients in the study had attained viral suppression, they are still more vulnerable to some of the parasites than HIV negative individuals and therefore can constitute a good source of contamination in their various communities. To achieve HIV epidemic control in 2021, the Cameroon Government needs to intensify the implementation of the various strategies. Anaemia

stands as a serious haematological abnormality among HIV patients and gets exacerbated even with the viral load suppression in the population of PLWH. Hence, there is a need for constant monitoring of the health status as well as the nutritional status of this at-risk group to better manage them.

Acknowledgements

The authors thank all the participants (both children and adults) who took part in the study as well as the staff at the Unit in-charge of patients with HIV (UPEC) in the Buea Regional Hospital. We are also thankful to the Buea community inhabitants who consented and parents who consented for their children to participate in the study.

Authors' Contributions

Conceptualization: Sorelle Mekachie Sandie.

Data curation: Irene Ule Ngole Sumbele.

Formal analysis: Sorelle Mekachie Sandie.

Investigation: Sorelle Mekachie Sandie, Irene Ule Ngole Sumbele, Martin Mih Tasah, Helen Kuokuo Kimbi.

Methodology: Sorelle Mekachie Sandie, Martin Mih Tasah.

Supervision: Irene Ule Ngole Sumbele, Helen Kuokuo Kimbi.

Validation: Irene Ule Ngole Sumbele, Helen Kuokuo Kimbi.

Writing – original draft: Sorelle Mekachie Sandie.

Writing – review & editing: Irene Ule Ngole Sumbele, Helen Kuokuo Kimbi.

References

1. Bangert M. The cross-cutting contribution of the end of neglected tropical diseases to the sustainable development goals. Infect Dis Poverty 2017; 6:73.

2. UNAIDS: UNAIDS report on the global AIDS epidemic 2018, Geneva, Switzerland.

3. Country Operational Plan (COP), Cameroon 2019. Strategic Direction Summary. https://www.state.gov/wp-content/uploads/2019/09/Cameroon_COP19-Strategic-Directional-Summary_public.pdf Accessed 07 Apr 2020.

4. Mabbott NA. The influence of parasite infections on host immunity to co-infection with other pathogens. Front Immunol. 2018. 8;9:2579.

5. World Health Organization. World Malaria Report 2019. Cameroon, African Region https://www.who.int/malaria/publications/country-profiles/profile_cmr_en.pdf?ua=1 2019. Accessed 28 Feb 2020.

6. World Health Organization. World Malaria Report 2018. Cameroon, African Region https://www.who.int/malaria/publications/country-profiles/profile_cmr_en.pdf?ua=1 2018. Accessed 04 Mar 2020.

7. Nkuo-Akenji T, Tevoufouet EE, Nzang F, Fon E, Ebong IN. HIV/AIDS and malaria in pregnant women from Cameroon short running title: HIV, malaria in pregnancy. Afr J Health Sci. 2011;18:105-109.

8. Njunda LA, Kamga HLF, Nsagha DS, Assoh JCN, Kwenti TE. Low malaria prevalence in HIV-positive patients in Bamenda, Cameroon. J Microbiol Res. 2012;2:56-59.

9. Kimbi HK, Njoh DT, Ndamukong KJN, Lehman LG. Malaria in HIV/AIDS patients at different CD4 T cell levels in Limbe, Cameroon. J Bacteriol Parasitol. 2013;4 (164). doi: 10.4172/2155-9597.1000164.

10. Bate B, Kimbi HK, Lum E, Lehman LG, Onyoh EF, Ndip LM, et al. Malaria infection and anaemia in HIV-infected children in Mutengene, Southwest Cameroon: a cross sectional study. BMC Infect Dis. 2016;16,523.

11. Sandie SM, Sumbele IUN, Tasah MM and Kimbi HK. Malaria parasite prevalence and Haematological parameters in HIV seropositive patients attending the regional hospital Limbe, Cameroon: a hospital-based cross-sectional study. BMC Infect Dis. 2019;19, 988.

12. Malaria Operational Plan (MOP) FY 2018 and FY 2019. President's Malaria Initiative Cameroon, 2019. https://www.pmi.gov/docs/default-source/default-document-library/malaria-operational-plans/fy-2018/fy-2018-cameroon-malaria-operational-plan.pdf?sfvrsn=5 Accessed: 07 Apr 2020.

13. Alemu G, Alelign D, Abossie A. Prevalence of opportunistic intestinal parasites and associated factors among HIV Patients while Receiving ART at Arba Minch Hospital in South Ethiopia: A Cross-sectional Study. Ethiop J Health Sci. 2018; 28(2):147.

14. Webb EL, Ekii AO, Pala P. Epidemiology and immunology of helminth = HIV interactions. Curr Opin HIV AIDS. 2012;7:245–253.

15. Adeleke OA, Yogeswaran P, Wright G. Intestinal helminth infections amongst HIV-infected adults in Mthatha General Hospital, South Africa. Afr J Prim Health Care Fam Med. 2015;7(1): 910.

16. Nsagha DS, Njunda AL, Assob NJC, Ayima CW, Tanue EA, kibu OD et al. Intestinal parasitic infections in relation to CD4+Tcell counts and diarrhoea in HIV/AIDS patients with or without antiretroviral therapy in Cameroon. BMC Infect Dis. 2016;16:9.

17. Nacher M, Singhasivanon P, Yimsamran S, Manibunyong W, Thanyavanich N, Wuthisen R. Intestinal helminth infections are associated with increased incidence of Plasmodium falciparum malaria in Thailand. J Parasitol. 2002;88(1):55–8.

18. Sokhna C, Le Hesran JY, Mbaye PA, Akiana J, Camara P, Diop M. Increased malaria attacks among children presenting concomitant infections by *S. mansoni* in Senegal. Malar J. 2004;3:43.

19. Njunda AL, Fon SG, Assob JCN, Nsagha DS, Kwenti TDB and Kwenti TE. Co-infection with malaria and intestinal parasites, and its association with anaemia in children in Cameroon. Infect Dis Poverty. 2015;4:43.

20. Wagnew F, Eshetie S, Alebel A, Tesema C, Kibret GD, Gebrie A, et al. Burden of anemia and its association with HAART in HIV infected children in Ethiopia: a systematic review and meta-analysis. BMC Infect Dis. 2019;19.

21. Brooker S, Akhwale W, Pullan R, Estambale B, Clarke SE, Snow RW. Epidemiology of *Plasmodium*-helminth co-infection in Africa: populations at risk, potential impact on anemia and prospects for combining control. Am J Trop Med Hyg.2007;77(6S):88–98.

22. Sumbele IU, Nkemnji GB. and Kimbi HK. Soil-transmitted helminths and plasmodium falciparum malaria among individuals living in different agroecosystems in two rural communities in the mount Cameroon area: a cross-sectional study. Infect Dis Poverty. 2017;6(1): 67.

23. Saracino A, Nacarapa EA, Massinga EAC, Martinelli D, Oliveira C, Antonich A, et al. Prevalence and clinical features of HIV and malaria co-infection in hospitalized adults in Beira, Mozambique. Malar J. 2012; 11:241.

24. Sumbele IUN, Sama SO, Kimbi HK and Taiwe GS. Malaria, moderate to severe anaemia, and malarial anaemia in children at presentation to hospital in the Mount Cameroon Area: a cross-sectional study. Anemia. 2016;Article ID 572.

25. CAMPHIA (Cameroon Population-based HIV Impact Assessment) 2017. Summary sheet: Preliminary findings. https://phia.icap.columbia.edu/wp-content/uploads/2018/07/3471CAMPHIA_Cameroon-SS_A4 v13_requests_7.25.18.pdf 2017. Accessed 09 Apr 2020.

26. World Health Organization. Malaria Factsheets 2020. https://www.who.int/news-room/fact-sheets/detail/malaria 2020. Accessed 02 Apr 2020.

27. Charan J and Biswas T. How to calculate sample size for different study designs in medical research? Indian J Psychol Med. 2013;35(2) 121-126.

28. Chanyalew MD and Gurara MK. Malaria and intestinal parasite infections and co-infections in Tach Gayint District, South Gondar Zone, Amhara Regional State 2010. Science J Public Health. 2014; 2(6): 546-553.

29. Cheesbrough M. District Laboratory Practice in Tropical Countries, 3Rd ed., part 1. United Kingdom: Cambridge University Press; 2014. p. 178–215.

30. UNAIDS. Undetectable = Untransmittable. Publich health and HIV load suppression. 2018.https://www.unaids.org/sites/default/files/media_asset/undetectable-untransmittable_en.pdf Accessed 09 Apr 2020.

31. WHO Nutritional anaemias: tools for effective prevention and control. Geneva, Switzerland: World Health Organization Publication; 2017. https://www.who.int/nutrition/publications/micronutrients/anaemias-tools-prevention-control/en/. Accessed 07 April 2020.

32. Minsante. XIième Journée mondiale de lutte contre le paludisme "prêt à vaincre le paludisme" Nous sommes la génération qui peut éliminer le paludisme. Dossier de Presse. Minsante. 2018:1–20.

33. Mbah-Mbole FG, Tufon KA, Meriki DH, Enow-Orock G, Mbah-Mbole P, Njunda LA, et al.. Malaria and human immunodeficiency virus coinfection in febrile patients attending the Regional Hospital of Buea, Southwest region, Cameroon. Int J Adv Med Health Res. 2018;6:46-51.

34. Sanyaolu AO, Fagbenro-Beyioku AF, Oyibo WA, Badaru OS, Onyeabor OS, Nnaemeka CI. Malaria and HIV co-infection and their effect on haemoglobin levels from three healthcare institutions in Lagos, southwest Nigeria. Afr Health Sci. 2013; 13(2):295-300.

35. Njunda AL, Njumkeng C, Nsagha SD, Assob JCN and Kwenti, TE. The prevalence of malaria in people living with HIV in Yaounde, Cameroon. BMC Public Health. 2016; 16:964.

36. Bouyou-Akotet M, Offouga C, Mawili-Mboumba D, Essola L, Madoungou B, Kombila M: Falciparum malaria as an emerging cause of fever in adults living in Gabon, Central Africa. BioMed Res Int. 2014; Article ID 351281.

37. Lehman LG, Kangam L, Nguepi E, Mbenoun ML and Bilong Bilong CF. Study of intestinal parasitic infections associated with HIV infection in Douala, Cameroon. Retrovirology. 2012;9 (Suppl 1): 48.

38. Nkenfou CN, Nana CT and Payne VK. Intestinal parasitic infections in HIV infected and non-infected patients in a low HIV prevalence region, West- Cameroon. PLoS ONE. 2013;8(2): e57914.

39. Vouking MZ, Enoka P, Tamo CV, Tadenfok CN. Prevalence of intestinal parasites among HIV patients at the Yaoundé Central Hospital, Cameroon. Pan African Med J. 2014;18:136.

40. Kwenti TE, Nkume FA, Tanjeko AT and Kwenti TDB. The effect of intestinal parasitic infection on the clinical outcome of malaria in coinfected children in Cameroon. PLoS Negl Trop Dis. 2016; 10(4): e0004673.

41. Angora KE, Djohan V, Konaté A, Kiki-Barro PC and Bedia-Tanoh AV. Uncomplicated malaria and intestinal helminth co-infections among schoolchildren in Abobo District, Abidjan (Côte d'Ivoire). J Infect Dis Epidemiol. 2019;5:064.

42. Assefa M, Abegaz WE and Mulugeta Belay. Prevalence and correlates of anemia among HIV infected patients on highly active anti-retroviral therapy at Zewditu Memorial Hospital, Ethiopia. BMC Hematol. 2015; 15: 6.

43. Ako SE, Njunda LA, Akum EA, Benjamin PT, Assob JC. Hematological related disorders and transfusion of HIV Patients on Highly Active Antiretroviral Therapy (HAART) in the South West Region of Cameroon: Hematological Monitory Parameters for HIV Follow-Up. J HIV Retrovirus.2018;4;1:5.

44. Lai JL, Chen YH, Liu YM, Yuan JJ, Lin J, Huang AQ et al. Prevalence and risk factors of anaemia in hospitalised HIV-infected patients in southeast China: a retrospective study. Epidemiol Infect 2019; 147:e81.

45. Gebremedhin KB and Haye TB. Factors associated with anemia among people living with HIV/AIDS taking art in Ethiopia. Hindawi Advances in Hematology. 2019; 8.

46. Ageru TA, Koyra MM, Gidebo KD, Abiso TL. Anemia and its associated factors among adult people living with human immunodeficiency virus at Wolaita Sodo University teaching referral hospital. PLoS ONE. 2019;14(10): 0221853.

47. Volberding PA, Levine AM, Dieterich D, Mildvan D, Mitsuyasu R, Saag M et al. Anemia in HIV Working Group. Anemia in HIV Infection: Clinical impact and evidence-based management strategies. Clin Infect Dis. 2004;38:1454–63.

48. Blake M and Sherer R. Management of the adverse effects of antiretroviral therapy and medication adherence. Clin Infect Dis. 2000;30(Suppl2):S96–116.

49. Ojurongbe O, Oyeniran O, Alli O, Taiwo S, Ojurongbe T, Olowe A, et al. Prevalence of *Plasmodium falciparum* parasitaemia and its correlation with haematological parameters among HIV-Positive individuals in Nigeria. J Trop Med Hyg. 2014:161284.

50. Breymann C. Iron deficiency anemia in pregnancy. Semin Hematol. 2015; 52(4):339–47.

51. Tay SC, Badu K, Mensah AA. and Gbedema SY. The prevalence of malaria among HIV seropositive individuals and the impact of the co- infection on their hemoglobin levels. Ann Clin Microbiol Antimicrob. 2015;14, 10.

52. Njua-Yafi C, Achidi EA, Anchang-Kimbi JK, Apinjoh TO, Mugri RN, Chi HF, et al. Malaria, helminths, co-infection and anaemia in a cohort of children from Mutengene, south western Cameroon. Malar J. 2016; 15:69.

53. Erhabor O, Babatunde S, and Uko KE. Some haematological parameters in Plasmodial parasitized HIV-infected Nigerians. Nigeria J Med. 2006; 15(1): 52-55.

54. Etusim PE, Ihemanma CA, Nduka FO, Melariri PE, Ukpai O. Comparative study on the haematological characteristics of malaria infected and malaria non-infected persons referred to art/HIV laboratory, Abia State University teaching hospital, aba, Abia state. Int J Sci Multidiscip Studies. 2013;5(1):100–12.

55. Rattanapunya S, Kuesap J, Chaijaroenkul W, Rueangweerayut R, and Na-Bangchang K. Prevalence of malaria and HIV coinfection and influence of HIV infection on malaria disease

severity in population residing in malaria endemic area along the Thai-Myanmar border. Acta Tropica. 2015:145:55.

Dear Editor

Many thanks for your email of 28th July 2020 providing reviewers' comments on our manuscript and inviting us to submit a revision. We have now prepared a revised manuscript reflecting the suggestions you made as well as those made by the reviewers for your consideration. Below we have provided point-by-point response to the reviewers' concerns and comments detailing the changes we have made in response to each point raised. The changes made to the manuscript have been highlighted in yellow and turquoise based on the comments and points raised by reviewers 1 and 2 respectively in the "Revised Manuscript with Track Changes". We would like to thank the reviewers for taking the time to read our manuscript and provide thoughtful and helpful comments for revision. We believe the revised manuscript represents a significant improvement of the original one.

We look forward to hearing from you in due course

Sorelle Sandie (on behalf of all authors)

See Authors' responses to reviewers' comments in the attached document.

Responses to Editor and Reviewers Comments and Concerns

We are submitting here responses to the concerns raised by reviewers on manuscript: PONE-D-20-20708.

Title:

Malaria and intestinal parasite co-infection and its association with anaemia among people living with HIV in Buea, Southwest Cameroon: A community-based cross-sectional study

Authors

Sorelle Mekachie Sandie*: sandiesorelle2@gmail.com

Irene Ule Ngole Sumbele: sumbelei@yahoo.co.uk

Martin Mih Tasah: tasahmartins@gmail.com

Helen Kuokuo Kimbi: hkimbi@yahoo.co.uk

Version: 2

Date: 26th August 2020

Authors' responses to reviewers' concerns over leaf.

Responses to Editor's Concerns

When submitting your revision, we need you to address these additional requirements.

1. Please ensure that your manuscript meets PLOS ONE's style requirements, including those for file naming. The PLOS ONE style templates can be found at

https://journals.plos.org/plosone/s/file?id=wjVg/PLOSOne_formatting_sample_main_body.pdf and

https://journals.plos.org/plosone/s/file?id=ba62/PLOSOne_formatting_sample_title_authors_affiliations.pdf

The manuscript has been corrected to meet PLOS ONE's style requirements as suggested by the editor.

2. Thank you for indicating in the text of your manuscript that you obtained written informed consent from participants. Please also add this information to your ethics statement in the online submission form.

Information on ethics statement has been included in the online submission form.

3. Please include additional information regarding the survey or questionnaire used in the study and ensure that you have provided sufficient details that others could replicate the analyses. For instance, if you developed a questionnaire as part of this study and it is not under a copyright more restrictive than CC-BY, please include a copy, in both the original language and English, as Supporting Information.

The questionnaire for this study was developed by the principal investigator. A copy has been uploaded as Supporting Information in the online submission.

Corrections and Concerns of Reviewer #1

The corrections proposed by reviewer #1 have been effected and are all highlighted in yellow in the revised manuscript.

Reviewer #1: The manuscript is well written but the authors need to provide clear description on laboratory methods. The results are well presented but table three need rearrangement to be clear to the reader. The conclusions are based on the study findings

Thanks for the comments. Laboratory methods, Table 3 in the results section as well as the conclusions as suggested by the reviewer have been upgraded and reorganized.

Abstract

Results

Line 37: But this is not statistically significant

Effectively, the difference was not statistically significant across the two groups; but as malaria parasite prevalence was one of the main outcomes in the study, it could not be left out in the abstract because of that. Apart from statistical significance, it is also important to know the trend of the various infections across the groups.

Methods

Study design and sample size

Line 138: This sample size was not intended/powered to compare two proportions

You raised a pertinent issue concerning sample size calculation. To address that, the following sample size formula was used as we are comparing two proportions (See lines 138-143).

$N=2(Z_{\alpha} + Z_{\beta})^2 P(1-P)/(P_0-P_1)^2$

Where;

N=sample size

Z_{α}= 1.96

Z_{β}= 0.84

P = Pooled prevalence = $(P_0 + P_1)/2$

P_0 = 50% (unknown prevalence)

P_1 = 67.2% (Chanyalew and Gurara, 2014)

The calculated sample size (N) was 128 for each group.

Sample collection and processing

Line 164: Briefly describe how you quantified the parasites

A statement on quantification of parasites has been included in the revised manuscript (See lines 164 – 166).

Line 167: Provide details how quantification was done and quality assurance of the readings

A statement on malaria parasite quantification has been included in the revised manuscript (See lines 166 – 172).

The authors omitted to mention that blood smears were prepared and read by 2 qualified microscopists, and that a third microscopist was requested in case of discrepancy in some results; although that was effectively done. This information has been included under sample collection and processing (See lines 166 -169).

For quality assurance, a negative result was declared after checking at least 200 high power microscopic fields.

Line 185: Please provide reference

On line 185, the authors just stated the apparatus used for haemoglobin measurement; giving details on the location of the manufacturing company. The authors do not understand which reference should be added on that statement.

Table 3

Line 276: It will be easier to interpret this table if rearranged into two categories HIV+ve and HIV –ve like table2, then see how anemia and co-infection vary across groups.

In Table 3, the authors wanted to bring out clearly the difference in the prevalence of anaemia and its severity with respect to the various categories of infections (single, double and triple infections); so if arranged into two categories, that difference will not be clearly seen.

Figure 1: Please indicate that these participants were excluded/not enrolled.

A statement on that has been included under the description of socio-demographic data and referred to Figure 1 (See lines 216 – 218).

Corrections and Concerns of Reviewer #2

The corrections proposed by reviewer #2 (Rodolfo Rossi) have been effected and are all highlighted in turquoise in the revised manuscript.

Reviewer #2: Thank you for giving me the opportunity to review this manuscript, which is rather well written and clear. The manuscript adds on the evidence of various conditions common is sub-Saharan Africa and links them to the HIV pandemic. However, in spite of its merits, the manuscript should undergo some review prior potential publication.

Both major and minor comments are explained below in order of the page of the manuscript:

Page 3 – Lines 52-55: the authors start the manuscript by trying to give evidence of what is known on the issue, which is good. However, this part could be expanded; there is literature that the authors can consider to make their statements stronger (as an example: https://doi.org/10.1016/j.gore.2019.07.002). Therefore, I suggest an additional search in the literature.

Thank you for the suggestion, which has been taken into consideration by the authors. After literature search, the background has been expanded to make the statements stronger (See lines 54 – 62).

Page 6 – Lines 126-127: It is not clear how the participants were selected, specifically in terms of their HIV status. Were 2 different selections done on the basis of HIV status, meaning, were the authors specifically going to look for HIV+ and HIV- participants? If so, this study should be redesigned as a (retrospective) cohort since participants are selected on the base of an exposure (i.e. HIV) and see the differences in outcomes (i.e. anaemia, IP, etc.). In cross-sectional studies, a sample of the population is selected, not necessarily on the base of some individual variables. Please, clarify this point and rectify it all along the manuscript.

The authors believe this study is a cross-sectional study because the participants were recruited from the communities not based on their exposure as well as from the HIV treatment centre in the community to make up the numbers of HIV positive cases. Hence, we had both exposed (HIV patients who already knew their status) and unexposed participants (HIV negatives who had no

knowledge of their status and were tested to confirm their HIV status) recruited from both the community and HIV treatment centre. Those who were tested positive for HIV in the community outreach were included in the HIV positive group.

Page 9 – Lines 194-196: the authors stated that they conducted a binary logistic regression, which appear somehow in contradiction with what written on line 281 ("A multinomial regression analysis was used…"). In logistic regression, usually we talk about "crude" (or univariate) analysis if there is only one exposure and the outcome in the model, or "adjusted" (or multivariable) analysis if there are more than one exposure in the same model. Please, specify which one you used, and if it is crude, please explain (with good reason) why an adjusted analysis was not conducted (which should be). In addition, please, describe which statistical test was used in the logistic regression (which can either be the Wald test or the Likelihood Ratio test).

The authors noticed the contradiction raised by the reviewer concerning the analysis that was used to determine odds ratio and it has been addressed in the revised manuscript.

To address the concern regarding odd ratios, the data was reanalyzed and both the crude and adjusted odds ratios were included in table 4 in the revised manuscript.

The statistical test used was the Likelihood Ratio test.

Page 10-11 (Table 1) and page 12-13 (Table 2): you provide descriptive statistics for various variables of participants. Please, provide the p-value from the Chi2 test to see if there are statistically significant differences in the various variables between HIV+ and HIV- participants.

As seen in Tables 1 and 2, the P- and Chi-square values have been included to show whether there were statistical significance differences in the various variables between HIV positive and negative participants.

Page 16 – Table 4: Please, again explain whether the OR are crude or adjusted (see point above); please, include (for clarity) what is the reference for each variable (e.g. if males have a OR of 0.37 for anaemia, it is in comparison to women, which will be the reference) in the table, rather than in the table legenda; please, also include which statistical test was used (see above).

The reference for each variable has been included in table 4 for better understanding of the results (See line 308).

Page 17 onwards (all Discussion section): please reorganize the whole Discussion/Conclusion section. This should be according to a logical sequence. Start by summarizing the findings of your study (no need to repeat values such as percentages and means in the discussion section), how they compare with other similar studies, what are the reasons for differences with other studies. Provide a plausibility for your findings. Provide Strengths and Limitations of your study (this is essential to see to what extent you can give recommendations following your results). Once you have gone through the strengths and limitations of your study, you should provide which are the implications of your results, these may be clinical, public health/programmatic, advocacy etc. as recommendations plus all what you consider to be done as additional research on the topic.
The discussion section has been improved as requested by the reviewer (See lines 423-433).
