# Supplementary material for: Malaria and intestinal parasite co-infection and its association with anaemia among people living with HIV in Buea, Southwest Cameroon: A community-based retrospective cohort study
Source: PLoS One. 2021 Jan 22;16(1):e0245743. doi: 10.1371/journal.pone.0245743 (PMC7822292; doi:10.1371/journal.pone.0245743)
Supplement: Attachment — Submitted filename: Response to Reviewers.docx [file pone.0245743.s003.docx]

**Dear Editor**

Many thanks for your email of 28^th^ July 2020 providing reviewers’ comments on our manuscript and inviting us to submit a revision. We have now prepared a revised manuscript reflecting the suggestions you made as well as those made by the reviewers for your consideration. Below we have provided point-by-point response to the reviewers’ concerns and comments detailing the changes we have made in response to each point raised. The changes made to the manuscript have been highlighted in yellow and turquoise based on the comments and points raised by reviewers 1 and 2 respectively in the “Revised Manuscript with Track Changes”. We would like to thank the reviewers for taking the time to read our manuscript and provide thoughtful and helpful comments for revision. We believe the revised manuscript represents a significant improvement of the original one.

We look forward to hearing from you in due course

Sorelle Sandie (on behalf of all authors)

See Authors’ responses to reviewers’ comments in the attached document.

**Responses to Editor and Reviewers Comments and Concerns**

We are submitting here responses to the concerns raised by reviewers on manuscript: PONE-D-20-20708.

**Title**:

**Malaria and intestinal parasite co-infection and its association with anaemia among people living with HIV in Buea, Southwest Cameroon: A community-based cross-sectional study**

**Authors**

Sorelle Mekachie Sandie*: [sandiesorelle2@gmail.com](mailto:sandiesorelle2@gmail.com)

Irene Ule Ngole Sumbele: [sumbelei@yahoo.co.uk](mailto:sumbelei@yahoo.co.uk)

Martin Mih Tasah: [tasahmartins@gmail.com](mailto:tasahmartins@gmail.com)

Helen Kuokuo Kimbi: [hkimbi@yahoo.co.uk](mailto:hkimbi@yahoo.co.uk)

Version: 2

Date: 26^th^ August 2020

Authors’ responses to reviewers’ concerns over leaf.

**Responses to Editor’s Concerns**

When submitting your revision, we need you to address these additional requirements.

**1. Please ensure that your manuscript meets PLOS ONE's style requirements, including those for file naming. The PLOS ONE style templates can be found at**

<https://journals.plos.org/plosone/s/file?id=wjVg/PLOSOne_formatting_sample_main_body.pdf> and

<https://journals.plos.org/plosone/s/file?id=ba62/PLOSOne_formatting_sample_title_authors_affiliations.pdf>

The manuscript has been corrected to meet PLOS ONE’s style requirements as suggested by the editor.

**2. Thank you for indicating in the text of your manuscript that you obtained written informed consent from participants. Please also add this information to your ethics statement in the online submission form.**

 Information on ethics statement has been included in the online submission form.

**3. Please include additional information regarding the survey or questionnaire used in the study and ensure that you have provided sufficient details that others could replicate the analyses. For instance, if you developed a questionnaire as part of this study and it is not under a copyright more restrictive than CC-BY, please include a copy, in both the original language and English, as Supporting Information.**

The questionnaire for this study was developed by the principal investigator. A copy has been uploaded as Supporting Information in the online submission.

**Corrections and Concerns of Reviewer #1**

The corrections proposed by reviewer #1 have been effected and are all highlighted in yellow in the revised manuscript.

**Reviewer #1: The manuscript is well written but the authors need to provide clear description on laboratory methods. The results are well presented but table three need rearrangement to be clear to the reader. The conclusions are based on the study findings**

Thanks for the comments. Laboratory methods, Table 3 in the results section as well as the conclusions as suggested by the reviewer have been upgraded and reorganized.

**Abstract**

**Results**

**Line 37: But this is not statistically significant**

Effectively, the difference was not statistically significant across the two groups; but as malaria parasite prevalence was one of the main outcomes in the study, it could not be left out in the abstract because of that. Apart from statistical significance, it is also important to know the trend of the various infections across the groups.

**Methods**

**Study design and sample size**

**Line 138: This sample size was not intended/powered to compare two proportions**

You raised a pertinent issue concerning sample size calculation. To address that, the following sample size formula was used as we are comparing two proportions (See lines 138-143).

N=2(Z_α_ + Z_β_)^2^ P(1-P)/(P_0_-P_1_)^2^

Where;

N=sample size

Z_α_= 1.96

Z_β_= 0.84

P = Pooled prevalence = (P_0_ + P_1_)/2

P_0_ = 50% (unknown prevalence)

P_1_ = 67.2% (Chanyalew and Gurara, 2014)

The calculated sample size (N) was 128 for each group.

**Sample collection and processing**

**Line 164: Briefly describe how you quantified the parasites**

A statement on quantification of parasites has been included in the revised manuscript (See lines 164 – 166).

**Line 167: Provide details how quantification was done and quality assurance of the readings**

A statement on malaria parasite quantification has been included in the revised manuscript (See lines 166 – 172).

The authors omitted to mention that blood smears were prepared and read by 2 qualified microscopists, and that a third microscopist was requested in case of discrepancy in some results; although that was effectively done. This information has been included under sample collection and processing (See lines 166 -169).

For quality assurance, a negative result was declared after checking at least 200 high power microscopic fields.

**Line 185: Please provide reference**

On line 185, the authors just stated the apparatus used for haemoglobin measurement; giving details on the location of the manufacturing company. The authors do not understand which reference should be added on that statement.

**Table 3**

**Line 276: It will be easier to interpret this table if rearranged into two categories HIV+ve and HIV –ve like table2, then see how anemia and co-infection vary across groups.**

In Table 3, the authors wanted to bring out clearly the difference in the prevalence of anaemia and its severity with respect to the various categories of infections (single, double and triple infections); so if arranged into two categories, that difference will not be clearly seen.

**Figure 1: Please indicate that these participants were excluded/not enrolled.**

A statement on that has been included under the description of socio-demographic data and referred to Figure 1 (See lines 216 – 218).

**Corrections and Concerns of Reviewer #2**

The corrections proposed by reviewer #2 (Rodolfo Rossi) have been effected and are all highlighted in turquoise in the revised manuscript.

Reviewer #2: Thank you for giving me the opportunity to review this manuscript, which is rather well written and clear. The manuscript adds on the evidence of various conditions common is sub-Saharan Africa and links them to the HIV pandemic.
However, in spite of its merits, the manuscript should undergo some review prior potential publication.
Both major and minor comments are explained below in order of the page of the manuscript:

**Page 3 – Lines 52-55: the authors start the manuscript by trying to give evidence of what is known on the issue, which is good. However, this part could be expanded; there is literature that the authors can consider to make their statements stronger (as an example:** [**https://doi.org/10.1016/j.gore.2019.07.002**](https://doi.org/10.1016/j.gore.2019.07.002) **). Therefore, I suggest an additional search in the literature.**

Thank you for the suggestion, which has been taken into consideration by the authors. After literature search, the background has been expanded to make the statements stronger (See lines 54 – 62).

**Page 6 – Lines 126-127: It is not clear how the participants were selected, specifically in terms of their HIV status. Were 2 different selections done on the basis of HIV status, meaning, were the authors specifically going to look for HIV+ and HIV- participants? If so, this study should be redesigned as a (retrospective) cohort since participants are selected on the base of an exposure (i.e. HIV) and see the differences in outcomes (i.e. anaemia, IP, etc.). In cross-sectional studies, a sample of the population is selected, not necessarily on the base of some individual variables. Please, clarify this point and rectify it all along the manuscript.**

The authors believe this study is a cross-sectional study because the participants were recruited from the communities not based on their exposure as well as from the HIV treatment centre in the community to make up the numbers of HIV positive cases. Hence, we had both exposed (HIV patients who already knew their status) and unexposed participants (HIV negatives who had no knowledge of their status and were tested to confirm their HIV status) recruited from both the community and HIV treatment centre. Those who were tested positive for HIV in the community outreach were included in the HIV positive group.

**Page 9 – Lines 194-196: the authors stated that they conducted a binary logistic regression, which appear somehow in contradiction with what written on line 281 (“A multinomial regression analysis was used…”). In logistic regression, usually we talk about “crude” (or univariate) analysis if there is only one exposure and the outcome in the model, or “adjusted” (or multivariable) analysis if there are more than one exposure in the same model. Please, specify which one you used, and if it is crude, please explain (with good reason) why an adjusted analysis was not conducted (which should be). In addition, please, describe which statistical test was used in the logistic regression (which can either be the Wald test or the Likelihood Ratio test).**

The authors noticed the contradiction raised by the reviewer concerning the analysis that was used to determine odds ratio and it has been addressed in the revised manuscript.

To address the concern regarding odd ratios, the data was reanalyzed and both the crude and adjusted odds ratios were included in table 4 in the revised manuscript.

The statistical test used was the Likelihood Ratio test.

**Page 10-11 (Table 1) and page 12-13 (Table 2): you provide descriptive statistics for various variables of participants. Please, provide the p-value from the Chi2 test to see if there are statistically significant differences in the various variables between HIV+ and HIV- participants**.
As seen in Tables 1 and 2, the P- and Chi-square values have been included to show whether there were statistical significance differences in the various variables between HIV positive and negative participants.

**Page 16 – Table 4: Please, again explain whether the OR are crude or adjusted (see point above); please, include (for clarity) what is the reference for each variable (e.g. if males have a OR of 0.37 for anaemia, it is in comparison to women, which will be the reference) in the table, rather than in the table legenda; please, also include which statistical test was used (see above).**

The reference for each variable has been included in table 4 for better understanding of the results (See line 308).

**Page 17 onwards (all Discussion section): please reorganize the whole Discussion/Conclusion section. This should be according to a logical sequence. Start by summarizing the findings of your study (no need to repeat values such as percentages and means in the discussion section), how they compare with other similar studies, what are the reasons for differences with other studies. Provide a plausibility for your findings. Provide Strengths and Limitations of your study (this is essential to see to what extent you can give recommendations following your results). Once you have gone through the strengths and limitations of your study, you should provide which are the implications of your results, these may be clinical, public health/programmatic, advocacy etc. as recommendations plus all what you consider to be done as additional research on the topic.**
The discussion section has been improved as requested by the reviewer (See lines 423-433).
