# Supplementary material for: Malaria and intestinal parasite co-infection and its association with anaemia among people living with HIV in Buea, Southwest Cameroon: A community-based retrospective cohort study
Source: PLoS One. 2021 Jan 22;16(1):e0245743. doi: 10.1371/journal.pone.0245743 (PMC7822292; doi:10.1371/journal.pone.0245743)
Supplement: Attachment — Submitted filename: Reviewers Response.docx [file pone.0245743.s005.docx]

**Dear Editor**

Many thanks for your email of 29^th^ September 2020 providing reviewers’ comments on our manuscript and inviting us to submit a revision. We have now prepared a revised manuscript reflecting the suggestions you made as well as those made by the reviewers for your consideration. Below we have provided point-by-point response to the reviewers’ concerns and comments detailing the changes we have made in response to each point raised. The changes made to the manuscript have been highlighted in yellow and turquoise based on the comments and points raised by reviewers 1 and 2 respectively in the “Revised Manuscript with Track Changes”. We would like to thank the reviewers for taking the time to read our manuscript and provide thoughtful and helpful comments for revision. We believe the revised manuscript represents a significant improvement of the original one.

We look forward to hearing from you in due course

Sorelle Sandie (on behalf of all authors)

See Authors’ responses to reviewers’ comments in the attached document.

**Responses to Reviewers Comments and Concerns**

We are submitting here responses to the concerns raised by reviewers on manuscript: PONE-D-20-20708.

**Title**:

**Malaria and intestinal parasite co-infection and its association with anaemia among people living with HIV in Buea, Southwest Cameroon: A community-based cross-sectional study**

**Authors**

Sorelle Mekachie Sandie*: [sandiesorelle2@gmail.com](mailto:sandiesorelle2@gmail.com)

Irene Ule Ngole Sumbele: [sumbelei@yahoo.co.uk](mailto:sumbelei@yahoo.co.uk)

Martin Mih Tasah: [tasahmartins@gmail.com](mailto:tasahmartins@gmail.com)

Helen Kuokuo Kimbi: [hkimbi@yahoo.co.uk](mailto:hkimbi@yahoo.co.uk)

Version: 3

Date: 17^th^ October 2020

Authors’ responses to reviewers’ concerns over leaf.

**Corrections and Concerns of Reviewer #1**

The corrections proposed by reviewer #1 have been effected and are all highlighted in yellow in the revised manuscript.

**Is in the study design (cross sectional study) but sampling of participants in the clinics (PLWH) and community (HIV -ve participants) is not well described.**

The sampling of participants for both categories has been described in the study design section as suggested (See lines 137 – 144).

**In the analysis the authors are not only comparing the two groups PLWH and HIV -ve they also add other sub groups which makes to interpret the findings. Throughout the text they use either PLHW or HIV +ve to refer to the same group of participants**

In this study, PLWH and HIV positive patients referred to the same group of participants. For better clarification and interpretation, the term “PLWH” has been used throughout the manuscript instead of HIV positive patients.

**Abstract**

**Results**

**Line 35: Here you could have a sentence to show the prevalence results; PLWH vs HIV –ve instead of overall prevalence in the first sentence.**

The authors are grateful for the suggestion. However, the authors believe it is most appropriate to give the prevalence of the different pathogens under study before stating the prevalence in association with different conditions examined in the study for a logical presentation. This presentation ties with the title of the manuscript. (See lines 35-39).

**Line 38: Use the same term PLWH and show mean Hb for the 2 groups.**

The term PLWH has been used in Line 38 and the mean Hb for the 2 groups has been reported (See lines 38-39).

**Line 40: This was not the focus of your study.**

The statement of line 40 has been removed as it was not the focus of the study.

**Line 41: Use PLWH instead of HIV positive patients.**

The term “HIV positive patients” has been replaced by “PLWH” throughout the manuscript.

**Methods**

**Study design and sample size**

**Line 137: This study also collected data from clinics as stated above in the description of study area.**

Yes. That has been clearly stated in lines 140-143 and lines 153-155. PLWH recruited from the HIV Treatment and Care centre of the hospital had data from the questionnaire verified with that in their file in the centre. In addition, the appropriate laboratory data measured on the day of recruitment was also obtained from the treatment centre. Hence, the description of data collection at the level of the HIV Treatment and Care Centre has been included in this section as mentioned in the lines above.

**Line 138: It is not clearly stated how the HIV positive individuals were sampled in the community or health facility.**

A more comprehensive statement on how HIV positive patients were recruited in this study can be found in the study design section (See lines 141-145).

**Line 139: Why convenient sampling?**

The sampling method used was a purposive convenient sampling method. Purposive because the study was meant to compare two groups of participants and convenient because the participants for the study could be easily enrolled from this cohort of individuals. It was quite convenient for them to be enrolled as they presented themselves and gave their consents (See line 137).

**Table 2**

**Line 252: How many HIV positive patients were screened in the community?**

From the outreach screening programme in the community, four participants were found living with HIV that were referred to the HIV-care and treatment centre for their enrolment follow-up and collection of appropriate data.

**Malaria parasite, Intestinal parasite infections and malaria/intestinal parasite co-infection**

**Line 257: Decide which term to use PLWH or HIV positive participants.**

PLWH has been used throughout the manuscript.

**Line 258: Recalculate this is around 16.6 65/406.**

Effectively there was a problem with the calculation of the prevalence of IPs in the total population. PLWH infected with IPs were 45 in number giving a prevalence of 23.7% while HIV negative participants infected with IPs were 12 in number giving a prevalence of 5.6%. The total number of participants infected with IPs irrespective of their HIV status was supposed to be 45 + 12 = 57, giving a total prevalence of 14.0%. The correction has been effected in the manuscript (See line 259-260) as well as in Figure 2.

**Line 263: This sentence should be rephrased, although not statistically significant, malaria/IP co-infection was higher in PLWH compared to HIV –ve participants.**

The sentence on line 263 has been rephrased appropriately.

**Corrections and Concerns of Reviewer #2**

The corrections proposed by reviewer #2 (Rodolfo Rossi) have been effected and are all highlighted in turquoise in the revised manuscript.

Thank you for addressing the vast majority of the comments I had made during the first revision.

There are still 2 pending issues that could be better addressed

**Lines 130 – 131: "The two categories of participants, PLWH and HIV-negative individuals were enrolled in the study at the HIV Treatment and Care Centre and from the different neighbourhoods". By reading this, I still understand that participants were recruited according to their HIV status. Therefore I kindly ask you to reformulate since you state that your study is a cross-sectional study**

The authors believe this study is not a retrospective cohort study because both the exposure (HIV status) and outcome (Malaria, anaemia, IPs) were measured at the same time, making it a cross-sectional study. Hence, participants were recruited in a cross-sectional study and grouped according to their status into PLWH and HIV- negative participants. The statement has been reformulated as suggested by the reviewer for better understanding of the study design (See lines 129-130)

**Discussion**

**In the discussion section, when summarizing the results, I suggest removing all/most numerical values since these are already reported in the results section.**

As suggested by the reviewer, most numerical values have been removed from the discussion section.
