# Supplementary material for: Malaria and intestinal parasite co-infection and its association with anaemia among people living with HIV in Buea, Southwest Cameroon: A community-based retrospective cohort study
Source: PLoS One. 2021 Jan 22;16(1):e0245743. doi: 10.1371/journal.pone.0245743 (PMC7822292; doi:10.1371/journal.pone.0245743)
Supplement: Attachment — Submitted filename: Response to Reviewers.docx [file pone.0245743.s006.docx]

**Dear Editor**

Many thanks for your email of 26^th^ October 2020 providing reviewers’ comments on our manuscript and inviting us to submit a revision. We have now prepared a revised manuscript reflecting the suggestions you made as well as those made by the reviewers for your consideration. Below we have provided point-by-point response to the reviewers’ concerns and comments detailing the changes we have made in response to each point raised. The changes made to the manuscript have been highlighted in yellow and turquoise based on the comments and points raised by reviewers 1 and 2 respectively in the “Revised Manuscript with Track Changes”. We would like to thank the reviewers for taking the time to read our manuscript and provide thoughtful and helpful comments for revision. We believe the revised manuscript represents a significant improvement of the original one.

We look forward to hearing from you in due course

Sorelle Sandie (on behalf of all authors)

See Authors’ responses to reviewers’ comments in the attached document.

**Responses to Reviewers Comments and Concerns**

We are submitting here responses to the concerns raised by reviewers on manuscript: PONE-D-20-20708.

**Title**:

**Malaria and intestinal parasite co-infection and its association with anaemia among people living with HIV in Buea, Southwest Cameroon: A community-based retrospective cohort study**

**Authors**

Sorelle Mekachie Sandie*: [sandiesorelle2@gmail.com](mailto:sandiesorelle2@gmail.com)

Irene Ule Ngole Sumbele: [sumbelei@yahoo.co.uk](mailto:sumbelei@yahoo.co.uk)

Martin Mih Tasah: [tasahmartins@gmail.com](mailto:tasahmartins@gmail.com)

Helen Kuokuo Kimbi: [hkimbi@yahoo.co.uk](mailto:hkimbi@yahoo.co.uk)

Version: 4

Date: 28^th^ October 2020

Authors’ responses to reviewers’ concerns over leaf.

**Corrections and Concerns of Editor**

**According to the reviewer, the concerns raised during the peer review process were not properly addressed. For example, reviewer #2 complains that the study design is still confuse. According to the reviewer, people HIV+/HIV- were categorized by exposure at the beginning of the study, therefore the study, as presented, is not a cross-sectional study. At this time we strongly suggest the authors to proper address the concerns related to study design.**

After thorough literature search on research methodology for better understanding, the authors have acknowledged the concerns of the second reviewer on the study design and have addressed them appropriately throughout the manuscript.

The study design has been modified to a retrospective cohort study design as the exposure (i.e HIV status) was known at the start of the study; making it not a cross-sectional study.

**Corrections and Concerns of Reviewer #1**

The corrections proposed by reviewer #1 have been effected and are all highlighted in yellow in the revised manuscript.

**The conclusion in the abstract should be similar to conclusion at the end of discussion section. ie focusing on burden of infections MP, IPs and Co infection MP/IPs . Currently Conclusion: it is stated Multiple infections exacerbate the health condition of PLWH.**

The concern raised concerning the abstract’s conclusion has been addressed appropriately as suggested by the reviewer (See line 46).

**Corrections and Concerns of Reviewer #2**

The corrections proposed by reviewer #2 (Rodolfo Rossi) have been effected and are all highlighted in turquoise in the revised manuscript.

**Thank you for your efforts, but the issue of study design highlighted by me, the other reviewer and the editor in the previous round of review has not been properly addressed, leaving confusion on the understanding of study design. Even if recruited simultaneously, people HIV+ and HIV- are categorized by exposure at the beginning of the study, therefore the study, as presented is not a cross-sectional study.**

After thorough literature search on research methodology for better understanding, the authors have acknowledged the concerns on the study design and have addressed them appropriately throughout the manuscript.

The study design has been modified to a retrospective cohort study design as the exposure (i.e HIV status) was known at the start of the study; not making it a cross-sectional study.
