# Supplementary material for: Malaria and intestinal parasite co-infection and its association with anaemia among people living with HIV in Buea, Southwest Cameroon: A community-based retrospective cohort study
Source: PLoS One. 2021 Jan 22;16(1):e0245743. doi: 10.1371/journal.pone.0245743 (PMC7822292; doi:10.1371/journal.pone.0245743)
Supplement: Attachment — Submitted filename: Response to Reviewers.docx [file pone.0245743.s007.docx]

**Dear Editor**

Many thanks for your email of 17^th^ November 2020 providing reviewers’ comments on our manuscript and inviting us to submit a revision. We have now prepared a revised manuscript reflecting the suggestions made by the reviewer for your consideration. Below we have provided point-by-point response to the reviewer’s concerns and comments detailing the changes we have made in response to each point raised. The changes made to the manuscript have been highlighted in yellow based on the comments and points raised by the reviewer in the “Revised Manuscript with Track Changes”. We would like to thank the reviewer for taking the time to read our manuscript and provide thoughtful and helpful comments for revision. We believe the revised manuscript represents a significant improvement of the original one.

We look forward to hearing from you in due course

Sorelle Sandie (on behalf of all authors)

See Authors’ responses to reviewer’s comments in the attached document.

**Responses to Reviewer’s Comments and Concerns**

We are submitting here responses to the concerns raised by the reviewer on manuscript: PONE-D-20-20708.

**Title**:

**Malaria and intestinal parasite co-infection and its association with anaemia among people living with HIV in Buea, Southwest Cameroon: A community-based retrospective cohort study**

**Authors**

Sorelle Mekachie Sandie*: [sandiesorelle2@gmail.com](mailto:sandiesorelle2@gmail.com)

Irene Ule Ngole Sumbele: [sumbelei@yahoo.co.uk](mailto:sumbelei@yahoo.co.uk)

Martin Mih Tasah: [tasahmartins@gmail.com](mailto:tasahmartins@gmail.com)

Helen Kuokuo Kimbi: [hkimbi@yahoo.co.uk](mailto:hkimbi@yahoo.co.uk)

Version: 5

Date: 20^th^ November 2020

Authors’ responses to reviewer’ concerns over leaf.

**Corrections and concerns of Reviewer #3:**

**Pg 3, lines 54-55: “In 2018, there were... “. What is the current status for new cases (in 2020)? Is there any improvement? Please include this with citations. Also include the current status in Cameroun (line 56).**

A more current status of HIV new cases has been given as reported by UNAIDS in 2019 and that report carries information related to the year 2018. That is why the authors specified that this data was for the year 2018; though it was reported by UNAIDS in 2019. With regards to Cameroon, the most current report is that of 2018 as of now as reported by UNAIDS.

**Pg 3, line 65: “variety of microbial and parasitic infections such as Plasmodium, Mycobacterium tuberculosis... “. I suggest that authors write the infections (malaria, tuberculosis) instead of the microbes, to eliminate any possible ambiguity.**

The sentence has been adjusted to eliminate ambiguity (See line 65). Malaria is the disease that results from an infection with *Plasmodium* likewise tuberculosis arises because of an infection with *M. tuberculosis*. Hence, it is necessary to state the infectious agent.

**Pg 4, 72-73: “African countries still bear the largest burden... “. 2018 is long time to make this conclusive statement. The situation may have changed within 2 years. I suggest authors provide a recent data/citation to back up this statement.**

The most recent published malaria report by WHO is that of 2019, which reports the burden of malaria in 2018. Malaria burden for 2019 has not yet been published by WHO.

**Pg 6, lines 123-125: “ In the national HIV prevalence ranking... “. The reference (25) show that this impact assessment was done in 2017, hence the South West Region may no longer be the 6th on the ranking in 2020. I suggest that authors obtain more recent data OR rephrase the statement to indicate that information presented is not recent, to avoid misleading prospective readers.**

The data presented is the most recent published information on HIV in Cameroon. The authors believe that as long as there are no updated reports on the ranking, the South West Region still remains in the 6^th^ position on the ranking.

**Pg 6, 129-130: There is also inadequate access to safe water... “. Please provide a reference for this.**

Reference 27 has been added to support the statement and the sentence has been rephrased appropriately to avoid misunderstanding (See line 131 – 133).

**Study area and participants: age range of participants was not specified.**

As suggested by the reviewer, age range has been added to the statement (See line 135).

**Pg 6, 133: “after confirmation of their HIV status ...”. Please specify the steps/assays used for the confirmation of HIV Status.**

The test used for HIV confirmation was Determine Test strip and this was specified in the statement (See line 136).

**Pg 7, 143-148: “HIV-negative participants included persons who ... “. This section is describing the study participants. Please move to appropriate section.**

The section describing the study participants has been moved to the appropriate section as suggested by the reviewer (See lines 136 – 141).

**Pg 11, 238 “were students (56.5%)...”. I presume authors are referring to the employment status of this category? If so, I suggest the use of ”unemployed” instead if this is the case, as some students engage in work study**.

In our setting in Cameroon, most students are not engaged in work activities and that is why this terms was used for those participants who are students and do not have any lucrative activity. There is a section under employment status for those who are not students and are unemployed and that category is referred to as “unemployment”..

**Tables 1 and 2 have grid lines while tables 3 and 4 do not. I suggest a uniformity in table presentation according to journal’s guidelines.**

Grid lines have been added to tables 3 and 4 to follow the journal’s guidelines for presentation of tables.

**Pg 15: 273-276: “Out of the 406 participants, 1.7% (7) had triple infection...”. Are the authors describing those with triple infection etc from the population or individuals with triple infection etc who were anaemic? If it is haemoglobin level that is being described, anaemia was not indicated anywhere in this section aside from the subtitle. Authors should endeavour to clarify this section.**

Those with triple infection are those who were found to be infected with HIV, malaria parasite and intestinal parasites simultaneously.

Under the section “Infection categories, haemoglobin level and anaemia prevalence”, the first part describes the different infection categories followed by their effects on the haemoglobin level. Then, after figure 3 which displays the differences in mean haemoglobin level with respect to infection categories, anaemia prevalence is discussed and it still falls under the same section.
