# Supplementary material for: Malaria and intestinal parasite co-infection and its association with anaemia among people living with HIV in Buea, Southwest Cameroon: A community-based retrospective cohort study
Source: PLoS One. 2021 Jan 22;16(1):e0245743. doi: 10.1371/journal.pone.0245743 (PMC7822292; doi:10.1371/journal.pone.0245743)
Supplement: Attachment — Submitted filename: Response to Reviewers.docx [file pone.0245743.s008.docx]

**Dear Editor**

Many thanks for your email of 24^th^ November 2020 providing reviewers’ comments on our manuscript and inviting us to submit a revision. We have now prepared a revised manuscript reflecting the suggestions made by the reviewer for your consideration. Below we have provided point-by-point response to the reviewer’s concerns and comments detailing the changes we have made in response to each point raised. The changes made to the manuscript have been highlighted in yellow based on the comments and points raised by the reviewer in the “Revised Manuscript with Track Changes”. We would like to thank the reviewer for taking the time to read our manuscript and provide thoughtful and helpful comments for revision. We believe the revised manuscript represents a significant improvement of the original one.

We look forward to hearing from you in due course.

Sorelle Sandie (on behalf of all authors)

See Authors’ responses to reviewer’s comments in the attached document.

**Responses to Reviewer’s Comments and Concerns**

We are submitting here responses to the concerns raised by the reviewer on manuscript: PONE-D-20-20708.

**Title**:

**Malaria and intestinal parasite co-infection and its association with anaemia among people living with HIV in Buea, Southwest Cameroon: A community-based retrospective cohort study**

**Authors**

Sorelle Mekachie Sandie*: [sandiesorelle2@gmail.com](mailto:sandiesorelle2@gmail.com)

Irene Ule Ngole Sumbele: [sumbelei@yahoo.co.uk](mailto:sumbelei@yahoo.co.uk)

Martin Mih Tasah: [tasahmartins@gmail.com](mailto:tasahmartins@gmail.com)

Helen Kuokuo Kimbi: [hkimbi@yahoo.co.uk](mailto:hkimbi@yahoo.co.uk)

Version: 6

Date: 25^th^ November 2020

Authors’ responses to reviewer’ concerns over leaf.

**Corrections and concerns of Reviewer #3:**

**Pg 3, 65-67: This statement is incorrect. I pointed this out in my previous review. Authors stated “microbial and parasitic infections such as…”. It is expected that the examples to be given would be microbial and parasitic infections, and not their aetiological agents. Plasmodium is not an infection but an agent, likewise Mycobacterium. If the authors insist on mentioning the aetiology of the infections, then I suggest “microbial and parasitic infections… “ be corrected to “microbes such as Plasmodium…”. Authors need not repeat parasites in this revision as microbes adequately captures this in the context.**

As suggested by the reviewer, the words “microbial and parasitic infections” have been replaced by “microbes such as *Plasmodium, Mycobacterium* and several intestinal parasites” to eliminate ambiguity (See lines 65-67).

**Pg 4, line 74-75: “African countries still bear the largest… “. Contrary to the authors’ reply to my previous comment, the most recent WHO world malaria report was published in April 2020. Please update information provided in this section.**

We thank the reviewer for the updated information. The information has been updated accordingly as suggested by the reviewer indicating that the most recently published malaria report is that of 2019 as published in April 2020 (See lines 73-74).

**Pg 6, line 127: Contrary to the authors’ reply to my comment, this assessment was concluded and announced in 2018. Also, summary sheet for this report is dated February 2020 and South West Region is not the 6th but the 7th according to the summary sheet. Please visit** [**https://phia.icap.columbia.edu/cameroon-summary-sheet/**](https://phia.icap.columbia.edu/cameroon-summary-sheet/) **to obtain this resource and revise this section of the manuscript accordingly, as well as the affected reference.**

We are grateful to the reviewer for providing the link with the most recent information on the position of the South West Region in Cameroon with respect to HIV ranking. The information has been updated r (See lines 125-126).

**Pg 6, line 136: Authors were required to specify the steps and assays used for the confirmation of HIV Status. This has not been done. Determine is not a confirmation assay, although it is employed as a national algorithm in certain countries alongside others serial assays for diagnostic purposes. Western blot and PCR are confirmatory assays used for confirmation of HIV infection. Did authors employ any of these in confirming the HIV Status of study participants? If yes, please give a detailed description of the assay methods employed under a subtitle**

Due to limited finances, no HIV confirmatory test was carried out in this study and the HIV positivity of the participants was based on positive results obtained using the Determine Test strip. The sentence has been rephrased for better understanding (See line 135).
