# Supplementary material for: Malaria and intestinal parasite co-infection and its association with anaemia among people living with HIV in Buea, Southwest Cameroon: A community-based retrospective cohort study
Source: PLoS One. 2021 Jan 22;16(1):e0245743. doi: 10.1371/journal.pone.0245743 (PMC7822292; doi:10.1371/journal.pone.0245743)
Supplement: Attachment — Submitted filename: Response to Reviewers.docx [file pone.0245743.s009.docx]

**Dear Editor**

Many thanks for your email of 3^rd^ December 2020 providing reviewer’s comments on our manuscript and inviting us to submit a revision. We have now prepared a revised manuscript reflecting the suggestions made by the reviewer for your consideration.

On behalf of the authors we wish to express our dismay at the language used by the current reviewer at this round of review process for a subject that had been adequately addressed in the first 5 rounds of review by the previous reviewers. I (Irene Sumbele; ORCID: 0000-0003-2607-8845) a co-author of this manuscript, a regular reviewer of PLoS articles and an Editor as well will like to call to the attention of the Editor to this issue. We would wish and appreciate if the current reviewer will appraise the context, objective and study design of the study. However, together with the corresponding author, we have provided a point by point responses to the concerns raised by the reviewer in this current round of review. See document overleaf. The changes made to the manuscript have been highlighted in yellow based on the comments and points raised by the reviewer in the “Revised Manuscript with Track Changes”.

We would like to thank the reviewer and the Editor for taking the time to read our manuscript and provide comments for revision.

We look forward to hearing from you in due course.

Sorelle Sandie (on behalf of all authors) and Irene Sumbele

See Authors’ responses to reviewer’s comments in the attached document.

**Responses to Reviewer’s Comments and Concerns**

We are submitting here responses to the concerns raised by the reviewer on manuscript: PONE-D-20-20708.

**Title**:

**Malaria and intestinal parasite co-infection and its association with anaemia among people living with HIV in Buea, Southwest Cameroon: A community-based retrospective cohort study**

**Authors**

Sorelle Mekachie Sandie*: [sandiesorelle2@gmail.com](mailto:sandiesorelle2@gmail.com)

Irene Ule Ngole Sumbele: [sumbelei@yahoo.co.uk](mailto:sumbelei@yahoo.co.uk)

Martin Mih Tasah: [tasahmartins@gmail.com](mailto:tasahmartins@gmail.com)

Helen Kuokuo Kimbi: [hkimbi@yahoo.co.uk](mailto:hkimbi@yahoo.co.uk)

Version: 7

Date: 31^st^ December 2020

Authors’ responses to reviewer’ concerns over leaf.

**Concerns or comments of the Editor**

**The reviewer strongly suggests the authors to adjust MS’s results otherwise the quality of the MS may be compromised.**

We the authors believe the results are presented in an appropriate manner to address the objective of the study which is to “determine the prevalence of malaria parasite and intestinal parasites in PLWH compared to HIV negative counterparts as well as to assess the effects of these infections on anaemia to provide relevant information to policymakers in the country’. The PLWH were recruited from HIV care and treatment center where testing is usually done following the National algorithm and the HIV negative individuals were recruited from the community upon confirmation of their HIV-negative status. This is the way the results have been presented following the input by the several reviewers. Hence, we the authors believe there is no need for further modification of the presentation of the results at this point since no further study was carried out or the objective of the study has not been changed or rephrased.

**Corrections and concerns of Reviewer #3:**

**Authors have done a commendable job in addressing my review comments. However, the methodology is heavily flawed due to the lack of confirmation of the HIV Status of the study participants.**

We the authors wish to vehemently reject the appraisal of “**methodology is heavily flawed due to lack of confirmation of HIV status”.** As stated in the methodology, PLWH were recruited from the HIV treatment and care centers where potential participants in the study were already enrolled members with the centers based on the fact that their HIV status had been confirmed based on the National algorithm in all the treatment and care centers in the national territory. Because of their HIV status, viral load and CD4 counts were regularly evaluated and it was confirmed that they were on anti-retrovirals for varying periods as indicated in the data. Hence, they were confirmed as PLWH. That is why the study design is a retrospective cohort study. For the healthy individuals recruited during the outreach program in the community, their HIV negative status was confirmed using the Determine 1 test which is actually stated in the algorithm that it is highly sensitive and used to confirm HIV negative cases (who lack the corresponding antibodies being detected).

**Where confirmation assays are not obtainable for one reason or the other, national algorithm is often employed and acceptable for research purposes.**

The status of all the participants were confirmed, that is why they were grouped into PLWH and HIV negative individuals. The four participants identified in the outreach program that were HIV positive for the Determine1, their confirmation test was obtained from the HIV treatment and care center following their enrolment based on the referral. We will wish to inquire if the reviewer is trying to introduce a new protocol by further confirmation of HIV negative test results of healthy individuals tested by Determine1 which is the standard National algorithm for those who are HIV negative?

**National algorithm usually involves serial testing (more than 1 test). Cameroon employs 2 RDTs in 2 or 3 serial tests, depending on whether Indeterminate results were obtained. With only one test (Determine) carried out, it is impossible to tell if there were any false positives/negatives obtained. I suggest that authors visit** [**https://bmjopen.bmj.com/content/8/3/e020611**](https://bmjopen.bmj.com/content/8/3/e020611) **to study the algorithm used and carry out the same methods in the present study to improve the quality of this study.**

We appreciate the fact that the reviewer referred us to this paper which we had consulted before designing and carrying out the study. It is based on the algorithms that PLWH were enrolled into the study from the HIV care and treatment center where the protocols are strictly followed before a patient is enrolled in the center.

The present study included both people living with HIV and HIV negative participants from the community. As specified in the methodology, HIV positive participants were recruited from the HIV Treatment and Care center of the hospital and therefore their positive HIV status had already been confirmed as prescribed by the Cameroon national algorithm i.e using Determine as the first line test and using Oraquick as the confirmatory test before placing them on ART. Determine 1 test was used in this study to confirm the negative HIV status of the HIV negative participants from the community before enrolment into the study and as the Determine test gave negative result, no confirmatory test was done as suggested by the national algorithm.

However, the statement on the HIV diagnosis has been rephrased to read “The two categories of participants, PLWH and HIV-negative individuals aged 1-72 years old were enrolled in the study and the HIV status of the HIV negative participants was determined using the Determine HIV Test strip.” to avoid misunderstanding. (See lines 134-135).

**Also, I suggest that the inability to carry out further confirmation assays be included in a section with the subtitle "Study limitations". Other challenges/limitations encountered in the course of the study can also be included in this section.**

We the authors believe that the “inability to carry out further HIV confirmatory test” is not a limitation, hence, should not be included in the “Study limitations” section. Based on the National algorithm, confirmatory test is definitely not a requirement for HIV negative participants tested by Determine 1. As for the 4 participants who were positive by Determine 1, their status was confirmed in the HIV Treatment and Care center following the standard operating procedure before they were included in the study. Hence the inability to carry out further confirmation is not a limitation as this was carried out.
