# Supplementary material for: Malaria and intestinal parasite co-infection and its association with anaemia among people living with HIV in Buea, Southwest Cameroon: A community-based retrospective cohort study
Source: PLoS One. 2021 Jan 22;16(1):e0245743. doi: 10.1371/journal.pone.0245743 (PMC7822292; doi:10.1371/journal.pone.0245743)
Supplement: Attachment — Submitted filename: Response to Reviewers.docx [file pone.0245743.s010.docx]

**Dear Editor**

Many thanks for your email of 05^th^ January 2021 providing reviewers’ comments on our manuscript and inviting us to submit a revision. We have now prepared a revised manuscript reflecting the suggestions made by the reviewer for your consideration. Below we have provided point-by-point response to the reviewer’s concerns and comments detailing the changes we have made in response to each point raised. The changes made to the manuscript have been highlighted in yellow based on the comments and points raised by the reviewer in the “Revised Manuscript with Track Changes”. We would like to thank the reviewer for taking the time to read our manuscript and provide thoughtful and helpful comments for revision. We believe the revised manuscript represents a significant improvement of the original one.

We look forward to hearing from you in due course.

Sorelle Sandie (on behalf of all authors)

See Authors’ responses to reviewer’s comments in the attached document.

**Responses to Reviewer’s Comments and Concerns**

We are submitting here responses to the concerns raised by the reviewer on manuscript: PONE-D-20-20708.

**Title**:

**Malaria and intestinal parasite co-infection and its association with anaemia among people living with HIV in Buea, Southwest Cameroon: A community-based retrospective cohort study**

**Authors**

Sorelle Mekachie Sandie*: [sandiesorelle2@gmail.com](mailto:sandiesorelle2@gmail.com)

Irene Ule Ngole Sumbele: [sumbelei@yahoo.co.uk](mailto:sumbelei@yahoo.co.uk)

Martin Mih Tasah: [tasahmartins@gmail.com](mailto:tasahmartins@gmail.com)

Helen Kuokuo Kimbi: [hkimbi@yahoo.co.uk](mailto:hkimbi@yahoo.co.uk)

Version: 8

Date: 05^th^ January 2021

Authors’ responses to reviewer’ concerns over leaf.

**Concerns or comments of the Reviewer:**

**Pg 6, 134 - Pg 7, 144: From the authors' response to my review comments, I understood that the PLWH enrolled in this study were already confirmed cases undergoing treatment. Such patients undergo testing at intervals (determined by the physicians) to ascertain if their proviral load is still detectable. According to the authors, the confirmation tests were carried out by the HAART centres before the authors enrolled the participants. If that was the case, I suggest that authors include a statement clarifying that all confirmation tests were already performed by the treatment centres on the study participants before they were enrolled in the study, and that the Determine test was carried out by the authors perhaps for quality control purposes. I recommend that the statement be included in Pg 7, line 141, immediately after "PLWH  identified  through  the  outreach  programmes  not registered  in  the  HIV  treatment  centre  were  referred  to  the  treatment  centre  for  confirmation  and follow-up."**

For better understanding, a statement on HIV confirmatory test carried out at the Treatment centre was included in Pg 7, line 139 – 141 as “PLWH identified through the outreach programmes in the community were referred to the HIV care and treatment centre for confirmation of their HIV positive status and follow up before being enrolled among the PLWH category”.
